# Temporal transcriptomic differences between tolerant and susceptible genotypes contribute to rice drought tolerance

**DOI:** 10.1186/s12864-020-07193-7

**Published:** 2020-11-10

**Authors:** Hui Xia, Xiaosong Ma, Kai Xu, Lei Wang, Hongyan Liu, Liang Chen, Lijun Luo

**Affiliations:** 1 Shanghai Agrobiological Gene Center, Shanghai, China; 2 School of Agriculture and Biology, Shanghai Jiao Tong University, Shanghai, China

**Keywords:** RNA-sequencing, Drought tolerance, Transcriptomic tradeoff, *Oryza sativa*

## Abstract

**Background:**

Drought-tolerance ensures a crop to maintain life activities and protect cell from damages under dehydration. It refers to diverse mechanisms temporally activated when the crop adapts to drought. However, knowledge about the temporal dynamics of rice transcriptome under drought is limited.

**Results:**

Here, we investigated temporal transcriptomic dynamics in 12 rice genotypes, which varied in drought tolerance (DT), under a naturally occurred drought in fields. The tolerant genotypes possess less differentially expressed genes (DEGs) while they have higher proportions of upregulated DEGs. Tolerant and susceptible genotypes have great differences in temporally activated biological processes (BPs) during the drought period and at the recovery stage based on their DEGs. The DT-featured BPs, which are activated specially (e.g. raffinose, fucose, and trehalose metabolic processes, etc.) or earlier in the tolerant genotypes (e.g. protein and histone deacetylation, protein peptidyl-prolyl isomerization, transcriptional attenuation, ferric iron transport, etc.) shall contribute to DT. Meanwhile, the tolerant genotypes and the susceptible genotypes also present great differences in photosynthesis and cross-talks among phytohormones under drought. A certain transcriptomic tradeoff between DT and productivity is observed. Tolerant genotypes have a better balance between DT and productivity under drought by activating drought-responsive genes appropriately. Twenty hub genes in the gene coexpression network, which are correlated with DT but without potential penalties in productivity, are recommended as good candidates for DT.

**Conclusions:**

Findings of this study provide us informative cues about rice temporal transcriptomic dynamics under drought and strengthen our system-level understandings in rice DT.

## Background

Water deficiency and drought is one of the most limiting and disastrous factors for plant adaptation and crop production. It is thus urgent for us to breed water-saving and drought-resistant crops [20, 21]. Among various mechanisms of drought resistance, drought tolerance (DT) ensures a plant to maintain its normal life activities under drought and protect plant cell away from damages of dehydration [8]. It is compromised by diverse mechanisms (e.g. stomata conductance, osmotic adjustment, and protective molecules), which are temporally activated when a plant adapts to a naturally-occurred drought [8, 29]. Briefly, the plant senses and transmits signals of water deficiency in first and then increases its osmolality to maintain turgor pressures by up-regulating various osmolytes [3, 8]. Sooner or later, the plant represents various acclimation responses (e.g. closure of stomata, leaf rolling, decrease in photosynthesis, inhibitions of growth and development) to reduce water loss and consumption [6, 11, 30, 43]. It needs to activate protective mechanisms, such as antioxidant enzymes and molecular chaperones, to protect cell from damages of dehydration at middle-later drought [31, 44]. Finally, cell death occurs when the degree of dehydration exceeds its tolerance, representing as leaf senescence and/or fertile abortion [27, 32]. The temporal physiological responses of a plant during drought should be determined by the regulation of relevant genes, which activates certain biological processes appropriately. Therefore, learning temporal transcriptomic dynamics of a plant adapting to a long-term drought can deepen our understanding in drought tolerance.

Rice is one of the most important cereal crops which provides > 50% stable food for global populations. The rice cultivation costs huge amount of water and is very sensitive to drought [2]. Water-saving and drought-resistance rice is thus required to ensure global food safety [21]. However, drought tolerance in rice is composed by thousands of genes with minor effects [10]. Hundreds of genes have been reported to be involved in plant responses to drought stress, forming a complicated gene network [10]. It is therefore the improvement of drought tolerance by manipulating on a single gene has limited success in the field, although many drought-tolerant genes have been reported to have significant effects in the laboratory or small-scale field conditions. A systematic solution in gene engineering or breeding to improve drought tolerance in rice is required.

The methodology of system biology, such as transcriptomics, is a powerful tool to explain complex traits. Many comparative transcriptomic studies on rice drought tolerance have been published in the last decade, contributing a lot in our understandings of drought tolerance at the systematic level [4, 5, 14, 18, 23–26, 34, 40, 46]. However, there are still several limitations remaining. First, the former studies preferred to use a single genotype [24, 25, 40] or a pair of genotypes with contrasting drought tolerance [4, 5, 23]. This may introduce genotype-specific bias. Second, drought tolerance in a crop requires stable productivity under drought rather than mere survival. In that case, many acclimation responses (e.g. stomata closure, leaf rolling, decrease in photosynthesis, etc.) inappropriately activated under normal conditions may lead to agronomic penalties. As a result, tradeoffs between DT and productivity have been frequently reported at the gene [15, 45], genome [39], transcriptome [42], and population scales [22, 41]. For the better understanding and utilization of drought-tolerant genes, such potential tradeoffs between drought tolerance and productivity in a crop should be seriously evaluated at the transcriptomic level. Finally, a comparative transcriptomic study can commonly obtain large number of differentially expressed genes (DEGs), most of which are byproducts accompanying with the plant’s morphological and physiological changes. In fact, only a few candidate genes from transcriptomic studies have been functionally characterized, according to the large number of candidates proposed by these transcriptomic studies on drought tolerance. How to effectively isolate the cause gene of DT from the large number of drought-responsive genes (DRGs) is still a challenge for researchers.

In this study, twelve rice genotypes (six tolerant and six susceptible genotypes) were used to investigate their transcriptomic dynamics during a long-term progressive drought. The drought treatment is arranged in a modified field, where drought-avoidance by root systems is separated from drought tolerance by the design of shallow soil layers [23]. The involvement of enough genotypes could mitigate genotype-specific bias in generating drought-responsive genes (DRGs). Meanwhile, the temporal differences between tolerant and susceptible genotypes in the activated biological processes could provide valuable information to determine key biological processes and genes that contribute to drought tolerance. In addition, the weighted gene coexpression network analysis (WGCNA) are applied to extract modules associated with drought-tolerant traits and to identify candidate genes with systematic impacts on DT. We aimed to disclose the transcriptomic dynamics in rice adaptation to the long-term drought and explore the cause of drought tolerance at the transcriptomic level. It can provide novel insights into the system-level understanding of drought tolerance.

## Results

### Influences of the long-term progressive drought on growth, physiological traits, and agronomic traits in rice genotypes

The soil-water content was decreased from 21.4 to 10.8% during the drought-period (Additional file 1: Figure S1). It caused dead leaves (ranged from 0.241 to 0.509) and significant reductions in biomass and yield among rice genotypes (Table 1). The drought-tolerant index based on the relative biomass (DTIB) ranged from 0.261 to 1.058 (Table 1). We could then divide the twelve genotypes into two groups of contrasting DTs based on their DTIBs_._ Lower values of DTIB (0.352 ± 0.040) in the susceptible group mean these genotypes may have greater acclimation responses. In contrary, higher values of DTIB in the tolerant group (0.862 ± 0.080) indicated well maintained life activities under drought. We also measured the traits relevant to osmotic adjustment and anti-oxidization. The osmotic potentials in susceptible and tolerant groups were almost the same under well-watered conditions (W) (Table 2). However, the osmotic potential of the tolerant group was significantly higher than that of the susceptible group during the drought period and recovery (R) stages (Table 2). The content of H_2_O_2_ in the tolerant group was significantly lower than that in the susceptible under both W and drought (D) conditions (Table 2). These results indicated osmotic adjustment and anti-oxidant capacities played essential roles in rice DT.

**Table 1:** Agronomic traits (mean ± standard deviation) and drought-tolerance index (DTI) evaluated on twelve rice genotypes under well-watered (W) and drought (D) conditions. “*”, “**”, and “***” indicate significant differences between W and D at *p* < 0.05, *p* < 0.01, and *p* < 0.001 via independent t test. DTIB or DTIY is calculated as P_d_/P_W_*(P_d_/P_ad_). P_d_ is referred as biomass/yield in D, P_w_ is referred as biomass/yield in W, and P_ad_ is referred as averaged biomass/yield of total rice genotypes in D

Code	Materials	Ratio of dead leaf	Seed set rating in W (%)	Seed set rating in D (%)	Biomass in W (g)	Biomass in D (g)	Yield per plant in W (g)	Yield per plant in D (g)	Harvest index in W	Harvest index in D	DTIB	DTIY
S3	IAC1246^b^	0.241	68.0 ± 1.4	80.4 ± 6.1	28.35 ± 2.00	19.64 ± 0.75*	10.65 ± 0.32	7.89 ± 0.42**	0.402 ± 0.025	0.378 ± 0.029	1.053	1.680
S6	Hanhui-3^b^	0.359	51.0 ± 0.8	23.7 ± 4.5**	27.60 ± 1.99	19.23 ± 1.16*	7.61 ± 0.29	1.17 ± 0.30***	0.063 ± 0.036	0.278 ± 0.035***	1.037	0.052
S9	CICA4^a^	0.410	69.2 ± 2.6	56.0 ± 10.6	13.67 ± 1.16	8.03 ± 0.73*	6.34 ± 0.54	2.03 ± 0.53**	0.244 ± 0.082	0.463 ± 0.012**	0.365	0.186
S11	Aimi ^b^	0.405	76.9 ± 8.0	82.8 ± 2.9	25.11 ± 3.64	17.56 ± 1.73*	11.17 ± 0.39	7.82 ± 0.50**	0.448 ± 0.027	0.450 ± 0.050	0.950	1.571
S12	Zhonghan-3^a^	0.435	79.3 ± 1.9	67.8 ± 4.9	19.78 ± 1.01	8.50 ± 0.63**	9.49 ± 0.47	2.45 ± 0.12***	0.289 ± 0.015	0.480 ± 0.024***	0.283	0.181
S14	Yunlu^a^	0.509	73.2 ± 2.3	81.7 ± 2.5	19.70 ± 2.73	11.34 ± 0.62**	7.41 ± 0.50	3.31 ± 0.15***	0.292 ± 0.005	0.378 ± 0.022*	0.505	0.425
S17	Qingsizhan ^b^	0.416	88.5 ± 1.8	47.8 ± 3.6**	20.79 ± 0.79	13.69 ± 1.60*	10.49 ± 0.65	2.05 ± 0.20***	0.151 ± 0.023	0.504 ± 0.022***	0.698	0.115
S18	IRAT109 ^a^	0.468	77.2 ± 6.9	52.8 ± 13.0	16.45 ± 2.30	7.90 ± 1.00*	8.56 ± 1.45	1.84 ± 0.49*	0.252 ± 0.169	0.517 ± 0.059**	0.293	0.113
S24	IPECA0162 ^a^	0.366	87.7 ± 2.8	54.5 ± 14.8	14.20 ± 0.27	6.93 ± 0.64***	7.26 ± 0.34	1.51 ± 0.50**	0.235 ± 0.166	0.511 ± 0.034**	0.261	0.090
S26	Bulebelle ^a^	0.435	70.9 ± 0.5	60.7 ± 4.9	18.17 ± 1.10	10.21 ± 0.91*	9.27 ± 0.74	4.26 ± 0.67**	0.413 ± 0.050	0.509 ± 0.018	0.444	0.563
S28	Tresmes ^b^	0.441	84.0 ± 2.7	64.3 ± 14.4	29.88 ± 0.26	18.75 ± 2.60*	13.30 ± 0.33	4.76 ± 0.96**	0.270 ± 0.137	0.445 ± 0.015*	0.911	0.488
S31	Huhan-1B ^b^	0.384	74.6 ± 0.7	43.8 ± 2.2***	26.14 ± 1.49	13.32 ± 0.96**	11.76 ± 0.44	2.71 ± 0.20***	0.204 ± 0.007	0.451 ± 0.029**	0.525	0.179

^a^ indicates susceptible genotype

^b^ indicates tolerant genotype

**Table 2:** Osmotic potential and H_2_O_2_ content (Mean ± SE) measured during drought (D1-D5) and at recovery (R) in the tolerant and the susceptible groups from well-watered (W) and drought (D) fields. Different letters indicate significant differences by one-way ANOVA (SNK method) during drought period (D1-D5). “*” indicates significant difference at *p* < 0.05 by independent *t*-test between the tolerant and the susceptible groups at the recovery stage

Trait	Group	Field	Time point					
			D1	D2	D3	D4	D5	R
Osmotic potential	Susceptible	Drought-treated	493.8 ± 11.6a	590.2 ± 15.7bc	498.2 ± 10.8b	595.5 ± 14.9c	648.4 ± 6.3b	593.3 ± 16.7*
	Susceptible	Well-watered	494.7 ± 8.1a	530.2 ± 14.2a	419.2 ± 20.9a	504.2 ± 11.1a	524.8 ± 9.9a	NA
	Tolerant	Drought-treated	536.9 ± 11.8b	621.8 ± 15.8c	548.2 ± 21.2c	673.2 ± 14.4d	708.1 ± 15.8c	641.1 ± 15.8*
	Tolerant	Well-watered	545 ± 10.3b	565.7 ± 13.1ab	435.8 ± 10.8a	545.9 ± 14.7b	547.5 ± 10.7a	NA
H2O2 content (mmol/g FW)	Susceptible	Drought-treated	0.403 ± 0.054b	0.594 ± 0.112a	0.583 ± 0.101b	0.539 ± 0.09b	0.654 ± 0.126b	0.576 ± 0.096*
	Susceptible	Well-watered	0.358 ± 0.061b	0.532 ± 0.117a	0.354 ± 0.057a	0.41 ± 0.078ab	0.453 ± 0.093ab	NA
	Tolerant	Drought-treated	0.199 ± 0.019a	0.292 ± 0.036a	0.287 ± 0.018a	0.301 ± 0.028a	0.353 ± 0.02a	0.334 ± 0.012*
	Tolerant	Well-watered	0.167 ± 0.014a	0.318 ± 0.054a	0.224 ± 0.042a	0.243 ± 0.01a	0.29 ± 0.05a	NA

### Summary of differentially expressed genes (DEGs) detected in twelve rice genotypes at six time points

The averaged total base for each sample was 4.48*10^9^ bases with the uniquely mapped rate of 87.0%. The Q30 rate was as high as 92.6% (Additional file 2: Table S1). There were 49,470 genes annotated to the reference genome MSU7.0 totally detected to be expressed in this study (FPKM> 0.001), ranging from 33,351 to 42,130 among 12 rice genotypes (Additional file 2: Table S2). Among these expressed genes, 14,354 genes were determined as DEGs at least once among all rice genotypes throughout six experimental time points (D_1_ to D_5_, and R), covering 99 known drought-tolerant genes (Additional file 1: Figure S2a, Additional file 2: Table S3).

**Fig. 1:**
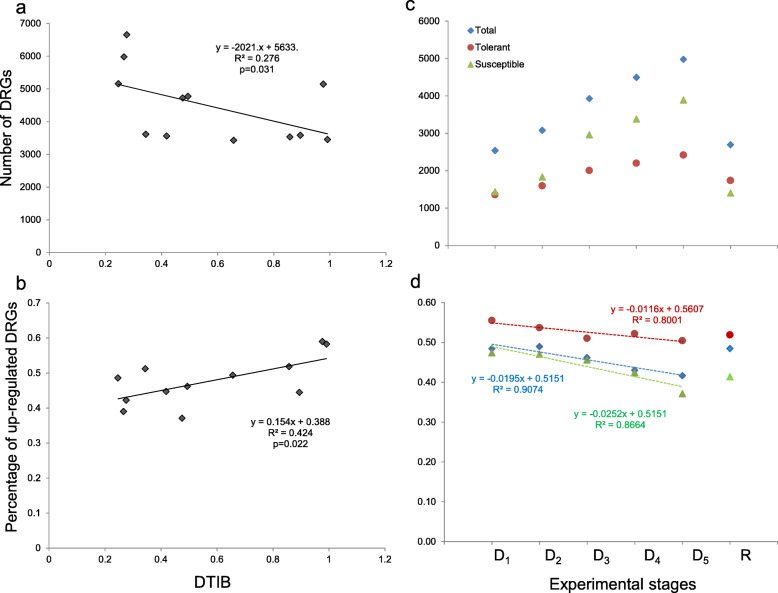
Number of drought-responsive genes (DRGs) and the percentage of up-regulated DRGs are associated with drought-tolerance. **a** Correlation of number of DRGs with drought-tolerance index by biomass (DTIB). **b** Correlation of percentage of up-regulated DRGs with DTIB. **c** Number of DRGs detected in total, tolerant, and susceptible genotypes at six time points. **d** Percentage of up-regulated DRGs in total, tolerant, and susceptible genotypes at six time points

For each rice genotype, the number of DEGs detected during drought period (D_1_ to D_5_) (defined as drought-responsive genes, DRGs) ranged from 3432 to 6660 (Additional file 1: Figure S3a). The averaged ratio of common DRGs shared by any two rice genotypes was 34.9 ± 6.0% (mean ± SD), suggesting a great genotype-specific manner in rice response to drought. Interestingly, the tolerant genotypes and the susceptible genotypes could be separately clustered by their presence or absence of total detected DRGs (Additional file 1: Figure S3a), indicating distinguished transcriptomic bases between the two groups. Meanwhile, the number of DRGs detected in each genotype was negatively correlated with its DTIB (Fig. 1a). It was also noteworthy that the DTIB was positively correlated with the proportion of upregulated DRGs among rice genotypes (Fig. 1b), indicating positive roles played by upregulated DRGs in DT. Based on the GO enrichment, upregulated and downregulated DRGs were relevant to different classifications of biological processes (Additional file 2: Table S4). For example, upregulated DRGs tended to be relevant to “carbohydrate metabolic process”, “response to abiotic stimulus”, “cellular component organization and biogenesis”, and “cell homeostasis”, while downregulated DRGs tended to be relevant to “response to external/extracellular stimulus”, “nucleotide and nucleic acid metabolic process”, “cell death”, “photosynthesis” and “generation of precursor metabolites and energy” (Additional file 2: Table S4).

To avoid positive false of our transcriptomic data, only the gene represented as a DEG at least in two genotypes at a time point were chosen for further analysis, resulting in 8270 DEGs in the final dataset (Additional file 1: Figure S2b). The excluded 6084 genes (accounting for 42.3%) conferred 13 function-characterized drought-tolerant genes (Additional file 1: Figure S2b), indicating only a limited potential loss (13.3%) of valuable information by this exclusion. Meanwhile, the mean absolute Log_2_^FC^ values of excluded DEGs (frequency = 1) were less than 1.0 at all six time points (Additional file 1: Figure S4).

### Cluster analysis of rice genotypes under W or D conditions based on expression of total genes and DRGs

Cluster based on the FPKMs of total expressed genes could not separate samples from D and W/ or D and recovery (R) at all six time points (Additional file 1: Figure S5), while cluster based on the FPKMs of DEGs could majorly separate D samples from W/R samples along with the progressive drought (Additional file 1: Figure S6). Moreover, the tolerant genotypes and the susceptible genotypes could be majorly separated by their FPKMs of DRGs under drought (Additional file 1: Figure S6). It was still necessary to point out that the susceptible and tolerant genotypes could be well separated by the cluster analysis based on their SNPs called from total transcripts (Additional file 1: Figure S7a) or called from transcripts of DEGs (Additional file 1: Figure S7b). These results allowed us to combine transcriptomic data of tolerant or susceptible genotypes together to form a tolerant or a susceptible group.

### Differences in DEGs detected in tolerant and susceptible genotypes at six time points

There were 1444–3893 DRGs in the susceptible group during the drought period and 1358–2410 recovery related genes (RRGs) at the recovery stage (Additional file 1: Figure S3b, Fig. 1c). The percentage of upregulated DRGs was gradually decreased along with the progressive drought (Fig. 1d). As expected, the tolerant group contains more upregulated DRGs. Based on the presence (1) and absence (0) of DEGs in the tolerant group and the susceptible group, they could not be clearly separated at the early drought (D1 and D2), while they were separated at the later drought stages (D3, D4, and D5) along the first and second coordinates (Additional file 1: Figure S8a). However, they were not well separated at the R stage based on the presence and absence of RRGs,

There were 3355 common DRGs shared by the tolerant group and the susceptible group during drought period, accounting for 57.8% of total DRGs. There were 2476 susceptible-specific and 1171 tolerant-specific DRGs (Additional file 1: Figure S9). The tolerant-specific DRGs tended to be relevant to catabolic process, carbohydrate metabolic process, and cellular component organization and biogenesis (Additional file 1: Figure S10). The susceptible-specific DRGs tended to be relevant with lipid metabolic process, protein metabolic process, nucleotide and nucleic acid metabolic process, and protein modification process (Additional file 1: Figure S10). Meanwhile, we also detected 605 DRGs having significant differences in fold changes between the tolerant group and the susceptible group (defined as the tolerant group and the susceptible group different DRGs, TS-different DRGs). In most cases (84.6%, 918 in 1085), higher absolute log_2_FC values of these TS-different DRGs were observed in the tolerant group. They were relevant to GO classifications of response to abiotic stimulus, response to stress, carbohydrate metabolic process, catabolic process, and secondary metabolic process (Additional file 1: Figure S10). At recovery, we detected 589 susceptible-specific and 920 tolerant-specific RRGs (Additional file 1: Figure S9).

### Temporal regulation of DEGs in the tolerant group and the susceptible group during drought and at recovery

The time-series regulation of DRGs could form 16 modes (clusters) respectively in the tolerant (Additional file 1: Figure S11) and susceptible (Additional file 1: Figure S12) groups during the drought period. Most of the common DRGs (2757, 82.2%) between the two groups represented similar patterns of time-series regulations (positively correlated, PPC > 0.4), while only 84 (2.5%) common DRGs represented contrary patterns of time-series regulations (negatively correlated, PCC < -0.4) (Additional file 2: Table S5). The distribution of osmotic-correlated or H_2_O_2_-correlated DRGs in the 16 modes was not random (Additional file 1: Figure S11, S12). Osmotic-correlated DRGs in the susceptible group majorly distributed in mode-1, 2, 5, 7, and 13, while these of the tolerant group majorly distributed in mode-1, 2, 3, 6, 7, 8, and 11. H_2_O_2_-correlated DRGs in the susceptible group majorly distributed in mode-1 and 3, while these of the tolerant group majorly distributed in mode-1 and 2.

The regulation of RRGs during the recovery process (W_5_-D_5_-R) formed eight modes (Additional file 1: Figure S13a). There were 1463 RRGs representing similar modes of time-series regulation between susceptible and tolerant groups, accounting for 66.9% total common RRGs (Additional file 1: Figure S13b). RRGs belonging to the same mode had similar biological functions as revealed by the PCoA based on enriched GOBPs (Additional file 1: Figure S13c). We thought RRGs in cluster-7 play key roles in recovery as they were specifically activated (upregulated) during the recovery process. RRGs of cluster-7 had great differences in enriched GO biological processes between the tolerant group and the susceptible group (Additional file 2: Table S6, Additional file 1: Figure S13c), providing additional evidence for its important roles in drought recovery.

### Temporal differences in biological processes between the tolerant group and the susceptible group

There were 356 GOBPs totally enriched (FDR < 0.05) based on DEGs in the tolerant group and the susceptible group at six time points (Additional file 1: Figure S14). During drought period, 78, 80, 66, 29, 56, and 46 GOBPs belonged to Type I, Type II, Type III, Type IV, Type V, and Type VI, respectively (Additional file 1: Figure S14). GOBPs of Type I were commonly enriched by tolerant and susceptible genotypes, suggesting their universal roles in responses to drought. They were majorly relevant to transport, carbohydrate metabolic process, secondary metabolic process, response to endogenous stimulus, response to abiotic stimulus, and response to biotic stimulus. GOBPs of Type II and Type IV could be considered as the DT-featured BPs. They tended to be relevant to nucleotide and nucleic acid metabolic process (GO:0006139), catabolic process (GO:0009056), generation of precursor metabolites and energy (GO:0006091), cellular component organization and biogenesis (GO:0016043), cell communication (GO:0007154), post-embryonic development (GO:0009791), response to extracellular stimulus (GO:0009991), and carbohydrate metabolic process (GO:0005975) (Additional file 2: Table S7). In contrast, GOBPs of Type III and V could be considered as susceptibility-featured BPs. They tended to be relevant to lipid metabolic process (GO:0006629), secondary metabolic process (GO:0019748), response to external stimulus (GO:0009605), response to endogenous stimulus (GO:0009719), and response to biotic stimulus (GO:0009607) (Additional file 2: Table S7). Finally, there were 179 GOBPs enriched at the recovery stage by the tolerant and the susceptible groups, ten among which were recovery-specific, referring to growth (GO:0040007), reproductive process (GO:0022414), metal ion homeostasis (GO:0055065), developmental process involved in reproduction (GO:0003006), etc. (Additional file 1: Figure S14).

**Fig. 2:**
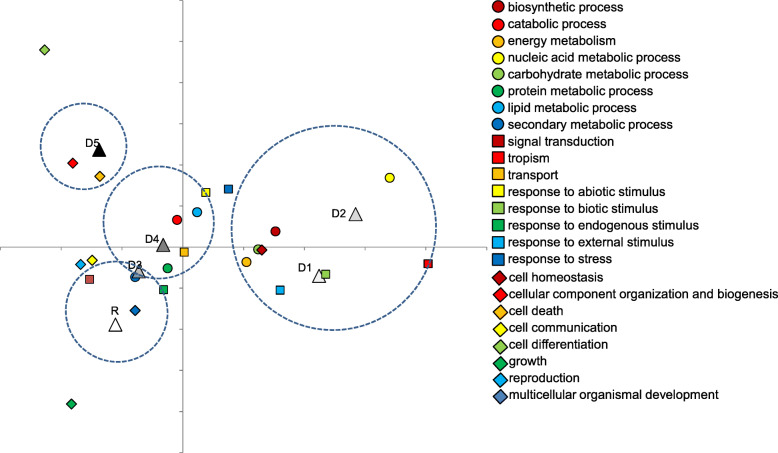
Description of temporal differences in GO biological processes between the tolerant and the susceptible groups during drought period (D1-D5) and at recovery (R) by correspondence analysis

Based on the enriched GOBPs, the tolerant group and the susceptible group could be separated along with the first coordinate in the PCoA, particularly at the later drought (D_3_-D_5_) (Additional file 1: Figure S8b). It indicated the tolerant group and the susceptible group had temporal differences in the activated biological processes in responses to drought, which varied at six experimental time points. The temporal differences in activated biological processes could be revealed by the correspondence analysis (Fig. 2). Early differences (D_1_ and D_2_) in biological processes between the tolerant group and the susceptible group mainly referred to GO classifications of nucleic acid metabolic process (GO:0006139), carbohydrate metabolic process (GO:0005975), generation of precursor metabolites and energy (GO:0006091), tropism (GO:0009606), response to external stimulus (GO:0009605), response to biotic stimulus (GO:0009607), cell homeostasis (GO:0019725), and biosynthetic process (GO:0009058). Differences at middle drought (D_3_ and D_4_) mainly referred to protein metabolic process (GO:0019538), lipid metabolic process (GO:0006629), secondary metabolic process (GO:0019748), catabolic process (GO:0009056), response to endogenous stimulus (GO:0009719), and transport (GO:0006810). Later differences (D_5_) mainly referred to cellular component organization and biogenesis (GO:0016043) and cell death (GO:0008219) (Fig. 2). It was noteworthy that regulation of programmed cell death (GO:0043067) and negative regulation of cell death (GO:0060548) were uniquely enriched in the susceptible group in the middle and later drought (Additional file 1: Figure S14). Differences in GOBPs at the recovery stage mainly referred to growth (GO:0040007), multicellular organismal development (GO:0007275), signal transduction (GO:0007165), and secondary metabolic process (GO:0019748) (Fig. 2, Additional file 1: Figure S14).

### Temporal differences in basic metabolic processes, transporters, and photosynthesis between tolerant and susceptible genotypes

Among 91 enriched GOBPs in the classification of carbohydrate metabolic process (*p* < 0.05), 78 had temporal differences between the tolerant group and the susceptible group (Additional file 1: Figure S15). Particularly, metabolic processes related to gluconeogenesis (GO:0006094), hexose (GO:0019319), raffinose (GO:0034484, GO:0033530), fucose (GO:0042353, GO:0006004), trehalose (GO:0005993), and rhamnogalacturonan (GO:0010400, GO:0010395) were belonged to Type II or Type IV during the drought period (Additional file 1: Figure S15). Similarly, many lipid (e.g. steroid esterification (GO:0034433), steroid catabolic process (GO:0006706), sterol esterification (GO:0034434), sterol catabolic process (GO:0016127), etc.) (Additional file 1: Figure S16) and protein metabolic processes (e.g. regulation of histone modification (GO:0031056), regulation of histone deacetylation (GO:0031063), regulation of protein deacetylation (GO:0090311), protein peptidyl-prolyl isomerization (GO:0000413), peptidyl-proline modification (GO:0018208) etc.) (Additional file 1: Figure S17) could be considered as DT-featured processes. Meanwhile, many metabolic processes of nucleobase, nucleoside, nucleotide and nucleic acid were uniquely enriched at D_2_ in the tolerant group (Additional file 1: Figure S18). It reflected more activated transcriptional activities in tolerant genotypes at early drought. Among various transporters, ferric iron import (GO:0033216), ferric iron transport (GO:0015682), sulfate transport (GO:0008272), sulfur compound transport (GO:0072348), tryptophan transport (GO:0015827), etc. were belonged to Type II and IV (Additional file 1: Figure S19). However, there was no apparent DT-featured secondary metabolic process during the drought period (Additional file 1: Figure S20). At the recovery stage, we also found some DT-specific processes, such as disaccharide catabolic process (GO:0046352), fucose metabolic process (GO:0006004), fucose biosynthetic process (GO:0042353) (Additional file 1: Figure S15), carotenoid biosynthetic process (GO:0016117) (Additional file 1: Figure S16), glucosinolate metabolic process (GO:0019760), glycosinolate metabolic process (GO:0019757) (Additional file 1: Figure S18), and many transports (Additional file 1: Figure S19). Based on the expression of photosynthesis-relevant genes, the tolerant genotypes and the susceptible genotypes were almost separately clustered during the drought or at the recovery stage (Additional file 1: Figure S21), while they could not clearly be separated under well-watered conditions (Additional file 1: Figure S22). The photosynthesis-relevant genes in the tolerant group were upregulated or slightly decreased while those in the susceptible group were greatly downregulated throughout the drought period, particularly at D_1_ and D_2_ (Additional file 1: Figure S23).

At the recovery stage, there were 15, 26, 4, 13, 47, and 6 GOBPs in the classification of carbohydrate (Additional file 1: Figure S15), lipid (Additional file 1: Figure S16), protein (Additional file 1: Figure S17), nucleic acid (Additional file 1: Figure S18), transporter (Additional file 1: Figure S19), and secondary metabolic process (Additional file 1: Figure S20) representing differences between the tolerant group and the susceptible group. The photosynthesis recovered more quickly in the tolerant group, reflected by its higher upregulations of photosynthesis-relevant genes at the recovery stage (Additional file 1: Figure S23).

### Cross-talks among phytohormones in tolerant and susceptible genotypes

The proportion of DRGs in total genes in the metabolic pathway of eight phytohormones ranged from 34.5 to 52.4% (Table 3). The occupation of group-distinguished genes (susceptible-specific, tolerant-specific, and TS different) ranged from 22.2 to 60.0% (Table 3). The regulation of ABA-relevant genes (80.0%, 8 out of 10) were mainly upregulated during drought period, while GA- (77.3%, 17 out of 22), auxin- (37.1%, 13 out of 35), and ethylene-relevant (48.1%, 26 out of 54) genes were mainly downregulated (Additional file 1: Figure S24). The regulation of other phytohormone-relevant genes varied among time-points and groups (Additional file 1: Figure S24). We can find that phytohormones relevant to growth and development (auxin, BR, and GA) have higher proportion of group-distinguished genes (Table 3). In contrast, the widely acknowledged stress-response phytohormone, ABA, had the lowest proportion of group-distinguished genes (20.0%, 2 out of 10) (Table 3). This result indicated that differences in the drought acclimation should contribute to their differences in rice DT. In addition, we observed a better coordination among JA, SA, BR, auxin, cytokinin, and ethylene in the tolerant genotypes, reflected by higher averaged PCC values among genes relevant to these phytohormones (Additional file 1: Figure S25).

**Table 3:** Description of drought-responsive genes (DRGs) in the metabolic pathway of eight phytohormones

Phytohormone	No. of total genes	No. of DRGs	No. of group-distinguished genes			Proportion of DRGs in total genes	Proportion of group-distinguished genes in DRGs
			Tolerant-susceptible different	Susceptible-specific	Tolerant-specific		
Abscisic acid	29	10	1	1	0	0.345	0.200
Auxin	93	35	4	12	4	0.376	0.571
Brassinosteroids	12	5	0	3	0	0.417	0.600
Cytokinin	28	11	1	2	2	0.393	0.455
Ethylene	148	54	6	9	6	0.365	0.389
Salicylic acid	68	33	8	6	1	0.485	0.455
Gibberellin	42	22	3	7	1	0.524	0.500
Jasmonic acid	46	15	1	5	1	0.326	0.467
Overall	428	168	20	43	14	0.393	0.458

### Correlations of DRGs with measured DT traits

There were 183 DRGs negatively correlated (Pearson correlation coefficient > 0.4, *p* < 0.001) with H_2_O_2_ content, while 383 DRGs positively correlated with it. Meanwhile, there were 459 DRGs negatively correlated with osmotic potential while 1292 DRGs positively correlated with it. The overlap between H_2_O_2_-correlated and osmolality-correlated DRGs was very rare (Additional file 1: Figure S26a). As expect, absolute values of Log_2_(fold change) of shared H_2_O_2_-correlated and osmolality-correlated DRGs were significantly higher in the tolerant group than those in the susceptible group (Additional file 1: Figure S27). Twenty-three GOBPs were enriched by H_2_O_2_-correlated DRGs (Additional file 2: Table S8), which mainly referred to GO classifications as metabolic process, cellular process, and transport (Additional file 2: Table S9). Among these enriched GOBPs, energy coupled proton transport (down electrochemical gradient) and ATP synthesis coupled proton transport were tolerant-specific (Type II) (Additional file 2: Table S8). Meanwhile, 136 GOBPs were enriched by osmolality-correlated DRGs (Additional file 2: Table S8), which mainly referred to GO classifications of carbohydrate metabolic process, biosynthetic process, nucleotide and nucleic acid metabolic process, etc. (Additional file 2: Table S9). Among these enriched GOBPs, 13 and 14 GOBPs were of type II and Type IV, respectively (Additional file 2: Table S8).

**Fig. 3:**
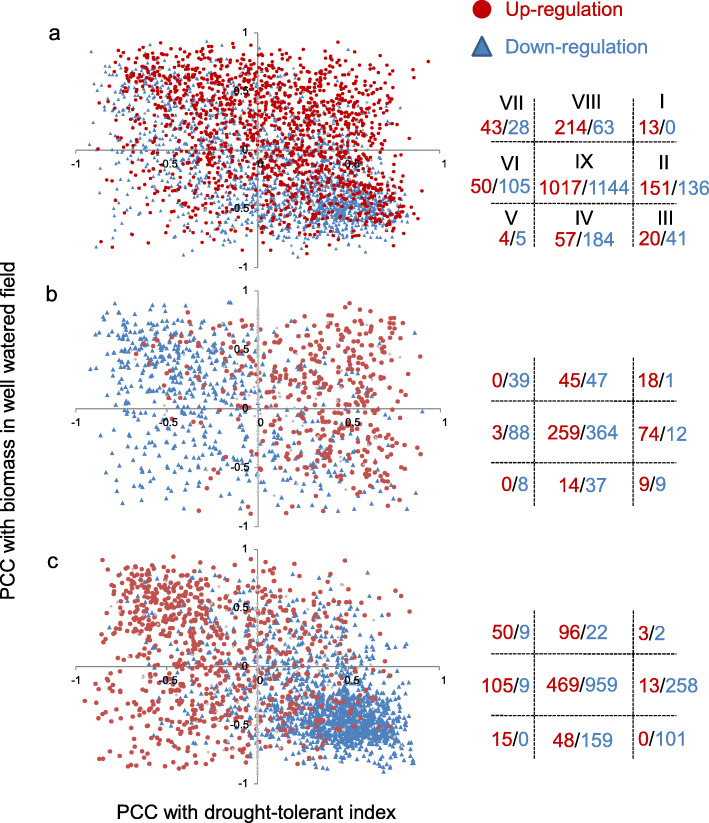
Transcriptomic tradeoffs between biomass and drought tolerance. **a** Correlations (Pearson correlation coefficient, PCC) of common drought-responsive genes (DRGs) with biomass under the well-watered condition (W-biomass) and drought-tolerant index by biomass. **b** Correlations of tolerant-specific DRGs with biomass under the well-watered condition and drought-tolerant index by biomass. **c** Correlations of susceptible-specific DRGs with biomass under the well-watered condition and drought-tolerant index by biomass (DTIB). DRGs in region I, III, V, and VII are significantly (*p <* 0.05) correlated with both W-biomass and DTIB. DRGs in region II and VI are significantly (*p <* 0.05) correlated with DTIB. DRGs in IV and VIII are significantly (*p <* 0.05) correlated with W-biomass. DRGs in region IX are not correlated with W-biomass or DTIB. The red/blue numbers in the right indicate number of up-regulated/down-regulated DRGs in the region

There were 1463 DRGs significantly correlated with the drought-tolerant index (DTI) (|PCC| > 0.576, *p* < 0.05) while there were 1403 DRGs significantly correlated with the biomass in the well-watered condition. Among 590 DRGs both correlated with DTI and biomass, a large proportion (499, 81.1%) located at the region of III and VII (Fig. 3), indicating their potential contradictory effects on DT and growth. This result indicated a gene, which is beneficial to DT, may potentially bring negative effects to growth. Interestingly, the tolerant- (Fig. 3b) and susceptible-specific (Fig. 3c) DRGs had opposite patterns in regulating DT and growth (Fig. 3b, c). In addition, a considerable proportion of H_2_O_2_- and osmolality-correlated genes were also correlated with biomass-W (Additional file 1: Figure S26b). This indicated DT mechanisms of ROS scavenging and osmotic adjustment can also have impacts on rice growth.

### Coexpression network and candidate hub genes for drought-tolerance and drought-recovery

Based on the result of WGCNA, nine biologically significant modules were generated when the cutoff threshold of edges was set at 0.16, which contained 52 to 3330 nodes (Table 4). The eigengene of the turquoise module was negatively correlated with the osmotic potential, while the eigengenes of the brown and purple modules were positively correlated with the osmotic potential (Table 4). This result indicated potential associations of the three modules with osmotic adjustment. The eigengenes of the tan, turquoise, yellow, and green modules were negatively correlated with the H_2_O_2_ content, while the eigengenes of brown and magenta modules were positively correlated with the H_2_O_2_ content (Table 4). This result indicated the potential associations of above modules with ROS scavenging. Meanwhile, each module was involved in many DT-featured biological processes based on the result of GO enrichment (Additional file 2: Table S10). For instance, many carbohydrate transporters, such as D-ribose transport (GO:0015752), pentose transport (GO:0015750), D-xylose transport (GO:0015753), sorbitol transport (GO:0015795), mannitol transport (GO:0015797), and dicarboxylic acid transport (GO:0006835), were enriched by genes in the brown module. The regulation of these transporters shall contribute to the osmotic adjustment (Additional file 2: Table S10). Meanwhile, the involvement in NADP biosynthetic process (GO:0006741) of the yellow module could be the potential explanation for its correlation with the H_2_O_2_ content (Additional file 2: Table S10). It is also noteworthy that the upregulated ABA relevant genes were mainly distributed in the blue and brown module (Additional file 2: Table S11), indicating that the association of the two modules with DT following the ABA-dependent manner. Other six phytohormones (ET, SA, auxin, JA, KT, and GA) were mainly distributed in the turquoise module (Additional file 2: Table S11), whose nodes were mainly downregulated (Table 4). It indicated the turquoise module played a role in the acclimation response.

**Table 4:** Description of nine modules in the weighted gene coexpression network. PCC is the abbreviation of Pearson’s correlation coefficient. *. **, and *** indicate the eigengene is significantly correlated with the trait at *p <* 0.05, *p <* 0.01, and *p <* 0.001, respectively

Module	PCC with osmotic potential	PCC with H_2_O_2_ content	No. of genes	No. of upregulated genes	No. of downregulated genes
Yellow	−0.046	− 0.210***	243	133	110
Turquoise	−0.131*	−0.272***	3300	353	2947
Tan	0.076	−0.411***	52	1	51
Purple	0.128*	0.085	160	49	111
Magenta	0.099	0.164**	179	142	37
Greenyellow	0.025	−0.053	96	96	0
Green	−0.100	− 0.163**	396	0	396
Brown	0.146	0.205***	530	530	0
Blue	0.056	−0.120	962	959	3
Total	NA	NA	5918	2263	3655

The hub genes in each module played crucial roles in regulating DT and/or acclimation responses. Twenty hub nodes were therefore recommended as good candidates for DT improvement. Most of the candidates were correlated with traits of DT and involved in at least one DT-featured biological process (Additional file 2: Table S12). Meanwhile, they had minor penalties on rice growth as they were not oppositely correlated with DTIB and biomass in the well-watered field (Additional file 2: Table S12). For drought-recovery, we suggested thirteen candidates, which were involved in DT-specific biological processes at the recovery (Additional file 2: Table S13). They belonged to cluster-4 and cluster-7 in the tolerant group (Additional file 2: Table S13). The candidates had relative higher expressions in callus based on data collected from the Rice Genome Annotation Project (http://rice.plantbiology.msu.edu/index.shtml) (Additional file 2: Table S13).

## Discussion

### Tolerant and susceptible rice genotypes have distinguished transcriptomic features

The transcriptomic studies of stress tolerance via comparison between single tolerant and single susceptible rice genotype always recorded great transcriptomic differences. In this study, the mean ratio of common DRGs shared between any two rice genotypes is about one-third, which is consistent with previous studies (33.4% in Moro versus IR64, [5]; 34.5% in N22 versus IR64, [34]; 35.1% in DD versus IR20, [4]). This means a lot of different DRGs between genotypes follow the genotype-specific pattern. To avoid positive and negative false by the single genotype, we determined DRGs from 12 rice genotypes by its presence frequencies. Interestingly, the tolerant genotypes and the susceptible genotypes could be well separated by the presence and absence of total DRGs. Meanwhile, they could be distinguished by the expressions of DRGs during drought. These results indicate the transcriptomic difference between the tolerant group and the susceptible group should contribute to DT.

### Temporal differences in biological processes between susceptible and tolerant genotypes contributions to rice drought tolerance

Plant adapts to the naturally occurred drought progressively by various stepwise morphological and physiological responses [8, 29]. Although there have been numerous genome-wide transcriptomic studies on rice drought tolerance [4, 14, 23, 25, 34, 45, 46], little is known about rice temporal transcriptomic dynamics in responses to a long-term progressive drought in field conditions.

At the gene scale, less DRGs were detected in tolerant genotypes or at early stages of drought, indicating a stable transcriptome under drought is associated with DT. Meanwhile, upregulated DRGs should play an important role in DT, as tolerant genotypes have more upregulated DRGs. This is consistent with empirical selections of upregulated DEGs as candidate genes in many previous transcriptomic studies [18, 33].

**Fig. 4:**
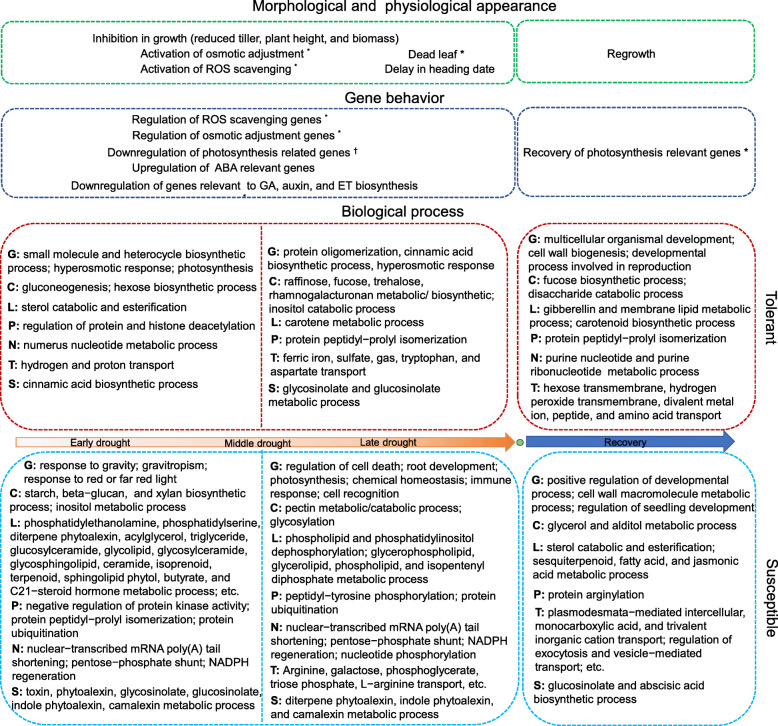
Description of drought tolerance- and susceptibility- featured morphological responses, transcriptomic dynamics, and biological processes during drought and at the recovery stage. * indicates significant difference between tolerant and susceptible genotypes; † indicates different temporal patterns between tolerant and susceptible genotypes. C: carbohydrate metabolic process; G: general biological process; L: lipid metabolic process; N: nucleic acid metabolic process; P: protein metabolic process, S: secondary metabolic process; T: transport

At the scale of biological process, the tolerant genotypes and the susceptible genotypes share many biological processes (53.4%) during the drought period (Type I), reflecting the common acclimation responses or tolerant mechanisms. Meanwhile, we also detect great temporal differences in activated biological processes between tolerant and susceptible genotypes (Type II ~ Type VI), which could help us to separate the tolerant mechanism from the passive acclimation response. Logically, these biological processes uniquely activated in tolerant genotypes (defined as Type II) shall contribute to DT. For example, many biological processes of carbohydrate metabolism (fucose, raffinose, and trehalose) are uniquely enriched in middle-latter drought by DRGs of tolerant genotypes (Fig. 4). They have been proven to be important in DT due to their roles in cell-wall synthesis, membrane protection, and photo-protection [9, 16, 28, 37]. Similar biological processes, including rhamnogalacturonan metabolic process, tryptophan and aspartate transports, etc. shall also play a role in DT at different periods (Fig. 4). Besides, if a biological process is activated earlier (e.g. hexose biosynthetic process, gluconeogenesis, etc.) and/or lasted longer (e.g. regulation of protein deacetylation, regulation of histone deacetylation, transcriptional attenuation, transcription antitermination, ferric iron import, ferric iron transport, etc.) during drought in the tolerant group, it shall be another cause of the better DT. For example, histone deacetylases play an essential role in stress responses and several genes regulating histone deacetylation (histone deacetylase and acetyltransferases) have been reported to be associated with DT [7, 19, 47]. However, roles of histone deacetylation played in rice DT have not been fully understood. In this study, we recorded three histone deacetylase genes (*LOC_Os02g12380*, *LOC_Os05g36920*, and *LOC_Os05g36930*) as DRGs. It could be valuable for testing their functions in DT. In contrast, if a biological process is initialed earlier in the susceptible group and/or then shared by the tolerant group (defined as Type V), it is very likely a drought-degree dependent process. Interestingly, protein ubiquitination, always associated with protein degradation, belongs to this type. Some other biological processes in Type III and V (e.g. regulation of cell death) reflect earlier and severer occurrences of negative impacts by drought on the susceptible genotypes. There are also many susceptibility-featured biological processes at various time points, particularly in metabolic processes of lipid and secondary metabolites (Fig. 4). Genes relevant to these biological processes could be used as molecular markers for drought susceptibility. Finally, the drought recovery also contributes to DT. It is not a simple reverse process of drought adaption as the involvement of recovery-specific DEGs and biological processes. We consider that the quick recovery in photosynthesis, growth, and development makes significant senses in drought recovery as they represent great differences between the tolerant group and the susceptible group (Fig. 4).

In a word, learning temporal transcriptomic dynamics during long-term drought can strengthen our understanding of drought tolerance and mine valuable drought-tolerant genes that refers to key biological processes. Temporal activations of genes and biological processes can further guide applications of drought-tolerant genes by temporally regulating their expressions via advanced gene engineering.

### Good balance between tolerance and productivity in drought-tolerant genotypes

Drought tolerance in crops requires relatively higher productivity under drought. However, wild plants evolve various acclimation mechanisms (e.g. stomata closure, stomata closure, leaf rolling, decrease in photosynthesis, etc.) to ensure good survival by inhibitions of growth and development [6, 11, 30]. As a result, a gene of DT may possess negative impacts (penalties) on growth and development under normal conditions, which can limit its application in breeding. The existence of penalty in growth has been reported in many previous functional studies of drought-tolerant genes, such as *OsIAA6* [15], *OsABF1* [45], and *OsCYP96B4* [35]. It means once we enhance the drought tolerance by over-expressing or inhibiting a drought-tolerant gene, it may cause unwanted yield penalty in normal conditions. We shall thus fully consider the pleiotropic effect from some drought-tolerant genes when utilizing it. In this study, we found hundreds of DRGs have contradictory correlations with productivity potential and DT, which confirms the tradeoff between DT and productivity and provides valuable cues to select propriate candidate genes in breeding.

Another valuable observation is that the regulation of a DRG does not always tend to increase drought tolerance, particularly when it has contradictory effects between DT and productivity. This phenomenon is very common as reported for many drought-tolerant genes, such as *SNAC2* [12], *OsIAA6* [15], *OsABF1* [45] etc. Interestingly, the tolerant genotypes and the susceptible genotypes are seemed to have different strategies in drought adaptation. The tolerant genotype rebuilds the balance between DT and growth much better than the susceptible genotype, by activating tolerant-specific DRGs. This leads to its stable productivity (biomass) recorded under drought. In contrast, the regulation of susceptible-specific DRGs tend to increase the biomass under normal conditions rather than enhance DT. The imbalance between DT and growth may accelerate water loss and over-reproduction of ROS, finally leading to the earlier cell death in susceptible genotypes and poorer productivity.

## Conclusion

The tolerant genotypes and the susceptible genotypes have distinguished genetic and transcriptomic bases, which underlies their different morphological and physiological responses to drought. The tolerant genotypes have their featured biological processes, which are temporally-activated through the progressive drought. Meanwhile, the tolerant and the susceptible genotypes represent some differences in their photosynthesis and cross-talks among phytohormones under drought. A considerable transcriptomic tradeoff was detected as hundreds of DRGs are oppositely correlated with DT and productivity. The tolerant genotype has a better balance between DT and productivity under drought by regulating the tolerant-specific DRGs. All these transcriptomic differences disclosed in this study contribute to rice drought tolerance. This knowledge on rice temporal transcriptomic dynamics under drought strengthens our system-level understandings in rice drought tolerance and provides us informative cues in finding and utilizing DT genes.

## Methods

### Plant materials

Twelve rice genotypes were selected to investigate their transcriptomic dynamics in responses to a long-term progressive drought, as well as their morphological and physiological performances. These plant materials were collected from International Rice Research Institute and conserved in the seed bank of Shanghai Agrobiological Gene Center (http://seed.sagc.org.cn/). The twelve rice genotypes have great differences in drought tolerance, which makes them a good system to study transcriptomic bases of drought tolerance. Meanwhile, they are important breeding materials for water-saving and drought-resistance rice (WDR) [20, 21]. Once we learn the transcriptomic features for drought tolerance in one or more genotypes, we can apply them in WDR breeding as the donors of drought tolerance.

### Field experiment design

Rice seedlings of twelve genotypes were grown in the modified field in the drought-resistance facility at Baihe Experimental Station (Shanghai, China) in 2014. The canopy can be closed on rainy days to keep rainfall out of the experimental field and to enable continuous drought. Different rice genotypes may have varied root lengths, making their capacities of accessing water at depth different. To avoid any influences caused by drought-avoidance, we imitated the design of experiments in column limited pots, making the depth of the soil layer in our experimental field only 30 cm. With this design, the developments of roots to the depth were neutralized among these rice genotypes and therefore their differences of capacities to access water at depth could be largely mitigated. Plants were grown in a plot of 10 rows× 10 hills. Three replicates for each plot were designed in both drought-treated (D) and well-watered conditions (W). The interval between hills was 18 cm. The field arrangement was followed with the single factor randomized block design. We made a minor adjustment in their dates of germination and transplanting to ensure they can meet the drought-stress before heading (Additional file 2: Table S6). We started the drought treatment on 16th, July and continued the artificial drought for 38 days. The drought-stressed field was re-watered on the afternoon of 22th, August. We measured the soil water content once every 3–5 days in the drought-stressed field to monitor and categorize the degree of drought stress at the depth of 30 cm at twelve sites distributing evenly. By monitoring the soil-water content, we can determine the time points of sampling. The averaged coefficient of variance (C.V.) of soil water content was 6.96%, reflecting homogenous levels of soil-water content across our field (Additional file 1: Figure S1).

### Measurements and analyses of morphological and physiological traits

The osmotic potential was measured to reflect the capacity of osmotic adjustment for each rice genotype via the Vapro™ vapor pressure osmometer (Wescor Model 5600). We measured the content of H_2_O_2_ using the assay kits (product#A064, Nanjing Jiancheng Bioengineering Institute, Jiangsu, China) to reflect the redox status of a rice genotype during the drought. They were measured from leaf samples of three replicates (plots). Plant height and number of tillers were measured from five individuals in each plot. These physiological and morphological traits were measured at six time points at different drought stage indicted by the soil-water content and/or developmental stages by the observation (Additional file 1: Figure S1): 24th of July (D1, later tillering), 29th of July (D2, booting), 5th of August (D3, booting), 11th of August (D4, early flowering), 22nd of August (D5, later flowering), and 23rd of August (recovery stage, R). Meanwhile, three leaf samples for each rice genotype were mixed harvested from each plot at these six time points. They were immediately put in liquid nitrogen for RNA-sequencing. The proportion of dead leaves was observed and estimated on the 23rd, August from five individual plants per plot. We measured the seed-set rating, biomass, and grain yield for each rice genotype after the harvest from all three plots with eight plants per plot. As the seed production of a rice genotype under drought was greatly influenced by its heading date, the relative biomass is more suitable for estimating its drought-tolerance. The drought-tolerant index based on biomass (DTIB) or yield (DTIY) for each rice genotype was calculated as: (biomass or yield in D/ that in W) * (biomass or yield in D/ averaged biomass or yield of all genotypes in D). Twelve genotypes could be divided into a tolerant group and a susceptible group by their DTIB. We applied the independent *t*-test or one-way ANOVA (SNK method) to detect any significant differences in the measured morphological and physiological traits between the tolerant group and the susceptible group in well-watered and drought-treated fields at each time point.

### Procedures of RNA-sequencing

Leaf samples of rice genotypes S3, S11, S17, S26, and S28 from three replicates were mixed for RNA extraction and sequencing. Other rice genotypes (S6, S9, S12, S14, S18, S24, and S31) had two or three biological replicates for RNA-sequencing as leaf samples from two or three replicates were sent for RNA extraction independently. We extracted the total RNA using the PureLink® Plant RNA Reagent (Life Technologies). We used the qualified RNA samples for library construction following the specifications of the TruSeq® RNA Sample Preparation v2 Guide (Illumina) and conducted the RNA-sequencing with Illumina Hiseq 2500 in Shanghai Majorbio Biopharm Technology Co., Ltd. (Shanghai, China). We used SeqPrep to strip adaptors and/or merging paired reads with overlap into single reads (https://github.com/jstjohn/SeqPrep) and used Sickle to remove low-quality reads (https://github.com/najoshi/sickle). We then assembled the clean data using the software Cufflinks and mapped them to the reference genome (Nipponbare, msu7.0) with mitochondrial and chloroplast genomes (http://rice.plantbiology.msu.edu/) via Tophat with no more than two base mismatches allowed in the alignment [36]. The general information of the RNA-sequencing data was provided in Table S1. The raw data for each sample had been submitted to the NCBI Sequence Read Archive (SRA) under the genotype number PRJNA609211.

### Data analysis

#### Determination of the differentially expressed gene (DEG) for each rice genotype between well-watered (W) and drought-stressed (D) treatments

We determined the gene expression levels with the Fragment Per Kilobase of exon per Million fragments mapped (FPKM) method via the widely applied software Cuffdiff [36]. The quantification of gene expression by RNA-sequencing was well validated in our previous study using same samples of two rice genotypes [23]. Since rice genotypes S6, S9, S12, S14, S18, S24, and S31 had biological replicates, we determined their DEGs via a false discovery rate (FDR) < 0.05 and a logarithm two-fold change |log2FC| ≥ 1. Given the mixed nature of the cDNA library of S3, S11, S17, S26, and S28, we thus determined its DEGs with a *p*-value < 0.05 and |log2FC| ≥ 1. Additionally, rice plant commonly delayed its development when it exposed to drought (Additional file 2: Table S15). Therefore, a part of differences in terms of transcripts between samples from the well-watered and drought-stressed conditions should be a result of delayed development. Development-dependent genes, which had differences between any two adjacent time points under the well-watered condition, were excluded from the final database for each rice genotype. The DEG detected during drought period was defined as the drought-responsive gene (DRG), while the DEG detected at recovery was defined as the recovery related gene (RRG).

A cluster analysis was conducted to determine relationships among twelve rice genotypes based on their activated DEGs (if a gene was determined as a DEG in a rice genotype, it was scored as “1”; if not, it was scored as “0”). Moreover, as DEGs with low frequencies might be occasionally detected and had low probabilities to be associated with drought-tolerance, we thus exclude DEGs determined only once (~ 44.5%) among 12 rice genotypes at a time point. Cluster analyses were conducted to see whether rice genotypes within a group (tolerant or susceptible) had common transcriptomic features, using FPKMs of total detected genes and DEGs at six time points.

DEGs having differences between the tolerant group and the susceptible group should contribute to drought-tolerance. We thus defined the group-specific DEG which was detected only in the tolerant or susceptible group. We also defined the group-different DEG, which possessed significant difference in the fold change between the tolerant group and the susceptible group by independent *t* test at a time point. Analyses of Gene Ontology (GO) enrichment was applied for group-specific and group-different DEGs by. Among three categories of GO terms (cell component, molecular function, and biological process), we paid attentions to GO terms of biological processes (GOBP). We conducted analyses of GO enrichment using the software GOATOOLS (https://github.com/tanghaibao/GOatools) [17].

#### Temporal gene regulations and relevant biological processes in the tolerant group and the susceptible group

Time-series analyses on gene regulations via hierarchical clustering (Euclidean method) were conducted in the tolerant group and the susceptible group, respectively. The drought period (from D1 to D5) and recovery process (W5-D5-R) were separately analyzed. When conducting time-series analyses during drought period, mean fold changes of DEGs within a group (tolerant or susceptible group) were used. Additionally, correlation analysis of time-series regulation for each DEG was conducted between tolerant or susceptible groups. When conducting time-series analyses at recovery stage, eight regulation modes (clusters) were defined by regulations (up-regulation, unchanged, or down-regulation) from “W5 to D5” and “D5 to R”. Analyses of GO enrichment (FDR < 0.05) were conducted for each mode of the tolerant group and the susceptible group during the recovery process, respectively. PCoA was also conducted for each mode based on its enriched GOBPs.

To describe temporal biological processes in responses to the long-term drought, analyses of GO enrichment (FDR < 0.05) were conducted based on DEGs at each time points in the tolerant group and the susceptible group, respectively. PCoA was conducted to describe similarities of transcriptome features between the tolerant group and the susceptible group at six time points using their corresponding DEGs and enriched GOBPs. The enriched GOBPs were further functionally categorized in to dozens of classifications by a web tools named “GO Terms Classifications Counter” (http://www.animalgenome.org/cgi-bin/util/gotreei) [13]. We applied correspondence analysis to investigate differences of GO classifications between the tolerant group and the susceptible group in responses to the drought at various time points.

We further investigated differences of GOBPs between the tolerant group and the susceptible group in six important classifications of biological processes (*p* < 0.05 as confidence interval): carbohydrate metabolic process (GO:0005975), protein metabolic process (GO:0006464), lipid metabolic process (GO:0006629), secondary metabolic process (GO:0019748), nucleic acid metabolic process (GO:0006139, short for nucleobase, nucleoside, nucleotide and nucleic acid metabolic process), and transport (GO:0006810). Based on temporal patterns of GO enrichments during drought (D1 to D5), terms of GOBP could divided in to six modes: (I) having the same temporal pattern between the tolerant group and the susceptible group; (II) DT-specific (uniquely enriched in tolerant group); (III) susceptibility-specific (uniquely enriched in susceptible group); (IV) initiated earlier and/or lasted longer in the tolerant group; (V) initiated earlier and/or lasted longer in the susceptible group; (VI) having other different temporal patterns.

As regulation of photosynthesis is a primary acclimation response to drought stress in plant [11] and has been reported to be associated with drought-tolerance in crops [23, 46], we investigated temporal expressions of photosynthesis relevant DEGs (genes annotated to GO:0009767, GO:0009772, GO:0015979, and GO:0022900) in the tolerant group and the susceptible group. Cluster analyses were conducted to see whether rice genotypes within a group (tolerant or susceptible) had common transcriptomic features in terms of photosynthesis, using their FPKMs.

#### Correlation analysis between DRGs annotated to eight phytohormones

As the cross-talk among phytohormones plays a crucial role in plant drought adaptation and tolerance [38], we conducted correlation analyses (Pearson’s correlation coefficient, PCC) among DRGs annotated to KEGG pathways of the eight phytohormones (ABA (map00906), GA (map00904), auxin (map00380 and map00400), ET (map00270 and map00625), SA (map00360, map00624, and map00626), JA (map00592), KT (map00908), and BR(map00905)). The potential cross-talk between two phytohormones were determined by the mean PCC value among DRGs annotated to the two phytohormones.

#### The weighted gene co-expression network analysis (WGCNA)

Co-expression gene network modules (highly co-expressed clusters of genes) were inferred by WGCNA. The automatic one-step network construction and module detection method with default settings were used, which include an unsigned type of topological overlap matrix (TOM). To gain biologically significant modules in a weighted gene co-expression network, the cutoff threshold of edges was determined by the method described previously, at where the network density displays a minimal value [1]. Edges above the cutoff indicated significant relationships of co-expression and were thus determined as significant edges. Only nodes with significant edges were retained in the gene co-expression network. Their values of module eigengenes were calculated and used to test their associations with morphological, physiological, and agronomic traits by correlation analyses. A gene with the top 15% connectivity in a module was determined as a hub gene. Analyses of GO enrichment (FDR < 0.05) were also conducted for each module to investigate its potential biological functions.

#### Correlation analyses of between fold changes/expressions of DRGs and drought-tolerance/agronomic traits

We conducted a correlation analysis between averaged fold changes (mean fold changes from D_1_ to D_5_) of a DRG and drought-tolerances (DTIB) among twelve rice genotypes (|PCC| > 0.576 and *p* value < 0.05 for significance). As biomass in the well-watered field represented the growth and productivity, we also conducted correlation analysis between the biomass and averaged gene expression (mean of FPKMs from W1 to W5) in the well-watered field (|PCC| > 0.576 and *p* value < 0.05 for significance) among twelve rice genotypes. Contradictories of coefficients (positively correlated with DTIB but negatively correlated with biomass; or reverse) from two correlation analyses above could reflect tradeoffs between drought-tolerance and growth at the scale of gene expression. We also conducted correlation analyses between gene expression levels and osmotic potential /H_2_O_2_ content using all samples during drought.

## Supplementary Information

**Additional file 1: Supplementary Figure S1.** Soil water contents monitored during the experimental period. The grey dot and dashed line indicate coefficient of variance (C.V.). **Supplementary Figure S2.** Venn diagram of DEGs detected during drought period, DEGs detected at recovery, and function-studied drought-tolerant (DT) genes. **Supplementary Figure S3.** Overlap of DEGs detected among genotypes or between the tolerant and the susceptible groups. **Supplementary Figure S4.** Mean absolute Log_2_(fold changes) of DEGs of different frequencies detected at among 12 rice genotypes from time points D1 to R (a-f). **Supplementary Figure S5.** Cluster analysis of 12 rice genotypes in drought (D) and well-watered (W) fields based on expressions levels of total expressed genes from time points D1 to R (a-f). **Supplementary Figure S6.** Cluster analysis of 12 rice genotypes in drought (D) and well-watered (W) fields based on expressions levels of DEGs from time points D1 to R (a-f). **Supplementary Figure S7.** Cluster analysis of 12 rice genotypes by SNP called from total transcripts (a) and transcripts of DEGs (b). **Supplementary Figure S8.** Results of principal component analysis of the tolerant and the susceptible groups at six time points (D1-D5 and R) based on their DEGs (a) and enriched GO biological processes (b). **Supplementary Figure S9.** Venn diagram of drought-responsive genes (DRG) and recovery related genes (RRG) in the tolerant and the susceptible groups. **Supplementary Figure S10.** Distributions of enriched GO biological processes in various GO classifications by tolerant (T)-specific, susceptible (S)-specific, and T-S different DRGs. **Supplementary Figure S11.** Time-series clusters based on Log_2_(fold change) in the tolerant groups during drought period. **Supplementary Figure S12.** Time-series clusters based on Log_2_(fold change) in the susceptible groups during drought period. **Supplementary Figure S13.** Regulation modes of recovery related genes (RRGs) and their differences between the tolerant and the susceptible groups. **Supplementary Figure S14.** A heatmap describing temporal differences of enriched GO biological processes (Bonferroni corrected *p* < 0.05) between the tolerant and the susceptible groups at six time points (D1-D5 and R). **Supplementary Figure S15.** A heatmap describing temporal differences of enriched GO biological processes (*p <* 0.05) in the GO classification of carbohydrate metabolic process (GO:0005975) between the tolerant and the susceptible groups at six time points (D1-D5 and R). **Supplementary Figure S16.** A heatmap describing temporal differences of enriched GO biological processes (*p <* 0.05) in the GO classification of lipid metabolic process (GO:0006629) between the tolerant and the susceptible groups at six time points (D1-D5 and R). **Supplementary Figure S17.** A heatmap describing temporal differences of enriched GO biological processes (*p <* 0.05) in the GO classification of protein metabolic process (GO:0019538) between the tolerant and the susceptible groups at six time points (D1-D5 and R). **Supplementary Figure S18.** A heatmap describing temporal differences of enriched GO biological processes (*p <* 0.05) in the GO classification of nucleobase, nucleoside, nucleotide, and nucleic acid metabolic process (GO:0006139) between the tolerant and the susceptible groups at six time points (D1-D5 and R). **Supplementary Figure S19.** A heatmap describing temporal differences of enriched GO biological processes (*p <* 0.05) in the GO classification of transporter (GO:0006810) between the tolerant and the susceptible groups at six time points (D1-D5 and R). **Supplementary Figure S20.** A heatmap describing temporal differences of enriched GO biological processes (*p <* 0.05) in the GO classification of secondary metabolic process (GO:0019748) between the tolerant and the susceptible groups at six time points (D1-D5 and R). **Supplementary Figure S21.** Cluster analyses of rice genotypes in the drought field (D) at time points D1 to D5 (a-e) and recovery stage (R) (f) based on expressions of photosynthesis-relevant genes. **Supplementary Figure S22.** Cluster analyses of rice genotypes in the well-watered field (W) at time points W1 to W5 (a-e) based on expressions of photosynthesis-relevant genes. **Supplementary Figure S23.** A heatmap of regulations of photosynthesis-relevant DRGs during drought period (D1-D5) and at recovery (R) by mean log_2_(fold changes) in tolerant (T) and susceptible (S) groups. **Supplementary Figure S24.** A heatmap of regulations of phytohormone-relevant DRGs during drought period (D1-D5) and at recovery (R) by mean log_2_(fold changes) in tolerant (T) and susceptible (S) groups. **Supplementary Figure S25.** The matrix of mean Pearson’s correlation coefficients (PCCs) calculated from DRGs among eight phytohormones. The above matrix is of tolerant genotypes while the below one is of susceptible genotypes. **Supplementary Figure S26.** Venn diagram of (a) osmolality- and H_2_O_2_- correlated drought-responsive genes and (b) DTIB- and biomass- correlated drought-responsive genes. **Supplementary Figure S27.** Comparisons of absolute values of Log_2_(Fold change) of osmolality- (a) and H_2_O_2_-correlated (b) drought-responsive genes (DRGs) between the tolerant and the susceptible groups at six time points. Bars indicate standard errors. “*”, “**”, and “***” indicate significant differences at *p <* 0.05, *p* < 0.01, and *p* < 0.001 by independent *t* test between the tolerant and the susceptible groups.

**Additional file 2: Supplementary Table S1.** Basic information of RNA-seq for all samples. **Supplementary Table S2.** Total expressed genes (FPKM> 0.001) detected in twelve rice genotypes. **Supplementary Table S3.** Function-studied drought-tolerant genes, their correlation with drought-tolerance index by biomass (DTIB) and biomass measured in well-watered fields. **Supplementary Table S4.** Number of enriched GOBPs by upregulated and downregulated DRGs in various classifications of biological processes. Number in shade indicates the GO classification preferred by upregulated or downregulated DRGs. **Supplementary Table S5.** Correlations of fold changes from common drought-responsive genes between the tolerant and the susceptible groups during drought. **Supplementary Table S6.** Number of biological processes in various GO classifications enriched by recovery related genes in different clusters. **Supplementary Table S7.** Distributions of GO biological processes of different types in various GO classifications. Percentage in shade indicates the GO classification preferred by the type of GO biological processes. **Supplementary Table S8.** Enrichments of GO biological processes by osmolality- and H2O2-correlated drought responsive genes. **Supplementary Table S9.** Distribution of biological processes enriched by osmolality- and H2O2-correlated drought responsive genes in various GO classification. **Supplementary Table S10.** The enrichment of DT-featured biological processes in Gene Ontology for nine modules. **Supplementary Table S11.** Distribution of genes relevant to photosynthesis and phytohormones in the modules. **Supplementary Table S12.** Information of candidate genes of drought-tolerance. **Supplementary Table S13.** Information of candidate genes for drought-recovery.

## Data Availability

All data supporting the conclusions of this article are provided within the article and its supplementary files. The raw sequence data included in this study has been deposited into the NCBI Sequence Read Archive (SRA) under the accession number of PRJNA609211.

## Abbreviations

ABA: Abscisic acid
BP: Biological process
BR: Brassinosteroids
DEG: Differentially expressed genes
DRG: Drought-responsive genes
DT: Drought tolerance
DTIB: Drought tolerance index based on biomass
DTIY: Drought-tolerant index based on yield
ET: Ethylene
FDR: False discovery rate
FPKM: Fragment per kilobase of exon per million fragments mapped
GA: Gibberellin
GO: Gene ontology
JA: Jasmonic acid
KT: Cytokinin
PCC: Pearson’s correlation coefficient
PCoA: Principal component analysis
RRG: Recovery related genes
SA: Salicylic acid
WDR: Water-saving and drought-resistance rice
WGCNA: Weighted gene coexpression network analysis
