# Supplementary material for: Temporal transcriptomic differences between tolerant and susceptible genotypes contribute to rice drought tolerance
Source: BMC Genomics. 2020 Nov 10;21:776. doi: 10.1186/s12864-020-07193-7 (PMC7654621; doi:10.1186/s12864-020-07193-7)
Supplement: Supplementary file 1 — Additional file 1: Supplementary Figure S1. Soil water contents monitored during the experimental period. The grey dot and dashed line indicate coefficient of variance (C.V.). Supplementary Figure S2. Venn diagram of DEGs detected during drought period, DEGs detected at recovery, and function-studied drought-tolerant (DT) genes. Supplementary Figure S3. Overlap of DEGs detected among genotypes or between the tolerant and the susceptible groups. Supplementary Figure S4. Mean absolute Log2(fold changes) of DEGs of different frequencies detected at among 12 rice genotypes from time points D1 to R (a-f). Supplementary Figure S5. Cluster analysis of 12 rice genotypes in drought (D) and well-watered (W) fields based on expressions levels of total expressed genes from time points D1 to R (a-f). Supplementary Figure S6. Cluster analysis of 12 rice genotypes in drought (D) and well-watered (W) fields based on expressions levels of DEGs from time points D1 to R (a-f). Supplementary Figure S7. Cluster analysis of 12 rice genotypes by SNP called from total transcripts (a) and transcripts of DEGs (b). Supplementary Figure S8. Results of principal component analysis of the tolerant and the susceptible groups at six time points (D1-D5 and R) based on their DEGs (a) and enriched GO biological processes (b). Supplementary Figure S9. Venn diagram of drought-responsive genes (DRG) and recovery related genes (RRG) in the tolerant and the susceptible groups. Supplementary Figure S10. Distributions of enriched GO biological processes in various GO classifications by tolerant (T)-specific, susceptible (S)-specific, and T-S different DRGs. Supplementary Figure S11. Time-series clusters based on Log2(fold change) in the tolerant groups during drought period. Supplementary Figure S12. Time-series clusters based on Log2(fold change) in the susceptible groups during drought period. Supplementary Figure S13. Regulation modes of recovery related genes (RRGs) and their differences between the tol [file 12864_2020_7193_MOESM1_ESM.pdf]

Supplementary Figure Legends

Figure S1. Soil water contents monitored during the experimental period. The grey dot and dashed line indicate coefficient of variance (C.V.). This figure can also be found in a previous study (Ma et al. 2016) as the two experiments were conducted together.

Figure S2. Venn diagram of DEGs detected during drought period, DEGs detected at recovery, and function-studied drought-tolerant (DT) genes. (a) All detected DEGs. (b) DEGs detected at least in two genotypes (frequency≥2).

Figure S3. Overlap of DEGs detected among genotypes or between tolerant and susceptible groups. (a) Matrix of ratio of shared DEGs between any two rice genotypes. The value in diagonal indicates number of DEGs detected in the genotype during drought-period. The result of cluster analysis on the right is based on the involvement of DEGs detected among 12 rice genotypes. (b) Venn diagram of DEGs (frequency≥2) detected in tolerant and susceptible groups at six time points.

Figure S4. Mean absolute Log_2(fold changes) of DEGs of different frequencies detected at among 12 rice genotypes from time points D1 to R (a-f).

Figure S5. Cluster analysis of 12 rice genotypes in drought (D) and well-watered (W) fields based on expressions levels of total expressed genes from time points D1 to R (a-f).

Figure S6. Cluster analysis of 12 rice genotypes in drought (D) and well-watered (W) fields based on expressions levels of DEGs from time points D1 to R (a-f).

Figure S7. Cluster analysis of 12 rice genotypes by SNP called from total transcripts (a) and transcripts of DEGs (b).

Figure S8. Results of principal component analysis of tolerant and susceptible groups at six time points (D1-D5 and R) based on their DEGs (a) and enriched GO biological processes (b).

Figure S9. Venn diagram of drought-responsive genes (DRG) and recovery related genes (RRG) in tolerant and susceptible groups.

Figure S10. Distributions of enriched GO biological processes in various GO classifications by tolerant (T)-specific, susceptible (S)-specific, and S-T different DRGs.

Figure S11. Time-series clusters based on Log_2(fold change) in the tolerant groups during drought period. Fold change is calculated as: FPKM in drought (FPKM-D)/FPKM in well-watered (FPKM-W). The number on top indicates number of genes in this cluster. Numbers at bottom indicate number of negatively osmolality-correlated, positively osmolality-correlated, negatively H_2O_2-correlated, positively H_2O_2-correlated genes in this cluster.

Figure S12. Time-series clusters based on Log_2(fold change) in the susceptible groups during drought period. Fold change is calculated as: FPKM in drought (FPKM-D)/FPKM in well-watered (FPKM-W). The number on top indicates number of genes in this cluster. Numbers at bottom indicate number of negatively osmolality-correlated, positively osmolality-correlated, negatively H_2O_2-correlated, positively H_2O_2-correlated genes in this cluster.

Figure S13. Regulation modes of recovery related genes (RRGs) and their differences between tolerant and susceptible groups. (a) Eight regulation modes of RRGs among well-watered samples at 5th time point (W5), drought-treated samples at 5th time point (D5), and after recovery (R). (b) The circle size of a mode indicates the number of RRGs in it. The width of a line between two modes indicates the number of common

RRGs shared by them. (c) Result of principal component analysis of different modes based on their enriched GO biological process.

Figure S14. A heatmap describing temporal differences of enriched GO biological processes (Bonferroni corrected p<0.05) between tolerant and susceptible groups at six time points (D1-D5 and R). Some typical tolerant- and susceptible-featured biological processes are emphasized in red and green colors, respectively. “*” indicates recovery-specific biological processes.

Figure S15. A heatmap describing temporal differences of enriched GO biological processes (p<0.05) in the GO classification of carbohydrate metabolic process (GO:0005975) between tolerant and susceptible groups at six time points (D1-D5 and R). Some typical tolerant-featured biological processes are emphasized in red.

Figure S16. A heatmap describing temporal differences of enriched GO biological processes (p<0.05) in the GO classification of lipid metabolic process (GO:0006629) between tolerant and susceptible groups at six time points (D1-D5 and R). Some typical tolerant-featured biological processes are emphasized in red.

Figure S17. A heatmap describing temporal differences of enriched GO biological processes (p<0.05) in the GO classification of protein metabolic process (GO:0019538) between tolerant and susceptible groups at six time points (D1-D5 and R). Some typical tolerant-featured biological processes are emphasized in red.

Figure S18. A heatmap describing temporal differences of enriched GO biological processes (p<0.05) in the GO classification of nucleobase, nucleoside, nucleotide and nucleic acid metabolic process (GO:0006139) between tolerant and susceptible groups at six time points (D1-D5 and R).

Figure S19. A heatmap describing temporal differences of enriched GO biological

processes ($p<0.05$) in the GO classification of transporter (GO:0006810) between tolerant and susceptible groups at six time points (D1-D5 and R). Some typical tolerant-featured biological processes are emphasized in red.

Figure S20. A heatmap describing temporal differences of enriched GO biological processes ($p<0.05$) in the GO classification of secondary metabolic process (GO:0019748) between tolerant and susceptible groups at six time points (D1-D5 and R).

Figure S21. Cluster analyses of rice genotypes in the drought field (D) at time points D1 to D5 (a-e) and recovery stage (R) (f) based on expressions of photosynthesis-relevant genes. Tolerant genotypes are indicated in red.

Figure S22. Cluster analyses of rice genotypes in the well-watered field (W) at time points W1 to W5 (a-e) based on expressions of photosynthesis-relevant genes. Tolerant genotypes are indicated in red.

Figure 23. A heatmap of regulations of photosynthesis-relevant DRGs during drought period (D1-D5) and at recovery (R) by mean log_2(fold changes) in tolerant (T) and susceptible (S) groups.

Figure 24. A heatmap of regulations of phytohormone-relevant DRGs during drought period (D1-D5) and at recovery (R) by mean log_2(fold changes) in tolerant (T) and susceptible (S) groups.

Figure S25. The matrix of mean Pearson's correlation coefficients (PCCs) calculated from DRGs among eight phytohormones. The above matrix is of tolerant genotypes while the below one is of susceptible genotypes.

Figure S26. Venn diagram of (a) osmolality- and H_2O_2- correlated drought-responsive

genes and (b) DTIB- and biomass- correlated drought-responsive genes.

Figure S27. Comparisons of absolute values of Log_2(Fold change) of osmolality- (a) and H_2O_2-correlated (b) drought-responsive genes (DRGs) between tolerant and susceptible groups at six time points. Bars indicate standard errors. “*”, “**”, and “***” indicate significant differences at $p<0.05$, $p<0.01$, and $p<0.001$ by independent *t* test between tolerant and susceptible groups.

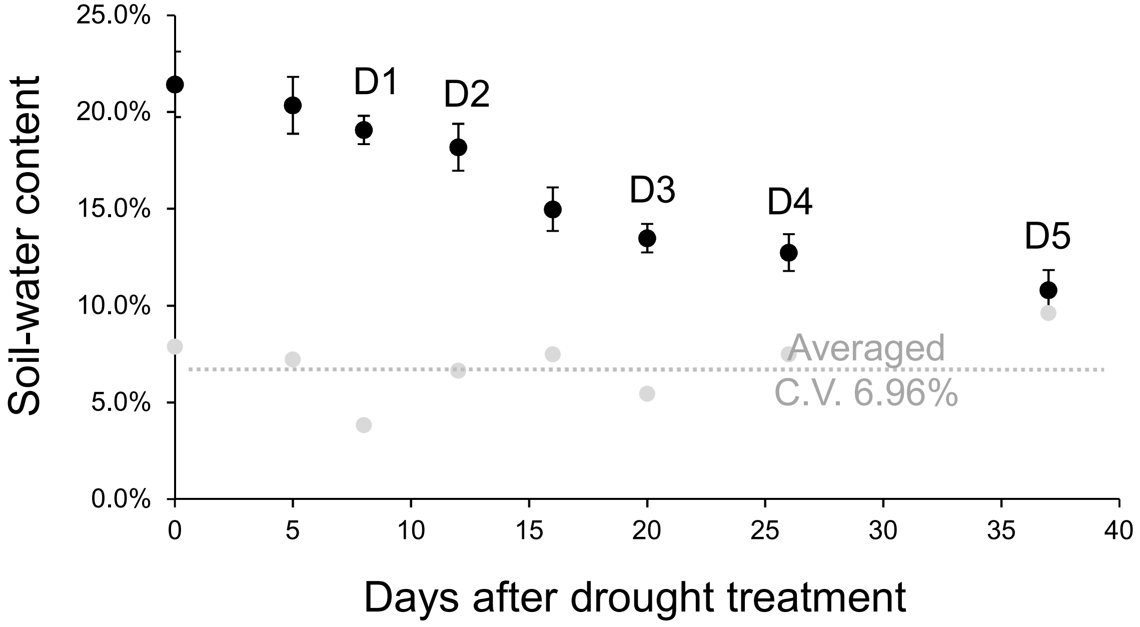
25.0%
20.0%
15.0%
10.0%
5.0%
0.0%
Soil-water content
D1
D2
D3
D4
D5
Averaged
C.V. 6.96%
0
5
10
15
20
25
30
35
40
Days after drought treatment

Fig. S1

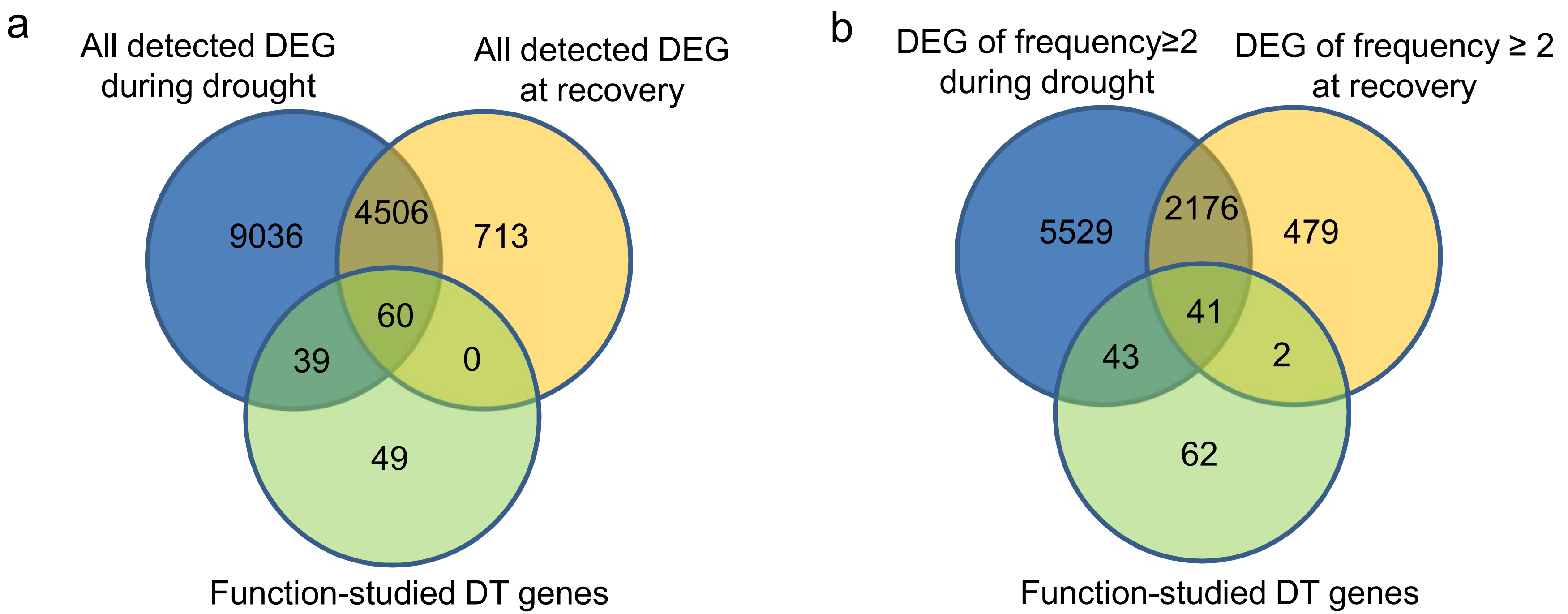
a
All detected DEG
during drought
All detected DEG
at recovery
9036
4506
713
60
39
0
49
Function-studied DT genes
b
DEG of frequency≥2
during drought
DEG of frequency ≥ 2
at recovery
5529
2176
479
41
43
2
62
Function-studied DT genes

Fig. S2

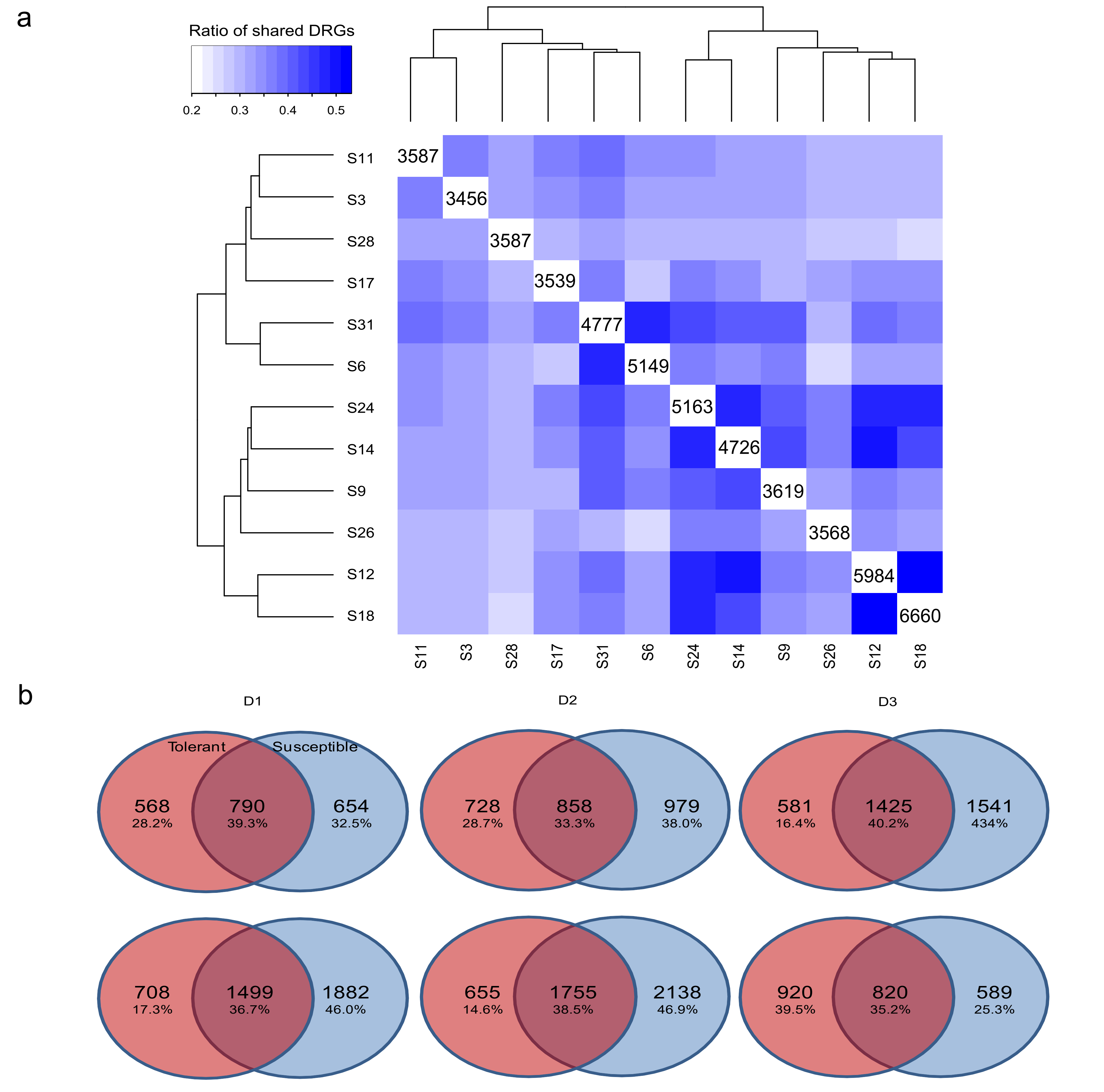
a
Ratio of shared DRGs
0.2
0.3
0.4
0.5
S11
S3
S28
S17
S31
S6
S24
S14
S9
S26
S12
S18
3587
3456
3587
3539
4777
5149
5163
4726
3619
3568
5984
6660
b
D1
Tolerant
Susceptible
568
28.2%
790
39.3%
654
32.5%
D2
728
28.7%
858
33.3%
979
38.0%
D3
581
16.4%
1425
40.2%
1541
434%
708
17.3%
1499
36.7%
1882
46.0%
D4
655
14.6%
1755
38.5%
2138
46.9%
D5
920
39.5%
820
35.2%
589
25.3%
R

Fig. S3

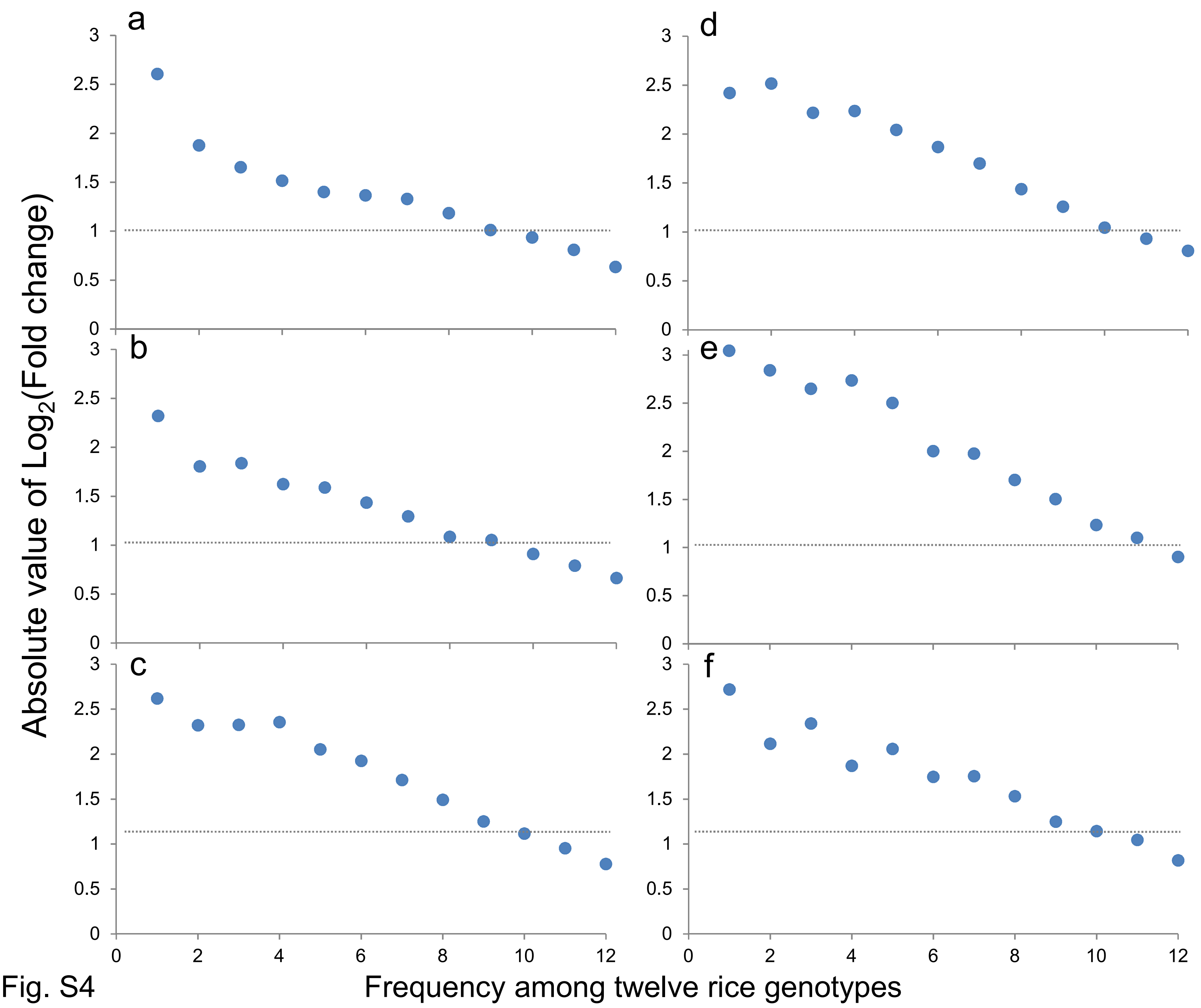

a
b
c
d
e
f
3
2.5
2
1.5
1
0.5
0
0
2
4
6
8
10
12
Absolute value of Log2(Fold change)
Frequency among twelve rice genotypes

Fig. S4

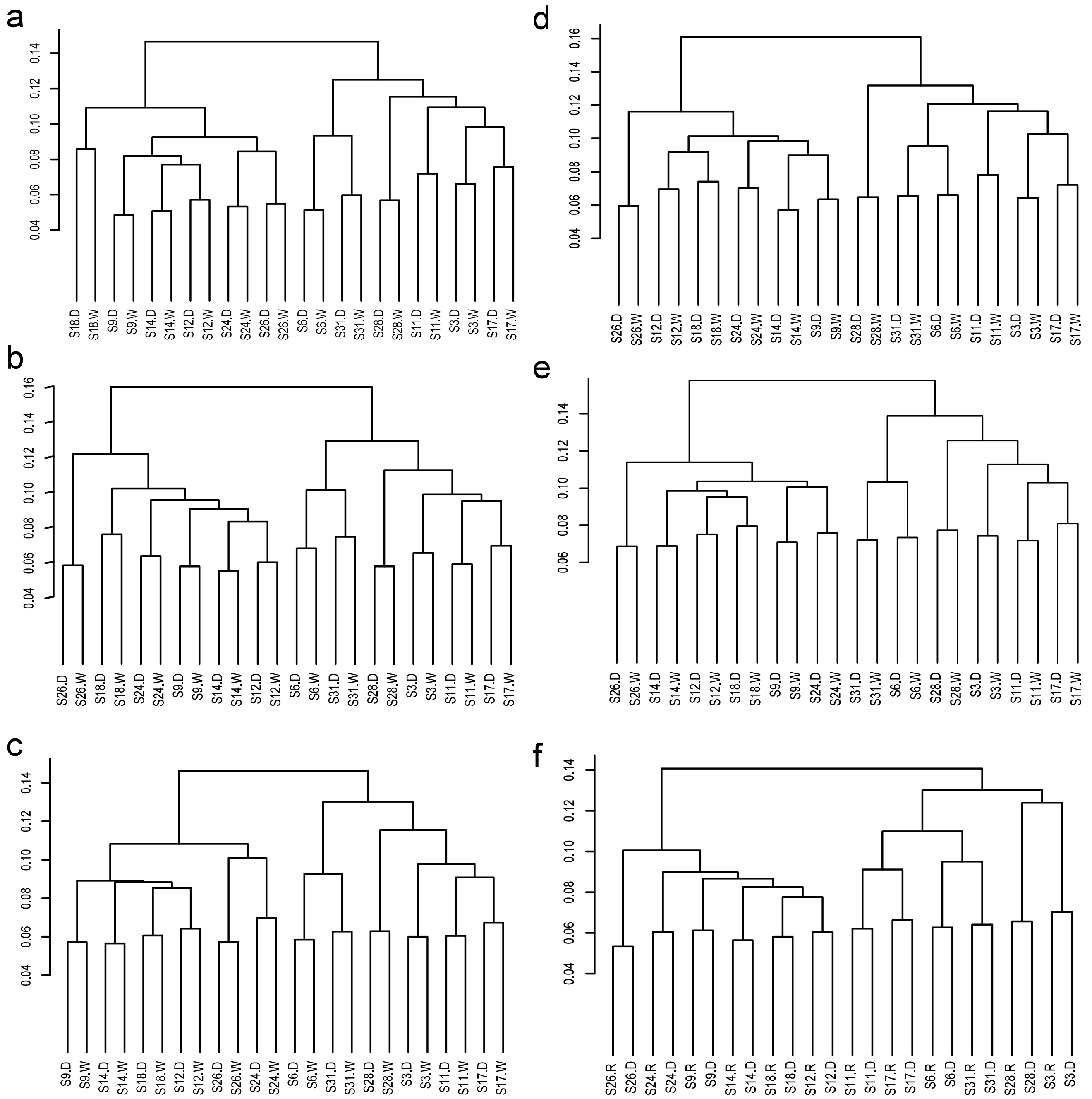
a
S18.D
S18.W
S9.D
S9.W
S14.D
S14.W
S12.D
S12.W
S24.D
S24.W
S26.D
S26.W
S6.D
S6.W
S31.D
S31.W
S28.D
S28.W
S11.D
S11.W
S3.D
S3.W
S17.D
S17.W
b
S26.D
S26.W
S18.D
S18.W
S24.D
S24.W
S9.D
S9.W
S14.D
S14.W
S12.D
S12.W
S6.D
S6.W
S31.D
S31.W
S28.D
S28.W
S3.D
S3.W
S11.D
S11.W
S17.D
S17.W
c
S9.D
S9.W
S14.D
S14.W
S18.D
S18.W
S12.D
S12.W
S26.D
S26.W
S24.D
S24.W
S6.D
S6.W
S31.D
S31.W
S28.D
S28.W
S3.D
S3.W
S11.D
S11.W
S17.D
S17.W
d
S26.D
S26.W
S12.D
S12.W
S18.D
S18.W
S24.D
S24.W
S14.D
S14.W
S9.D
S9.W
S28.D
S28.W
S31.D
S31.W
S6.D
S6.W
S11.D
S11.W
S3.D
S3.W
S17.D
S17.W
e
S26.D
S26.W
S14.D
S14.W
S12.D
S12.W
S18.D
S18.W
S9.D
S9.W
S24.D
S24.W
S31.D
S31.W
S6.D
S6.W
S28.D
S28.W
S3.D
S3.W
S11.D
S11.W
S17.D
S17.W
f
S26.R
S26.D
S24.R
S24.D
S9.R
S9.D
S14.R
S14.D
S18.R
S18.D
S12.R
S12.D
S11.R
S11.D
S17.R
S17.D
S6.R
S6.D
S31.R
S31.D
S28.R
S28.D
S3.R
S3.D

Fig. S5

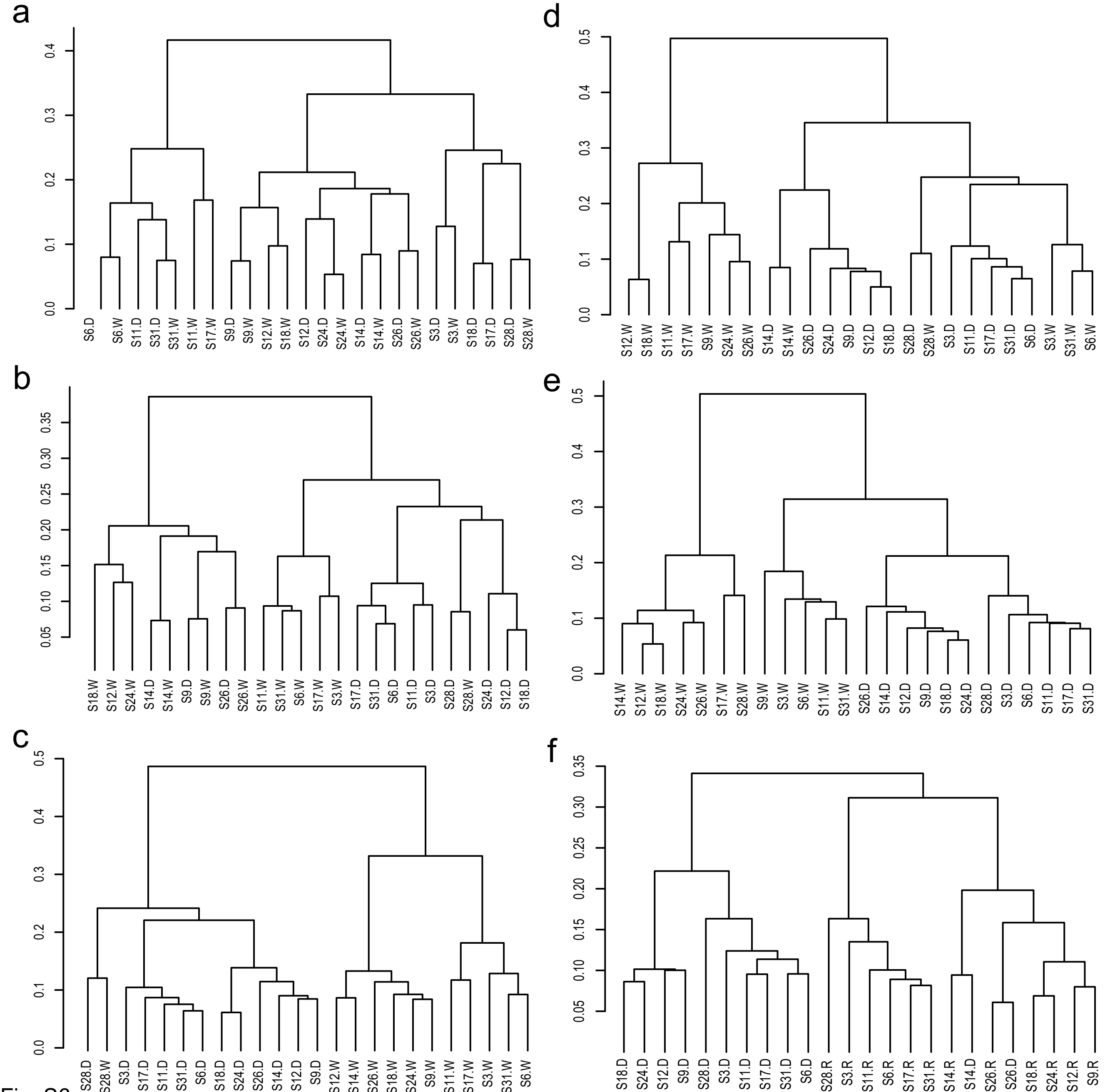
a
S6.D S6.W S11.D S31.D S31.W S11.W S17.W S9.D S9.W S12.W S18.W S12.D S24.D S24.W S14.D S14.W S26.D S26.W S3.D S3.W S18.D S17.D S28.D S28.W
b
S18.W S12.W S24.W S14.D S14.W S9.D S9.W S26.D S26.W S11.W S31.W S6.W S17.W S3.W S17.D S31.D S6.D S11.D S3.D S28.D S28.W S24.D S12.D S18.D
c
S28.D S28.W S3.D S17.D S11.D S31.D S6.D S18.D S24.D S26.D S14.D S12.D S9.D S12.W S14.W S26.W S18.W S24.W S9.W S11.W S17.W S3.W S31.W S6.W
d
S12.W S18.W S11.W S17.W S9.W S24.W S26.W S14.D S14.W S26.D S24.D S9.D S12.D S18.D S28.D S28.W S3.D S11.D S17.D S31.D S6.D S3.W S31.W S6.W
e
S14.W S12.W S18.W S24.W S26.W S17.W S28.W S9.W S3.W S6.W S11.W S31.W S26.D S14.D S12.D S9.D S18.D S24.D S28.D S3.D S6.D S11.D S17.D S31.D
f
S18.D S24.D S12.D S9.D S28.D S3.D S11.D S17.D S31.D S6.D S28.R S3.R S11.R S6.R S17.R S31.R S14.R S14.D S26.R S26.D S18.R S24.R S12.R S9.R
Fig. S6

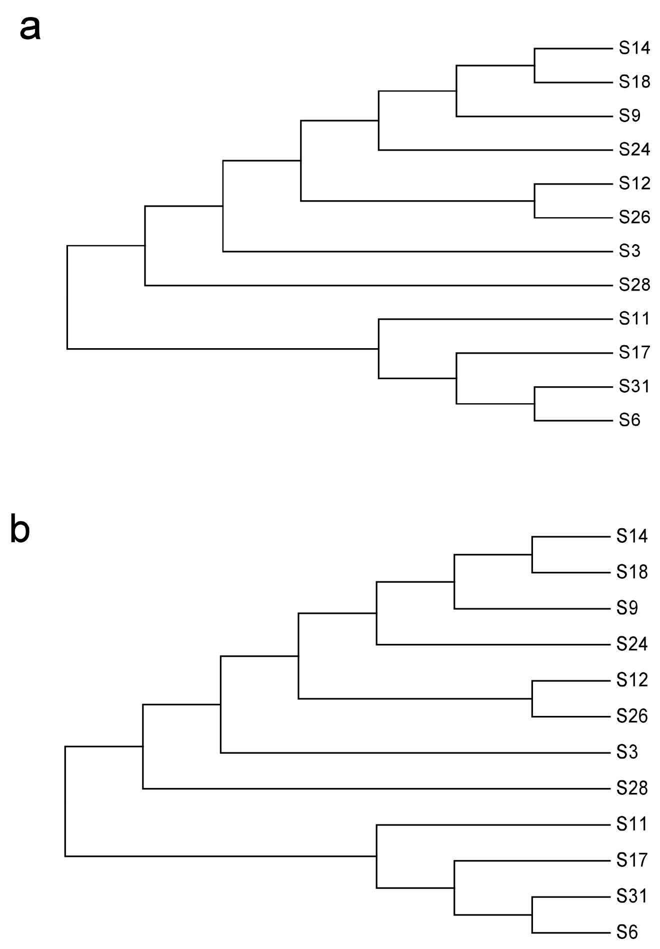
a
S14
S18
S9
S24
S12
S26
S3
S28
S11
S17
S31
S6
b
S14
S18
S9
S24
S12
S26
S3
S28
S11
S17
S31
S6

Fig. S7

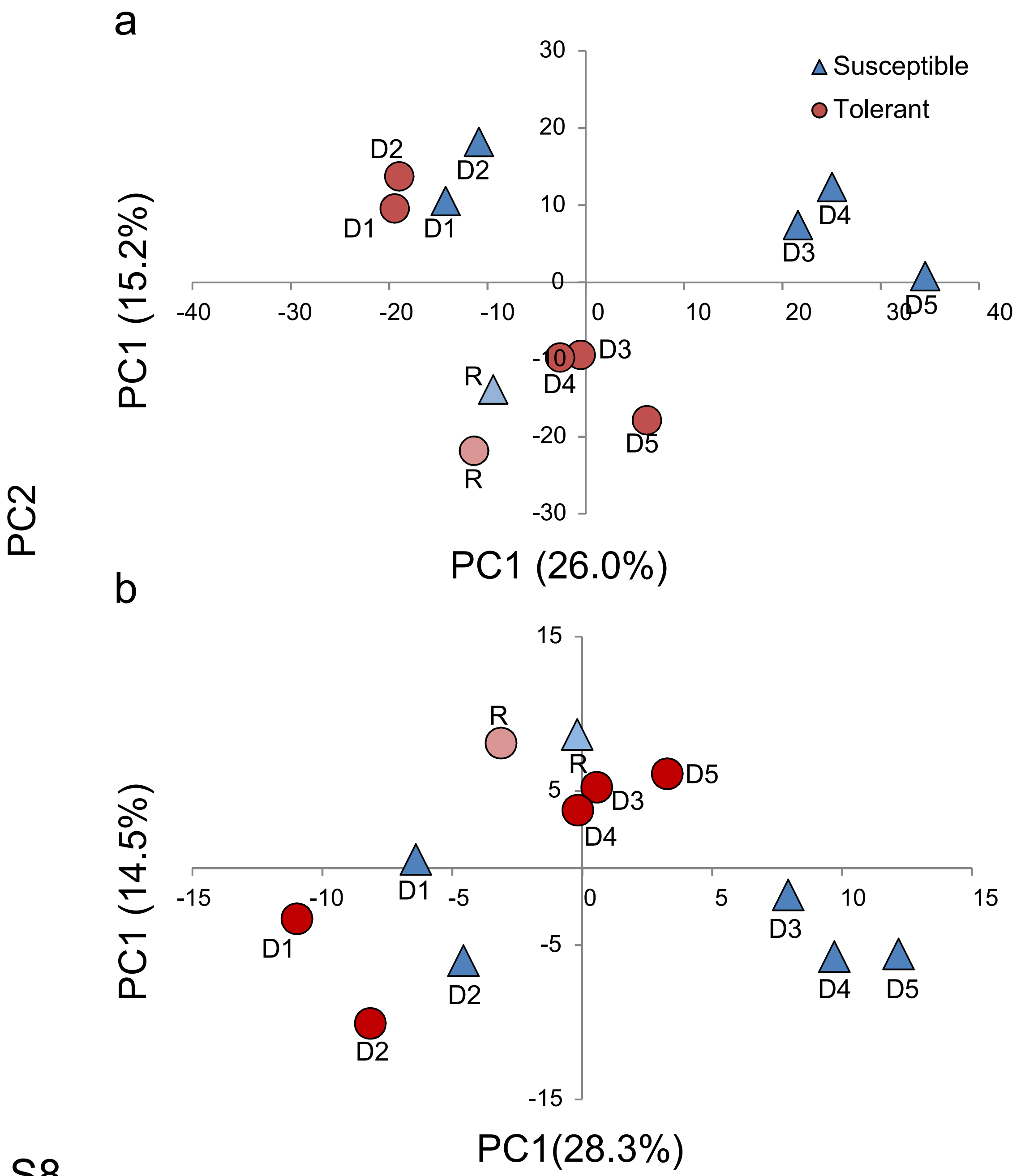

Fig. S8

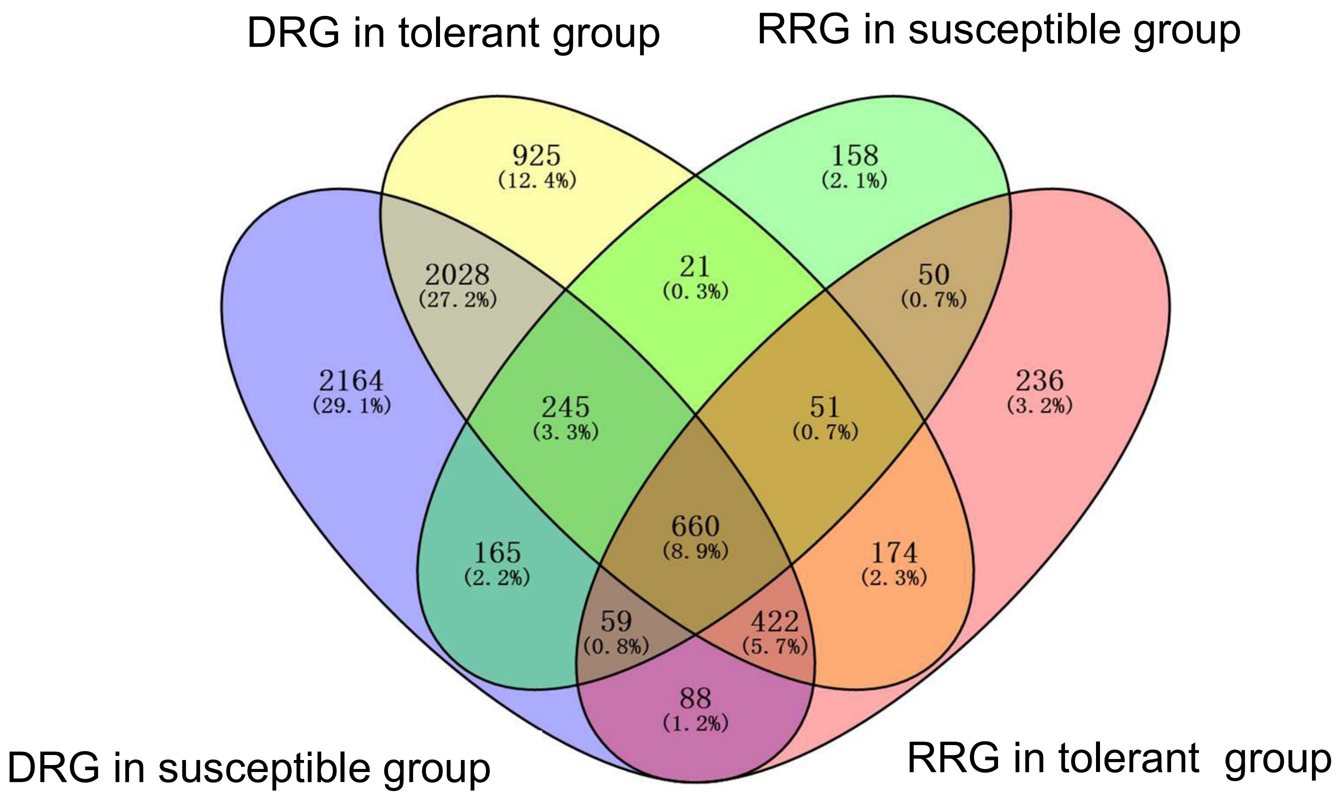
DRG in tolerant group
RRG in susceptible group
DRG in susceptible group
RRG in tolerant group
925
(12.4%)
158
(2.1%)
2028
(27.2%)
21
(0.3%)
50
(0.7%)
2164
(29.1%)
245
(3.3%)
51
(0.7%)
236
(3.2%)
660
(8.9%)
165
(2.2%)
174
(2.3%)
59
(0.8%)
422
(5.7%)
88
(1.2%)

Fig. 9

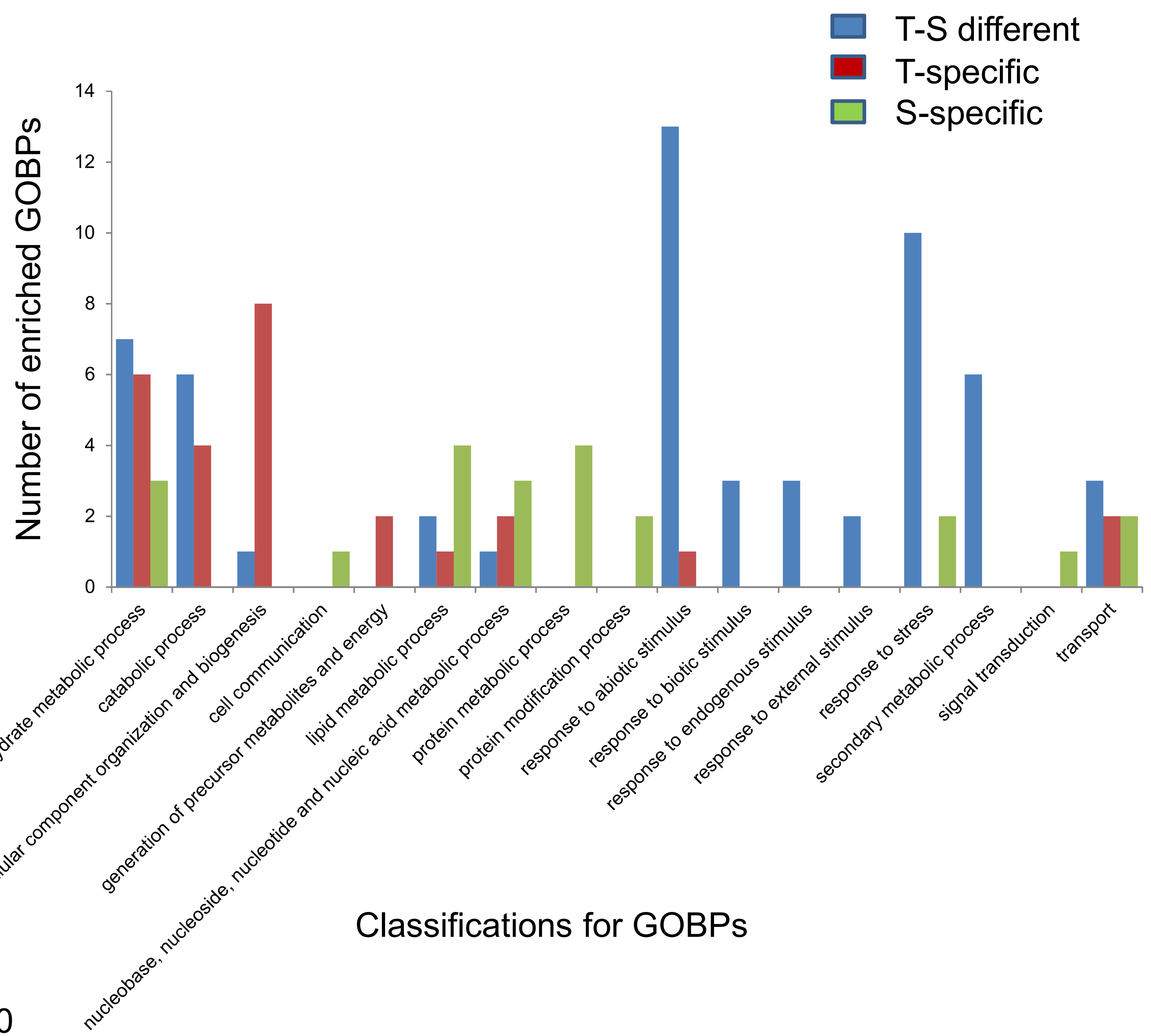
T-S different
T-specific
S-specific
Number of enriched GOBPs
14
12
10
8
6
4
2
0
carbohydrate metabolic process
catabolic process
cellular component organization and biogenesis
cell communication
generation of precursor metabolites and energy
lipid metabolic process
nucleobase, nucleoside, nucleotide and nucleic acid metabolic process
protein metabolic process
protein modification process
response to abiotic stimulus
response to biotic stimulus
response to endogenous stimulus
response to external stimulus
response to stress
secondary metabolic process
signal transduction
transport
Classifications for GOBPs

Fig. S10

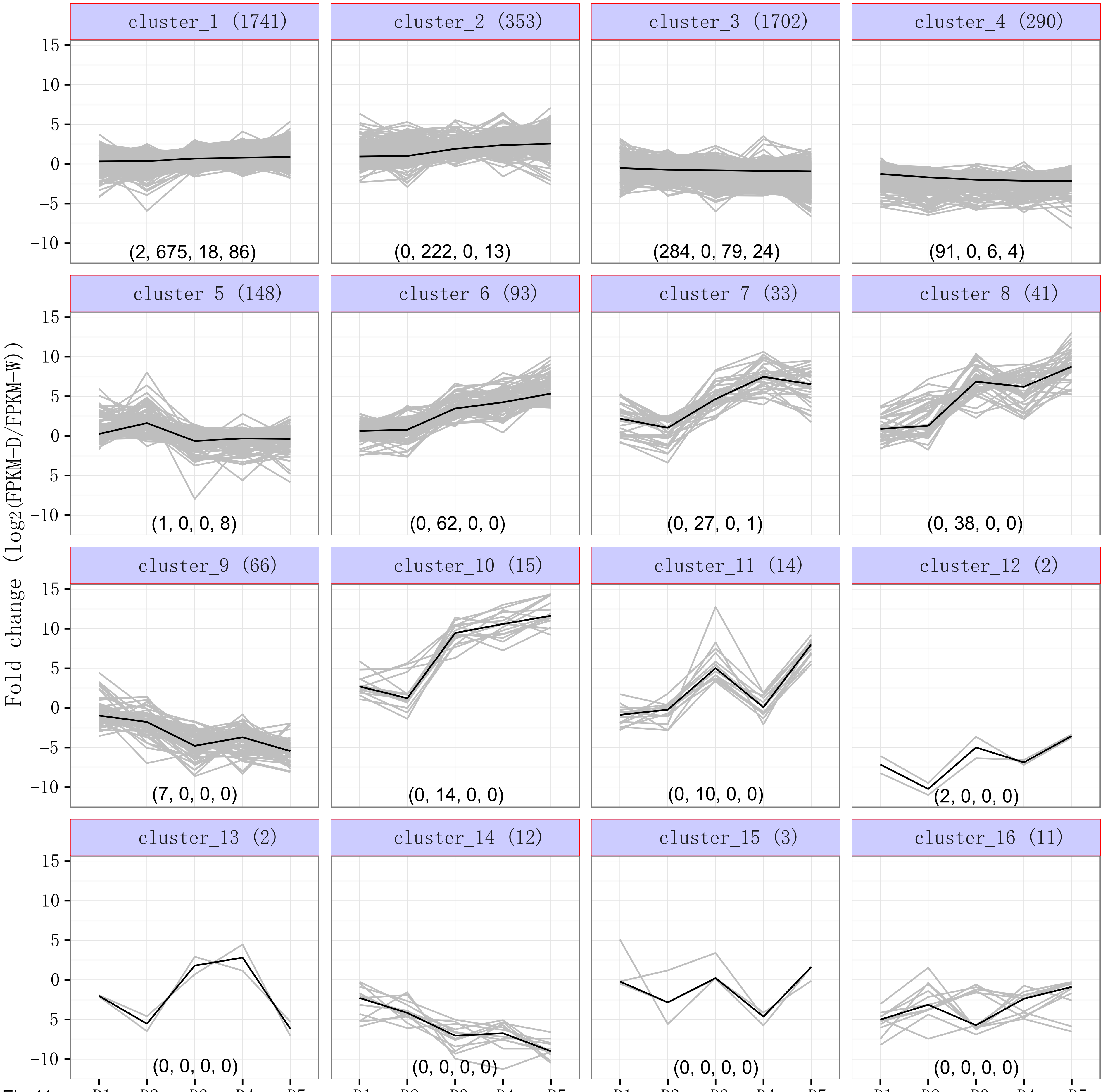

cluster_1 (1741)
cluster_2 (353)
cluster_3 (1702)
cluster_4 (290)
cluster_5 (148)
cluster_6 (93)
cluster_7 (33)
cluster_8 (41)
cluster_9 (66)
cluster_10 (15)
cluster_11 (14)
cluster_12 (2)
cluster_13 (2)
cluster_14 (12)
cluster_15 (3)
cluster_16 (11)
(2, 675, 18, 86)
(0, 222, 0, 13)
(284, 0, 79, 24)
(91, 0, 6, 4)
(1, 0, 0, 8)
(0, 62, 0, 0)
(0, 27, 0, 1)
(0, 38, 0, 0)
(7, 0, 0, 0)
(0, 14, 0, 0)
(0, 10, 0, 0)
(2, 0, 0, 0)
(0, 0, 0, 0)
(0, 0, 0, 0)
(0, 0, 0, 0)
(0, 0, 0, 0)
Fold change (log2(FPKM-D/FPKM-W))
15
10
5
0
−5
−10
D1
D2
D3
D4
D5

Fig 11

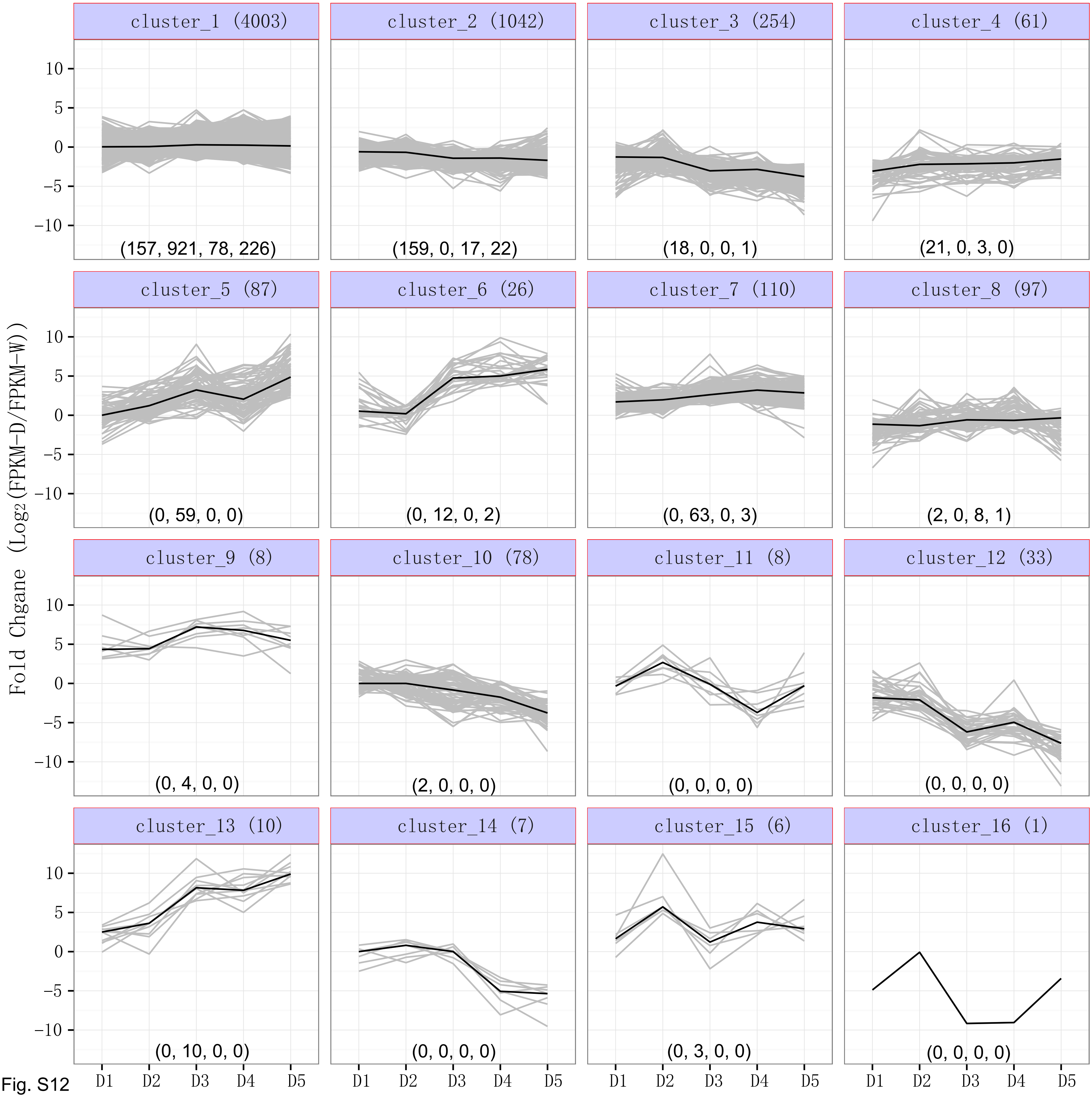
cluster_1 (4003)
cluster_2 (1042)
cluster_3 (254)
cluster_4 (61)
(157, 921, 78, 226)
(159, 0, 17, 22)
(18, 0, 0, 1)
(21, 0, 3, 0)
cluster_5 (87)
cluster_6 (26)
cluster_7 (110)
cluster_8 (97)
(0, 59, 0, 0)
(0, 12, 0, 2)
(0, 63, 0, 3)
(2, 0, 8, 1)
cluster_9 (8)
cluster_10 (78)
cluster_11 (8)
cluster_12 (33)
(0, 4, 0, 0)
(2, 0, 0, 0)
(0, 0, 0, 0)
(0, 0, 0, 0)
cluster_13 (10)
cluster_14 (7)
cluster_15 (6)
cluster_16 (1)
(0, 10, 0, 0)
(0, 0, 0, 0)
(0, 3, 0, 0)
(0, 0, 0, 0)
Fold Chgane (Log2(FPKM-D/FPKM-W))
10
5
0
−5
−10
D1
D2
D3
D4
D5
Fig. S12

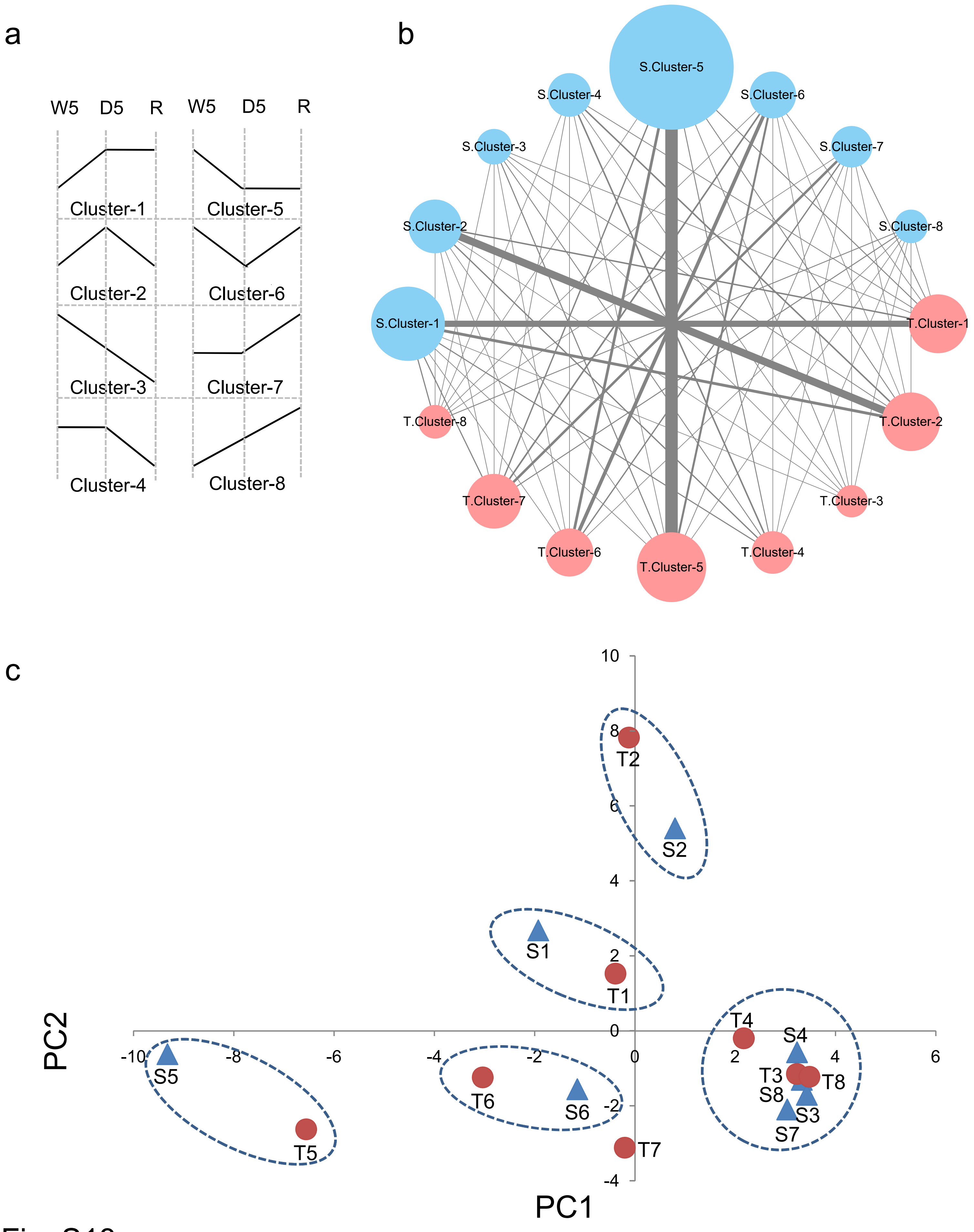
a
b
c
W5
D5
R
Cluster-1
Cluster-2
Cluster-3
Cluster-4
Cluster-5
Cluster-6
Cluster-7
Cluster-8
S.Cluster-1
S.Cluster-2
S.Cluster-3
S.Cluster-4
S.Cluster-5
S.Cluster-6
S.Cluster-7
S.Cluster-8
T.Cluster-1
T.Cluster-2
T.Cluster-3
T.Cluster-4
T.Cluster-5
T.Cluster-6
T.Cluster-7
T.Cluster-8
PC1
PC2
S1
S2
S3
S4
S5
S6
S7
S8
T1
T2
T3
T4
T5
T6
T7
T8

Fig. S13

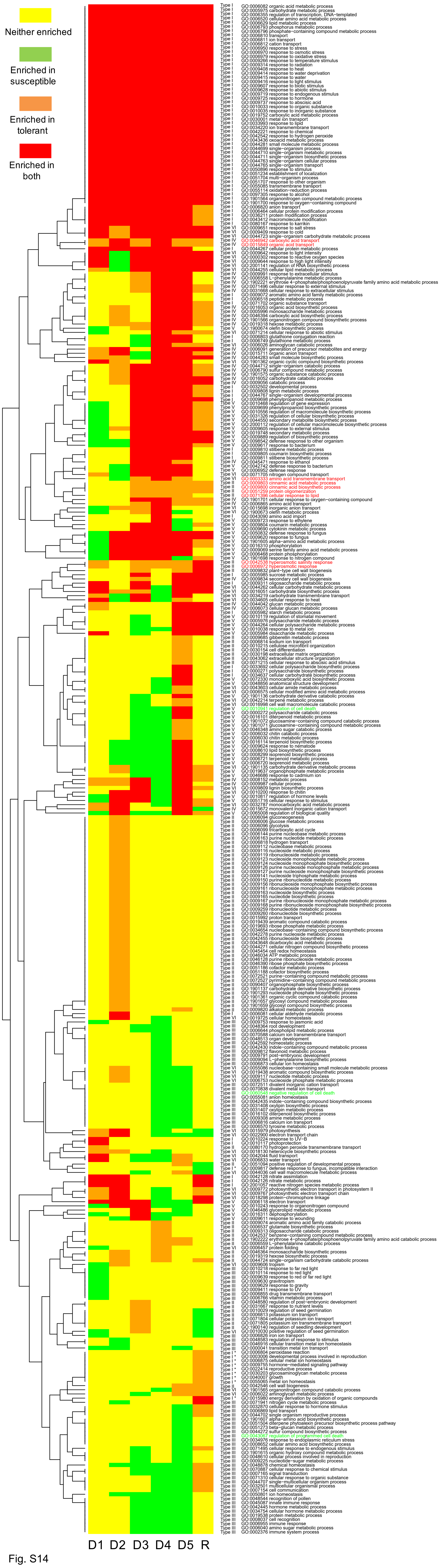

Neither enriched
Enriched in susceptible
Enriched in tolerant
Enriched in both
D1 D2 D3 D4 D5 R

Fig. S14

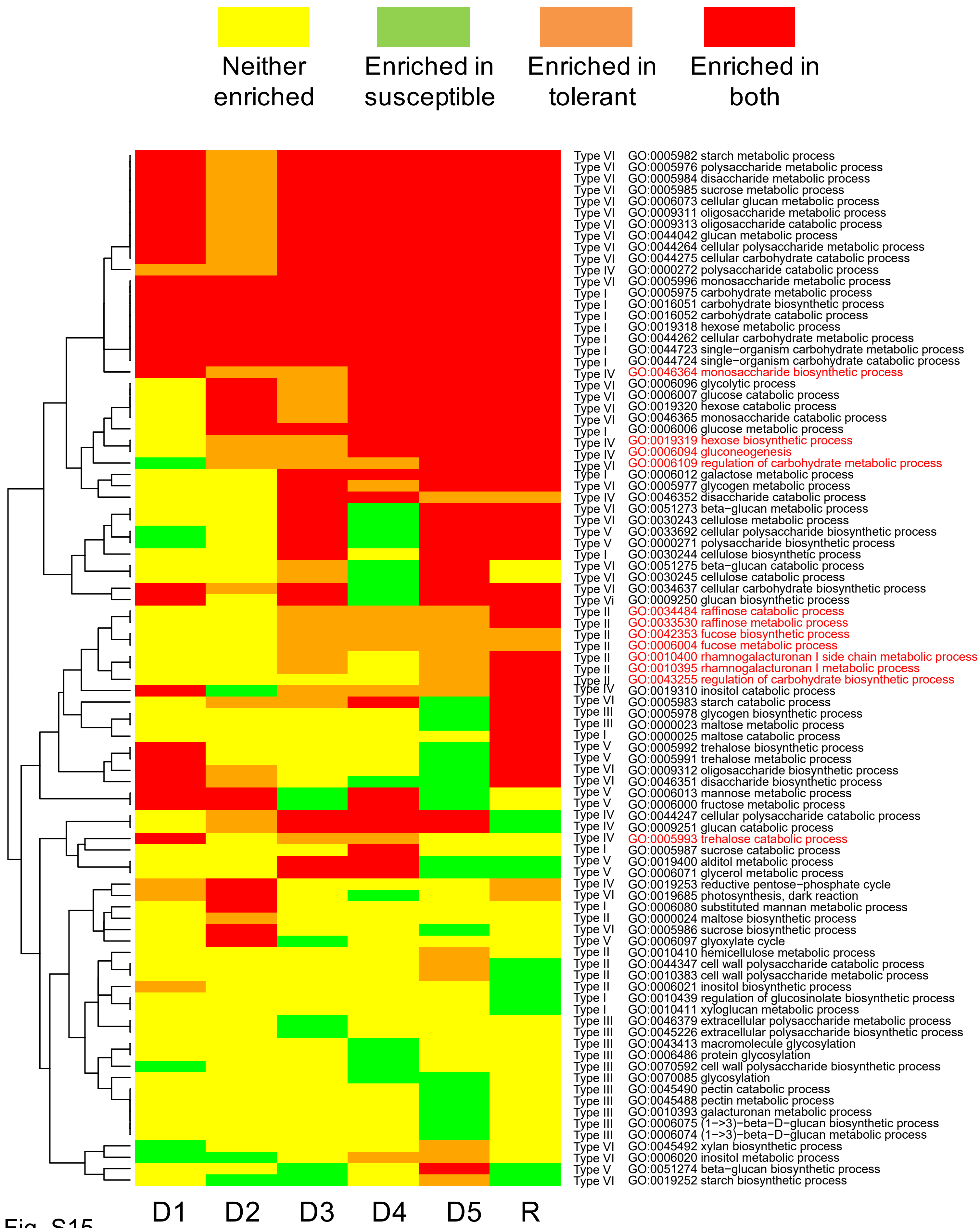
Neither enriched
Enriched in susceptible
Enriched in tolerant
Enriched in both
Type VI GO:0005982 starch metabolic process
Type VI GO:0005976 polysaccharide metabolic process
Type VI GO:0005984 disaccharide metabolic process
Type VI GO:0005985 sucrose metabolic process
Type VI GO:0006073 cellular glucan metabolic process
Type VI GO:0009311 oligosaccharide metabolic process
Type VI GO:0009313 oligosaccharide catabolic process
Type VI GO:0044042 glucan metabolic process
Type VI GO:0044264 cellular polysaccharide metabolic process
Type VI GO:0044275 cellular carbohydrate catabolic process
Type IV GO:0000272 polysaccharide catabolic process
Type VI GO:0005996 monosaccharide metabolic process
Type I GO:0005975 carbohydrate metabolic process
Type I GO:0016051 carbohydrate biosynthetic process
Type I GO:0016052 carbohydrate catabolic process
Type I GO:0019318 hexose metabolic process
Type I GO:0044262 cellular carbohydrate metabolic process
Type I GO:0044723 single−organism carbohydrate metabolic process
Type I GO:0044724 single−organism carbohydrate catabolic process
Type IV GO:0046364 monosaccharide biosynthetic process
Type VI GO:0006096 glycolytic process
Type VI GO:0006007 glucose catabolic process
Type VI GO:0019320 hexose catabolic process
Type VI GO:0046365 monosaccharide catabolic process
Type I GO:0006006 glucose metabolic process
Type IV GO:0019319 hexose biosynthetic process
Type IV GO:0006094 gluconeogenesis
Type VI GO:0006109 regulation of carbohydrate metabolic process
Type I GO:0006012 galactose metabolic process
Type VI GO:0005977 glycogen metabolic process
Type IV GO:0046352 disaccharide catabolic process
Type VI GO:0051273 beta−glucan metabolic process
Type VI GO:0030243 cellulose metabolic process
Type V GO:0033692 cellular polysaccharide biosynthetic process
Type V GO:0000271 polysaccharide biosynthetic process
Type I GO:0030244 cellulose biosynthetic process
Type VI GO:0051275 beta−glucan catabolic process
Type VI GO:0030245 cellulose catabolic process
Type VI GO:0034637 cellular carbohydrate biosynthetic process
Type Vi GO:0009250 glucan biosynthetic process
Type II GO:0034484 raffinose catabolic process
Type II GO:0033530 raffinose metabolic process
Type II GO:0042353 fucose biosynthetic process
Type II GO:0006004 fucose metabolic process
Type II GO:0010400 rhamnogalacturonan I side chain metabolic process
Type II GO:0010395 rhamnogalacturonan I metabolic process
Type II GO:0043255 regulation of carbohydrate biosynthetic process
Type IV GO:0019310 inositol catabolic process
Type VI GO:0005983 starch catabolic process
Type III GO:0005978 glycogen biosynthetic process
Type III GO:0000023 maltose metabolic process
Type I GO:0000025 maltose catabolic process
Type V GO:0005992 trehalose biosynthetic process
Type V GO:0005991 trehalose metabolic process
Type VI GO:0009312 oligosaccharide biosynthetic process
Type VI GO:0046351 disaccharide biosynthetic process
Type V GO:0006013 mannose metabolic process
Type V GO:0006000 fructose metabolic process
Type IV GO:0044247 cellular polysaccharide catabolic process
Type IV GO:0009251 glucan catabolic process
Type IV GO:0005993 trehalose catabolic process
Type I GO:0005987 sucrose catabolic process
Type V GO:0019400 alditol metabolic process
Type V GO:0006071 glycerol metabolic process
Type IV GO:0019253 reductive pentose−phosphate cycle
Type VI GO:0019685 photosynthesis, dark reaction
Type I GO:0006080 substituted mannan metabolic process
Type II GO:0000024 maltose biosynthetic process
Type VI GO:0005986 sucrose biosynthetic process
Type V GO:0006097 glyoxylate cycle
Type II GO:0010410 hemicellulose metabolic process
Type II GO:0044347 cell wall polysaccharide catabolic process
Type II GO:0010383 cell wall polysaccharide metabolic process
Type II GO:0006021 inositol biosynthetic process
Type I GO:0010439 regulation of glucosinolate biosynthetic process
Type I GO:0010411 xyloglucan metabolic process
Type III GO:0046379 extracellular polysaccharide metabolic process
Type III GO:0045226 extracellular polysaccharide biosynthetic process
Type III GO:0043413 macromolecule glycosylation
Type III GO:0006486 protein glycosylation
Type III GO:0070592 cell wall polysaccharide biosynthetic process
Type III GO:0070085 glycosylation
Type III GO:0045490 pectin catabolic process
Type III GO:0045488 pectin metabolic process
Type III GO:0010393 galacturonan metabolic process
Type III GO:0006075 (1−>3)−beta−D−glucan biosynthetic process
Type III GO:0006074 (1−>3)−beta−D−glucan metabolic process
Type VI GO:0045492 xylan biosynthetic process
Type VI GO:0006020 inositol metabolic process
Type V GO:0051274 beta−glucan biosynthetic process
Type VI GO:0019252 starch biosynthetic process
D1
D2
D3
D4
D5
R

Fig. S15

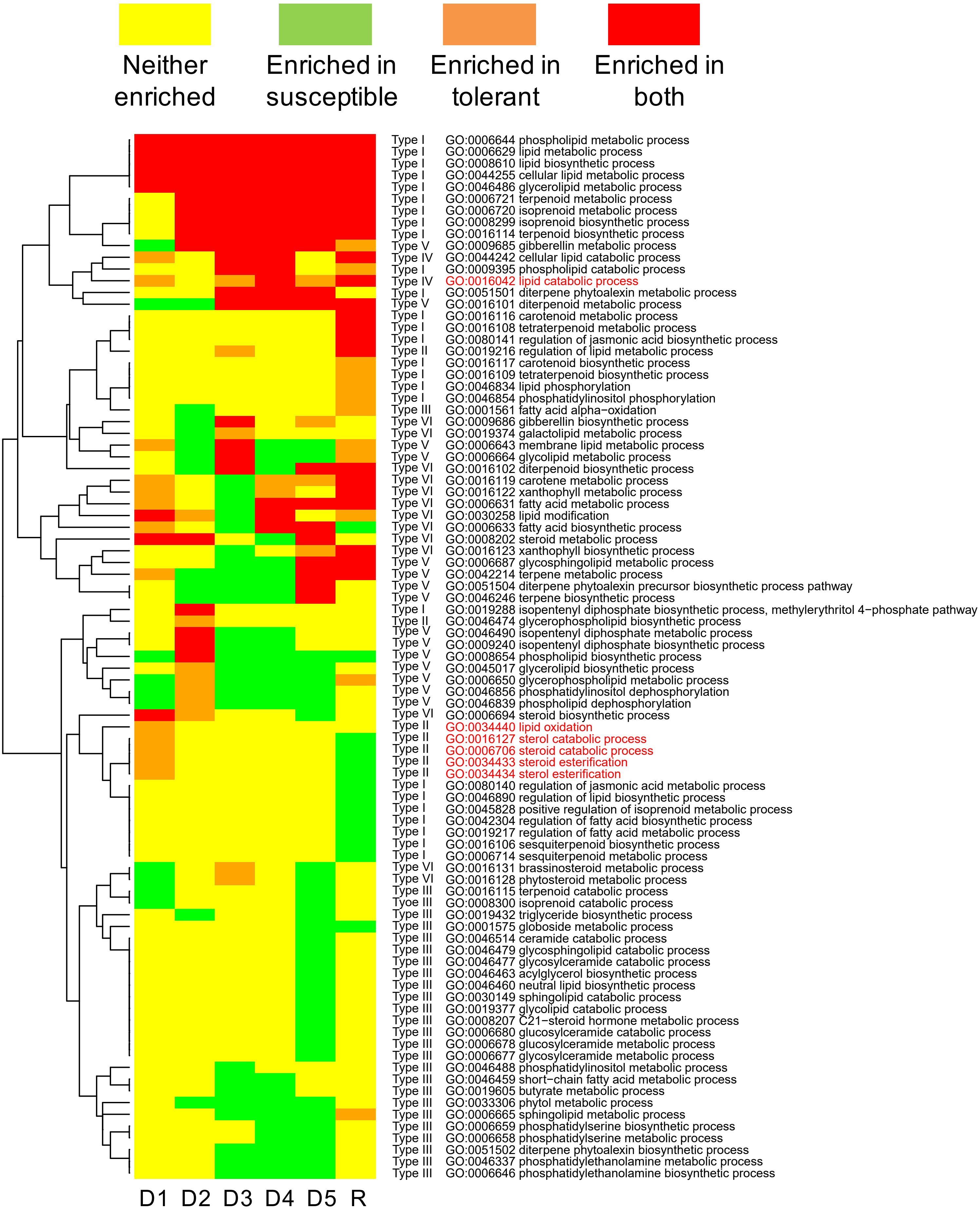
Neither enriched
Enriched in susceptible
Enriched in tolerant
Enriched in both
Type I GO:0006644 phospholipid metabolic process
Type I GO:0006629 lipid metabolic process
Type I GO:0008610 lipid biosynthetic process
Type I GO:0044255 cellular lipid metabolic process
Type I GO:0046486 glycerolipid metabolic process
Type I GO:0006721 terpenoid metabolic process
Type I GO:0006720 isoprenoid metabolic process
Type I GO:0008299 isoprenoid biosynthetic process
Type I GO:0016114 terpenoid biosynthetic process
Type V GO:0009685 gibberellin metabolic process
Type IV GO:0044242 cellular lipid catabolic process
Type I GO:0009395 phospholipid catabolic process
Type IV GO:0016042 lipid catabolic process
Type I GO:0051501 diterpene phytoalexin metabolic process
Type V GO:0016101 diterpenoid metabolic process
Type I GO:0016116 carotenoid metabolic process
Type I GO:0016108 tetraterpenoid metabolic process
Type I GO:0080141 regulation of jasmonic acid biosynthetic process
Type II GO:0019216 regulation of lipid metabolic process
Type I GO:0016117 carotenoid biosynthetic process
Type I GO:0016109 tetraterpenoid biosynthetic process
Type I GO:0046834 lipid phosphorylation
Type I GO:0046854 phosphatidylinositol phosphorylation
Type III GO:0001561 fatty acid alpha−oxidation
Type VI GO:0009686 gibberellin biosynthetic process
Type VI GO:0019374 galactolipid metabolic process
Type V GO:0006643 membrane lipid metabolic process
Type V GO:0006664 glycolipid metabolic process
Type VI GO:0016102 diterpenoid biosynthetic process
Type VI GO:0016119 carotene metabolic process
Type VI GO:0016122 xanthophyll metabolic process
Type VI GO:0006631 fatty acid metabolic process
Type VI GO:0030258 lipid modification
Type VI GO:0006633 fatty acid biosynthetic process
Type VI GO:0008202 steroid metabolic process
Type VI GO:0016123 xanthophyll biosynthetic process
Type V GO:0006687 glycosphingolipid metabolic process
Type V GO:0042214 terpene metabolic process
Type V GO:0051504 diterpene phytoalexin precursor biosynthetic process pathway
Type V GO:0046246 terpene biosynthetic process
Type I GO:0019288 isopentenyl diphosphate biosynthetic process, methylerythritol 4−phosphate pathway
Type II GO:0046474 glycerophospholipid biosynthetic process
Type V GO:0046490 isopentenyl diphosphate metabolic process
Type V GO:0009240 isopentenyl diphosphate biosynthetic process
Type V GO:0008654 phospholipid biosynthetic process
Type V GO:0045017 glycerolipid biosynthetic process
Type V GO:0006650 glycerophospholipid metabolic process
Type V GO:0046856 phosphatidylinositol dephosphorylation
Type V GO:0046839 phospholipid dephosphorylation
Type VI GO:0006694 steroid biosynthetic process
Type II GO:0034440 lipid oxidation
Type II GO:0016127 sterol catabolic process
Type II GO:0006706 steroid catabolic process
Type II GO:0034433 steroid esterification
Type II GO:0034434 sterol esterification
Type I GO:0080140 regulation of jasmonic acid metabolic process
Type I GO:0046890 regulation of lipid biosynthetic process
Type I GO:0045828 positive regulation of isoprenoid metabolic process
Type I GO:0042304 regulation of fatty acid biosynthetic process
Type I GO:0019217 regulation of fatty acid metabolic process
Type I GO:0016106 sesquiterpenoid biosynthetic process
Type I GO:0006714 sesquiterpenoid metabolic process
Type VI GO:0016131 brassinosteroid metabolic process
Type VI GO:0016128 phytosteroid metabolic process
Type III GO:0016115 terpenoid catabolic process
Type III GO:0008300 isoprenoid catabolic process
Type III GO:0019432 triglyceride biosynthetic process
Type III GO:0001575 globoside metabolic process
Type III GO:0046514 ceramide catabolic process
Type III GO:0046479 glycosphingolipid catabolic process
Type III GO:0046477 glycosylceramide catabolic process
Type III GO:0046463 acylglycerol biosynthetic process
Type III GO:0046460 neutral lipid biosynthetic process
Type III GO:0030149 sphingolipid catabolic process
Type III GO:0019377 glycolipid catabolic process
Type III GO:0008207 C21−steroid hormone metabolic process
Type III GO:0006680 glucosylceramide catabolic process
Type III GO:0006678 glucosylceramide metabolic process
Type III GO:0006677 glycosylceramide metabolic process
Type III GO:0046488 phosphatidylinositol metabolic process
Type III GO:0046459 short−chain fatty acid metabolic process
Type III GO:0019605 butyrate metabolic process
Type III GO:0033306 phytol metabolic process
Type III GO:0006665 sphingolipid metabolic process
Type III GO:0006659 phosphatidylserine biosynthetic process
Type III GO:0006658 phosphatidylserine metabolic process
Type III GO:0051502 diterpene phytoalexin biosynthetic process
Type III GO:0046337 phosphatidylethanolamine metabolic process
Type III GO:0006646 phosphatidylethanolamine biosynthetic process
D1 D2 D3 D4 D5 R

Fig. S16

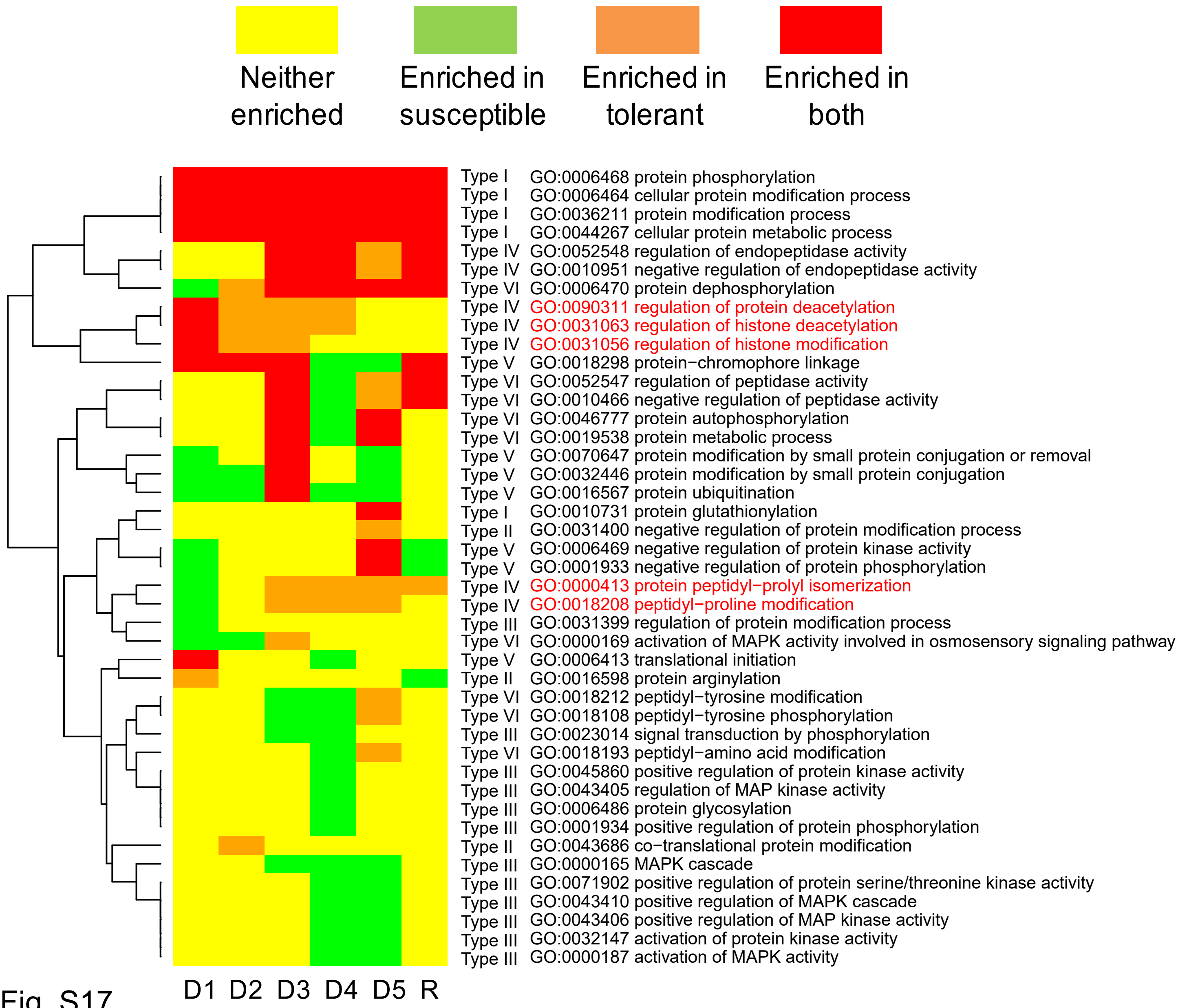

Neither enriched
Enriched in susceptible
Enriched in tolerant
Enriched in both
Type I GO:0006468 protein phosphorylation
Type I GO:0006464 cellular protein modification process
Type I GO:0036211 protein modification process
Type I GO:0044267 cellular protein metabolic process
Type IV GO:0052548 regulation of endopeptidase activity
Type IV GO:0010951 negative regulation of endopeptidase activity
Type VI GO:0006470 protein dephosphorylation
Type IV GO:0090311 regulation of protein deacetylation
Type IV GO:0031063 regulation of histone deacetylation
Type IV GO:0031056 regulation of histone modification
Type V GO:0018298 protein−chromophore linkage
Type VI GO:0052547 regulation of peptidase activity
Type VI GO:0010466 negative regulation of peptidase activity
Type VI GO:0046777 protein autophosphorylation
Type VI GO:0019538 protein metabolic process
Type V GO:0070647 protein modification by small protein conjugation or removal
Type V GO:0032446 protein modification by small protein conjugation
Type V GO:0016567 protein ubiquitination
Type I GO:0010731 protein glutathionylation
Type II GO:0031400 negative regulation of protein modification process
Type V GO:0006469 negative regulation of protein kinase activity
Type V GO:0001933 negative regulation of protein phosphorylation
Type IV GO:0000413 protein peptidyl−prolyl isomerization
Type IV GO:0018208 peptidyl−proline modification
Type III GO:0031399 regulation of protein modification process
Type VI GO:0000169 activation of MAPK activity involved in osmosensory signaling pathway
Type V GO:0006413 translational initiation
Type II GO:0016598 protein arginylation
Type VI GO:0018212 peptidyl−tyrosine modification
Type VI GO:0018108 peptidyl−tyrosine phosphorylation
Type III GO:0023014 signal transduction by phosphorylation
Type VI GO:0018193 peptidyl−amino acid modification
Type III GO:0045860 positive regulation of protein kinase activity
Type III GO:0043405 regulation of MAP kinase activity
Type III GO:0006486 protein glycosylation
Type III GO:0001934 positive regulation of protein phosphorylation
Type II GO:0043686 co−translational protein modification
Type III GO:0000165 MAPK cascade
Type III GO:0071902 positive regulation of protein serine/threonine kinase activity
Type III GO:0043410 positive regulation of MAPK cascade
Type III GO:0043406 positive regulation of MAP kinase activity
Type III GO:0032147 activation of protein kinase activity
Type III GO:0000187 activation of MAPK activity
D1 D2 D3 D4 D5 R

Fig. S17

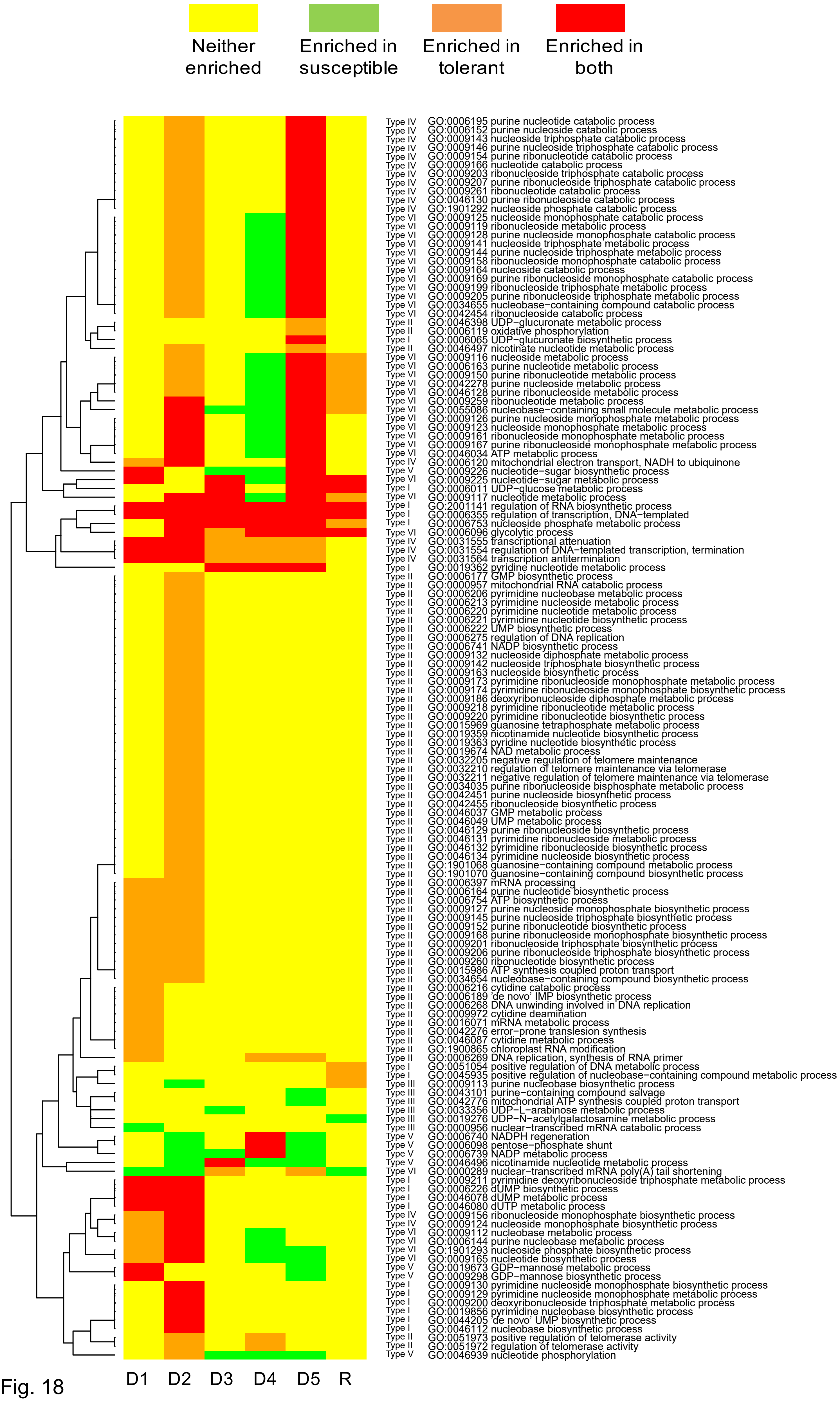

Neither enriched
Enriched in susceptible
Enriched in tolerant
Enriched in both
Type IV GO:0006195 purine nucleotide catabolic process
Type IV GO:0006152 purine nucleoside catabolic process
Type IV GO:0009143 nucleoside triphosphate catabolic process
Type IV GO:0009146 purine nucleoside triphosphate catabolic process
Type IV GO:0009154 purine ribonucleotide catabolic process
Type IV GO:0009166 nucleotide catabolic process
Type IV GO:0009203 ribonucleoside triphosphate catabolic process
Type IV GO:0009207 purine ribonucleoside triphosphate catabolic process
Type IV GO:0009261 ribonucleotide catabolic process
Type IV GO:0046130 purine ribonucleoside catabolic process
Type IV GO:1901292 nucleoside phosphate catabolic process
Type VI GO:0009125 nucleoside monophosphate catabolic process
Type VI GO:0009119 ribonucleoside metabolic process
Type VI GO:0009128 purine nucleoside monophosphate catabolic process
Type VI GO:0009141 nucleoside triphosphate metabolic process
Type VI GO:0009144 purine nucleoside triphosphate metabolic process
Type VI GO:0009158 ribonucleoside monophosphate catabolic process
Type VI GO:0009164 nucleoside catabolic process
Type VI GO:0009169 purine ribonucleoside monophosphate catabolic process
Type VI GO:0009199 ribonucleoside triphosphate metabolic process
Type VI GO:0009205 purine ribonucleoside triphosphate metabolic process
Type VI GO:0034655 nucleobase−containing compound catabolic process
Type VI GO:0042454 ribonucleoside catabolic process
Type II GO:0046398 UDP−glucuronate metabolic process
Type II GO:0006119 oxidative phosphorylation
Type I GO:0006065 UDP−glucuronate biosynthetic process
Type II GO:0046497 nicotinate nucleotide metabolic process
Type VI GO:0009116 nucleoside metabolic process
Type VI GO:0006163 purine nucleotide metabolic process
Type VI GO:0009150 purine ribonucleotide metabolic process
Type VI GO:0042278 purine nucleoside metabolic process
Type VI GO:0046128 purine ribonucleoside metabolic process
Type VI GO:0009259 ribonucleotide metabolic process
Type VI GO:0055086 nucleobase−containing small molecule metabolic process
Type VI GO:0009126 purine nucleoside monophosphate metabolic process
Type VI GO:0009123 nucleoside monophosphate metabolic process
Type VI GO:0009161 ribonucleoside monophosphate metabolic process
Type VI GO:0009167 purine ribonucleoside monophosphate metabolic process
Type VI GO:0046034 ATP metabolic process
Type IV GO:0006120 mitochondrial electron transport, NADH to ubiquinone
Type V GO:0009226 nucleotide−sugar biosynthetic process
Type VI GO:0009225 nucleotide−sugar metabolic process
Type I GO:0006011 UDP−glucose metabolic process
Type VI GO:0009117 nucleotide metabolic process
Type I GO:2001141 regulation of RNA biosynthetic process
Type I GO:0006355 regulation of transcription, DNA−templated
Type I GO:0006753 nucleoside phosphate metabolic process
Type VI GO:0006096 glycolytic process
Type IV GO:0031555 transcriptional attenuation
Type IV GO:0031554 regulation of DNA−templated transcription, termination
Type IV GO:0031564 transcription antitermination
Type I GO:0019362 pyridine nucleotide metabolic process
Type II GO:0006177 GMP biosynthetic process
Type II GO:0000957 mitochondrial RNA catabolic process
Type II GO:0006206 pyrimidine nucleobase metabolic process
Type II GO:0006213 pyrimidine nucleoside metabolic process
Type II GO:0006220 pyrimidine nucleotide metabolic process
Type II GO:0006221 pyrimidine nucleotide biosynthetic process
Type II GO:0006222 UMP biosynthetic process
Type II GO:0006275 regulation of DNA replication
Type II GO:0006741 NADP biosynthetic process
Type II GO:0009132 nucleoside diphosphate metabolic process
Type II GO:0009142 nucleoside triphosphate biosynthetic process
Type II GO:0009163 nucleoside biosynthetic process
Type II GO:0009173 pyrimidine ribonucleoside monophosphate metabolic process
Type II GO:0009174 pyrimidine ribonucleoside monophosphate biosynthetic process
Type II GO:0009186 deoxyribonucleoside diphosphate metabolic process
Type II GO:0009218 pyrimidine ribonucleotide metabolic process
Type II GO:0009220 pyrimidine ribonucleotide biosynthetic process
Type II GO:0015969 guanosine tetraphosphate metabolic process
Type II GO:0019359 nicotinamide nucleotide biosynthetic process
Type II GO:0019363 pyridine nucleotide biosynthetic process
Type II GO:0019674 NAD metabolic process
Type II GO:0032205 negative regulation of telomere maintenance
Type II GO:0032210 regulation of telomere maintenance via telomerase
Type II GO:0032211 negative regulation of telomere maintenance via telomerase
Type II GO:0034035 purine ribonucleoside bisphosphate metabolic process
Type II GO:0042451 purine nucleoside biosynthetic process
Type II GO:0042455 ribonucleoside biosynthetic process
Type II GO:0046037 GMP metabolic process
Type II GO:0046049 UMP metabolic process
Type II GO:0046129 purine ribonucleoside biosynthetic process
Type II GO:0046131 pyrimidine ribonucleoside metabolic process
Type II GO:0046132 pyrimidine ribonucleoside biosynthetic process
Type II GO:0046134 pyrimidine nucleoside biosynthetic process
Type II GO:1901068 guanosine−containing compound metabolic process
Type II GO:1901070 guanosine−containing compound biosynthetic process
Type II GO:0006397 mRNA processing
Type II GO:0006164 purine nucleotide biosynthetic process
Type II GO:0006754 ATP biosynthetic process
Type II GO:0009127 purine nucleoside monophosphate biosynthetic process
Type II GO:0009145 purine nucleoside triphosphate biosynthetic process
Type II GO:0009152 purine ribonucleotide biosynthetic process
Type II GO:0009168 purine ribonucleoside monophosphate biosynthetic process
Type II GO:0009201 ribonucleoside triphosphate biosynthetic process
Type II GO:0009206 purine ribonucleoside triphosphate biosynthetic process
Type II GO:0009260 ribonucleotide biosynthetic process
Type II GO:0015986 ATP synthesis coupled proton transport
Type II GO:0034654 nucleobase−containing compound biosynthetic process
Type II GO:0006216 cytidine catabolic process
Type II GO:0006189 'de novo' IMP biosynthetic process
Type II GO:0006268 DNA unwinding involved in DNA replication
Type II GO:0009972 cytidine deamination
Type II GO:0016071 mRNA metabolic process
Type II GO:0042276 error−prone translesion synthesis
Type II GO:0046087 cytidine metabolic process
Type II GO:1900865 chloroplast RNA modification
Type II GO:0006269 DNA replication, synthesis of RNA primer
Type I GO:0051054 positive regulation of DNA metabolic process
Type I GO:0045935 positive regulation of nucleobase−containing compound metabolic process
Type III GO:0009113 purine nucleobase biosynthetic process
Type III GO:0043101 purine−containing compound salvage
Type III GO:0042776 mitochondrial ATP synthesis coupled proton transport
Type III GO:0033356 UDP−L−arabinose metabolic process
Type III GO:0019276 UDP−N−acetylgalactosamine metabolic process
Type III GO:0000956 nuclear−transcribed mRNA catabolic process
Type V GO:0006740 NADPH regeneration
Type V GO:0006098 pentose−phosphate shunt
Type V GO:0006739 NADP metabolic process
Type V GO:0046496 nicotinamide nucleotide metabolic process
Type VI GO:0000289 nuclear−transcribed mRNA poly(A) tail shortening
Type I GO:0009211 pyrimidine deoxyribonucleoside triphosphate metabolic process
Type I GO:0006226 dUMP biosynthetic process
Type I GO:0046078 dUMP metabolic process
Type I GO:0046080 dUTP metabolic process
Type IV GO:0009156 ribonucleoside monophosphate biosynthetic process
Type IV GO:0009124 nucleoside monophosphate biosynthetic process
Type VI GO:0009112 nucleobase metabolic process
Type VI GO:0006144 purine nucleobase metabolic process
Type VI GO:1901293 nucleoside phosphate biosynthetic process
Type VI GO:0009165 nucleotide biosynthetic process
Type V GO:0019673 GDP−mannose metabolic process
Type V GO:0009298 GDP−mannose biosynthetic process
Type I GO:0009130 pyrimidine nucleoside monophosphate biosynthetic process
Type I GO:0009129 pyrimidine nucleoside monophosphate metabolic process
Type I GO:0009200 deoxyribonucleoside triphosphate metabolic process
Type I GO:0019856 pyrimidine nucleobase biosynthetic process
Type I GO:0044205 'de novo' UMP biosynthetic process
Type I GO:0046112 nucleobase biosynthetic process
Type II GO:0051973 positive regulation of telomerase activity
Type II GO:0051972 regulation of telomerase activity
Type V GO:0046939 nucleotide phosphorylation
D1
D2
D3
D4
D5
R

Fig. 18

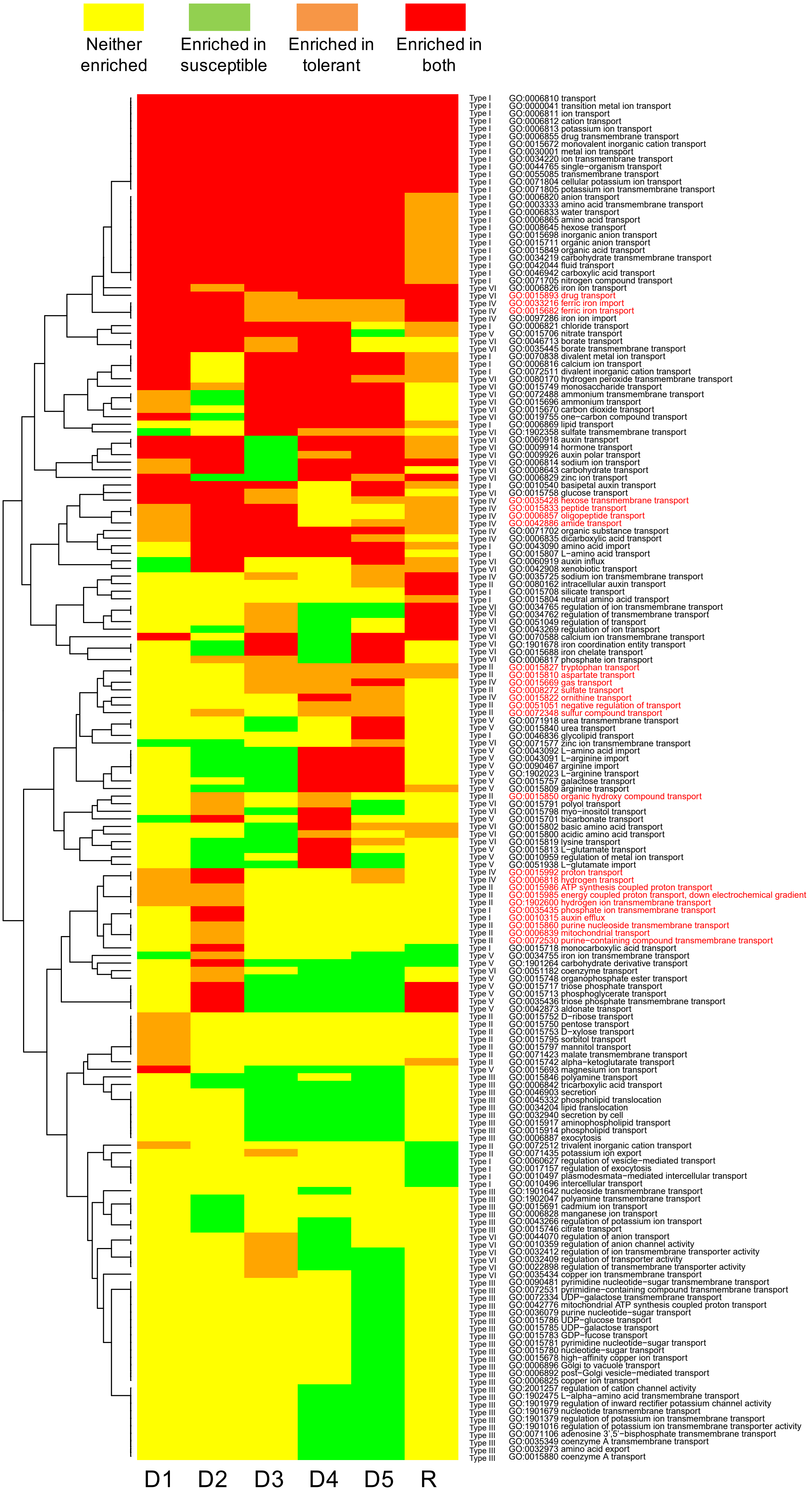
Neither enriched
Enriched in susceptible
Enriched in tolerant
Enriched in both
Type I GO:0006810 transport
Type I GO:0000041 transition metal ion transport
Type I GO:0006811 ion transport
Type I GO:0006812 cation transport
Type I GO:0006813 potassium ion transport
Type I GO:0006855 drug transmembrane transport
Type I GO:0015672 monovalent inorganic cation transport
Type I GO:0030001 metal ion transport
Type I GO:0034220 ion transmembrane transport
Type I GO:0044765 single−organism transport
Type I GO:0055085 transmembrane transport
Type I GO:0071804 cellular potassium ion transport
Type I GO:0071805 potassium ion transmembrane transport
Type I GO:0006820 anion transport
Type I GO:0003333 amino acid transmembrane transport
Type I GO:0006833 water transport
Type I GO:0006865 amino acid transport
Type I GO:0008645 hexose transport
Type I GO:0015698 inorganic anion transport
Type I GO:0015711 organic anion transport
Type I GO:0015849 organic acid transport
Type I GO:0034219 carbohydrate transmembrane transport
Type I GO:0042044 fluid transport
Type I GO:0046942 carboxylic acid transport
Type I GO:0071705 nitrogen compound transport
Type VI GO:0006826 iron ion transport
Type VI GO:0015893 drug transport
Type IV GO:0033216 ferric iron import
Type IV GO:0015682 ferric iron transport
Type IV GO:0097286 iron ion import
Type I GO:0006821 chloride transport
Type V GO:0015706 nitrate transport
Type VI GO:0046713 borate transport
Type VI GO:0035445 borate transmembrane transport
Type I GO:0070838 divalent metal ion transport
Type I GO:0006816 calcium ion transport
Type I GO:0072511 divalent inorganic cation transport
Type VI GO:0080170 hydrogen peroxide transmembrane transport
Type VI GO:0015749 monosaccharide transport
Type VI GO:0072488 ammonium transmembrane transport
Type VI GO:0015696 ammonium transport
Type VI GO:0015670 carbon dioxide transport
Type VI GO:0019755 one−carbon compound transport
Type I GO:0006869 lipid transport
Type VI GO:1902358 sulfate transmembrane transport
Type VI GO:0060918 auxin transport
Type VI GO:0009914 hormone transport
Type VI GO:0009926 auxin polar transport
Type VI GO:0006814 sodium ion transport
Type VI GO:0008643 carbohydrate transport
Type VI GO:0006829 zinc ion transport
Type I GO:0010540 basipetal auxin transport
Type VI GO:0015758 glucose transport
Type IV GO:0035428 hexose transmembrane transport
Type IV GO:0015833 peptide transport
Type IV GO:0006857 oligopeptide transport
Type IV GO:0042886 amide transport
Type IV GO:0071702 organic substance transport
Type IV GO:0006835 dicarboxylic acid transport
Type I GO:0043090 amino acid import
Type I GO:0015807 L−amino acid transport
Type VI GO:0060919 auxin influx
Type VI GO:0042908 xenobiotic transport
Type IV GO:0035725 sodium ion transmembrane transport
Type II GO:0080162 intracellular auxin transport
Type I GO:0015708 silicate transport
Type I GO:0015804 neutral amino acid transport
Type VI GO:0034765 regulation of ion transmembrane transport
Type VI GO:0034762 regulation of transmembrane transport
Type VI GO:0051049 regulation of transport
Type VI GO:0043269 regulation of ion transport
Type VI GO:0070588 calcium ion transmembrane transport
Type VI GO:1901678 iron coordination entity transport
Type VI GO:0015688 iron chelate transport
Type VI GO:0006817 phosphate ion transport
Type II GO:0015827 tryptophan transport
Type II GO:0015810 aspartate transport
Type IV GO:0015669 gas transport
Type II GO:0008272 sulfate transport
Type IV GO:0015822 ornithine transport
Type II GO:0051051 negative regulation of transport
Type II GO:0072348 sulfur compound transport
Type V GO:0071918 urea transmembrane transport
Type V GO:0015840 urea transport
Type I GO:0046836 glycolipid transport
Type VI GO:0071577 zinc ion transmembrane transport
Type V GO:0043092 L−amino acid import
Type V GO:0043091 L−arginine import
Type V GO:0090467 arginine import
Type V GO:1902023 L−arginine transport
Type V GO:0015757 galactose transport
Type V GO:0015809 arginine transport
Type II GO:0015850 organic hydroxy compound transport
Type VI GO:0015791 polyol transport
Type VI GO:0015798 myo−inositol transport
Type V GO:0015701 bicarbonate transport
Type VI GO:0015802 basic amino acid transport
Type VI GO:0015800 acidic amino acid transport
Type VI GO:0015819 lysine transport
Type V GO:0015813 L−glutamate transport
Type V GO:0010959 regulation of metal ion transport
Type V GO:0051938 L−glutamate import
Type IV GO:0015992 proton transport
Type IV GO:0006818 hydrogen transport
Type II GO:0015986 ATP synthesis coupled proton transport
Type II GO:0015985 energy coupled proton transport, down electrochemical gradient
Type II GO:1902600 hydrogen ion transmembrane transport
Type I GO:0035435 phosphate ion transmembrane transport
Type I GO:0010315 auxin efflux
Type II GO:0015860 purine nucleoside transmembrane transport
Type II GO:0006839 mitochondrial transport
Type II GO:0072530 purine−containing compound transmembrane transport
Type I GO:0015718 monocarboxylic acid transport
Type V GO:0034755 iron ion transmembrane transport
Type V GO:1901264 carbohydrate derivative transport
Type VI GO:0051182 coenzyme transport
Type V GO:0015748 organophosphate ester transport
Type V GO:0015717 triose phosphate transport
Type V GO:0015713 phosphoglycerate transport
Type V GO:0035436 triose phosphate transmembrane transport
Type V GO:0042873 aldonate transport
Type II GO:0015752 D−ribose transport
Type II GO:0015750 pentose transport
Type II GO:0015753 D−xylose transport
Type II GO:0015795 sorbitol transport
Type II GO:0015797 mannitol transport
Type II GO:0071423 malate transmembrane transport
Type II GO:0015742 alpha−ketoglutarate transport
Type V GO:0015693 magnesium ion transport
Type III GO:0015846 polyamine transport
Type III GO:0006842 tricarboxylic acid transport
Type III GO:0046903 secretion
Type III GO:0045332 phospholipid translocation
Type III GO:0034204 lipid translocation
Type III GO:0032940 secretion by cell
Type III GO:0015917 aminophospholipid transport
Type III GO:0015914 phospholipid transport
Type III GO:0006887 exocytosis
Type II GO:0072512 trivalent inorganic cation transport
Type II GO:0071435 potassium ion export
Type I GO:0060627 regulation of vesicle−mediated transport
Type I GO:0017157 regulation of exocytosis
Type I GO:0010497 plasmodesmata−mediated intercellular transport
Type I GO:0010496 intercellular transport
Type III GO:1901642 nucleoside transmembrane transport
Type III GO:1902047 polyamine transmembrane transport
Type III GO:0015691 cadmium ion transport
Type III GO:0006828 manganese ion transport
Type III GO:0043266 regulation of potassium ion transport
Type III GO:0015746 citrate transport
Type VI GO:0044070 regulation of anion transport
Type VI GO:0010359 regulation of anion channel activity
Type VI GO:0032412 regulation of ion transmembrane transporter activity
Type VI GO:0032409 regulation of transporter activity
Type VI GO:0022898 regulation of transmembrane transporter activity
Type VI GO:0035434 copper ion transmembrane transport
Type III GO:0090481 pyrimidine nucleotide−sugar transmembrane transport
Type III GO:0072531 pyrimidine−containing compound transmembrane transport
Type III GO:0072334 UDP−galactose transmembrane transport
Type III GO:0042776 mitochondrial ATP synthesis coupled proton transport
Type III GO:0036079 purine nucleotide−sugar transport
Type III GO:0015786 UDP−glucose transport
Type III GO:0015785 UDP−galactose transport
Type III GO:0015783 GDP−fucose transport
Type III GO:0015781 pyrimidine nucleotide−sugar transport
Type III GO:0015780 nucleotide−sugar transport
Type III GO:0015678 high−affinity copper ion transport
Type III GO:0006896 Golgi to vacuole transport
Type III GO:0006892 post−Golgi vesicle−mediated transport
Type III GO:0006825 copper ion transport
Type III GO:2001257 regulation of cation channel activity
Type III GO:1902475 L−alpha−amino acid transmembrane transport
Type III GO:1901979 regulation of inward rectifier potassium channel activity
Type III GO:1901679 nucleotide transmembrane transport
Type III GO:1901379 regulation of potassium ion transmembrane transport
Type III GO:1901016 regulation of potassium ion transmembrane transporter activity
Type III GO:0071106 adenosine 3’,5’−bisphosphate transmembrane transport
Type III GO:0035349 coenzyme A transmembrane transport
Type III GO:0032973 amino acid export
Type III GO:0015880 coenzyme A transport
D1
D2
D3
D4
D5
R

Fig. S19

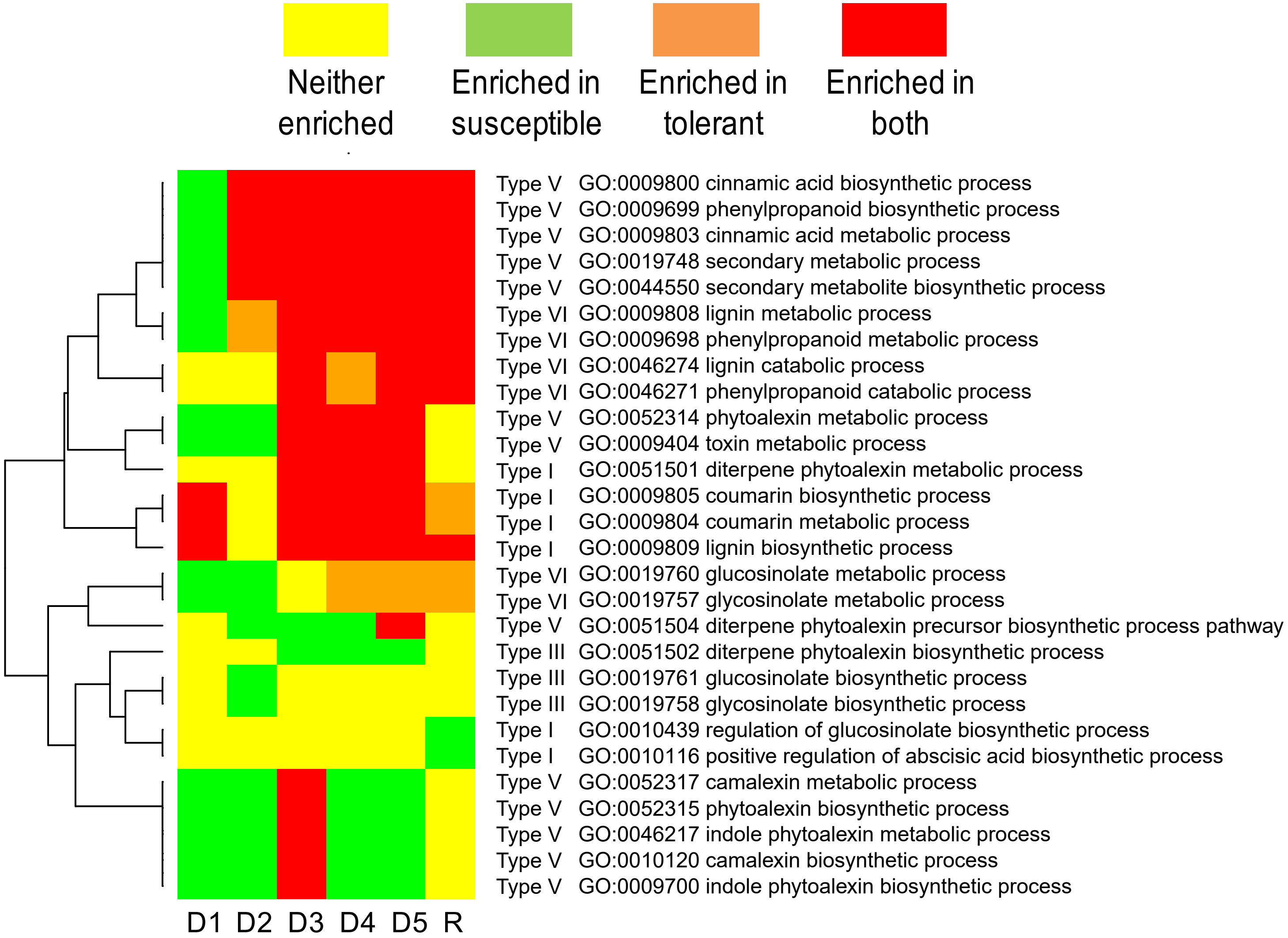
Neither enriched
Enriched in susceptible
Enriched in tolerant
Enriched in both
Type V GO:0009800 cinnamic acid biosynthetic process
Type V GO:0009699 phenylpropanoid biosynthetic process
Type V GO:0009803 cinnamic acid metabolic process
Type V GO:0019748 secondary metabolic process
Type V GO:0044550 secondary metabolite biosynthetic process
Type VI GO:0009808 lignin metabolic process
Type VI GO:0009698 phenylpropanoid metabolic process
Type VI GO:0046274 lignin catabolic process
Type VI GO:0046271 phenylpropanoid catabolic process
Type V GO:0052314 phytoalexin metabolic process
Type V GO:0009404 toxin metabolic process
Type I GO:0051501 diterpene phytoalexin metabolic process
Type I GO:0009805 coumarin biosynthetic process
Type I GO:0009804 coumarin metabolic process
Type I GO:0009809 lignin biosynthetic process
Type VI GO:0019760 glucosinolate metabolic process
Type VI GO:0019757 glycosinolate metabolic process
Type V GO:0051504 diterpene phytoalexin precursor biosynthetic process pathway
Type III GO:0051502 diterpene phytoalexin biosynthetic process
Type III GO:0019761 glucosinolate biosynthetic process
Type III GO:0019758 glycosinolate biosynthetic process
Type I GO:0010439 regulation of glucosinolate biosynthetic process
Type I GO:0010116 positive regulation of abscisic acid biosynthetic process
Type V GO:0052317 camalexin metabolic process
Type V GO:0052315 phytoalexin biosynthetic process
Type V GO:0046217 indole phytoalexin metabolic process
Type V GO:0010120 camalexin biosynthetic process
Type V GO:0009700 indole phytoalexin biosynthetic process
D1 D2 D3 D4 D5 R

Fig. S20

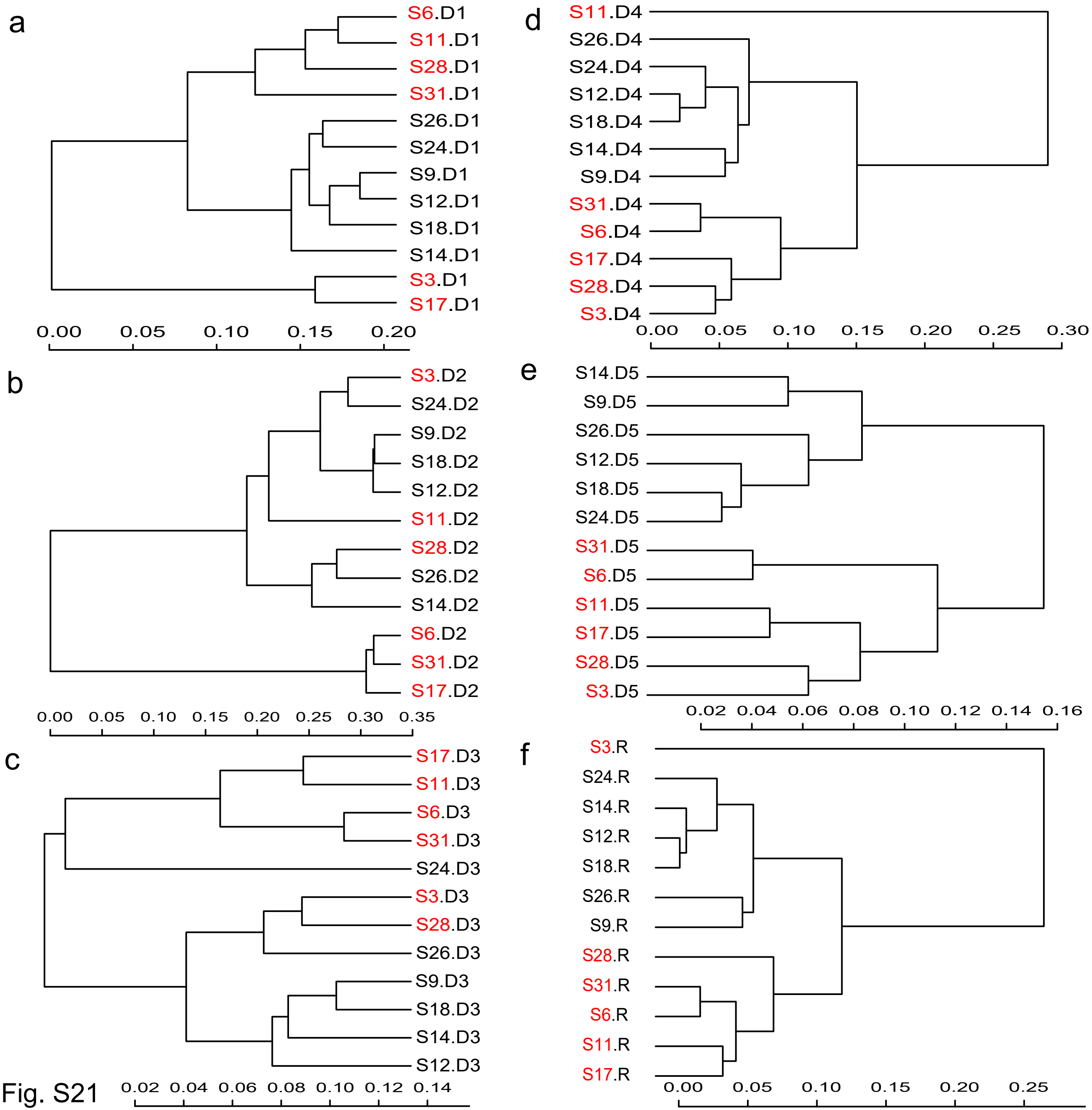
a
S6.D1
S11.D1
S28.D1
S31.D1
S26.D1
S24.D1
S9.D1
S12.D1
S18.D1
S14.D1
S3.D1
S17.D1
0.00 0.05 0.10 0.15 0.20
b
S3.D2
S24.D2
S9.D2
S18.D2
S12.D2
S11.D2
S28.D2
S26.D2
S14.D2
S6.D2
S31.D2
S17.D2
0.00 0.05 0.10 0.15 0.20 0.25 0.30 0.35
c
S17.D3
S11.D3
S6.D3
S31.D3
S24.D3
S3.D3
S28.D3
S26.D3
S9.D3
S18.D3
S14.D3
S12.D3
0.02 0.04 0.06 0.08 0.10 0.12 0.14
d
S11.D4
S26.D4
S24.D4
S12.D4
S18.D4
S14.D4
S9.D4
S31.D4
S6.D4
S17.D4
S28.D4
S3.D4
0.00 0.05 0.10 0.15 0.20 0.25 0.30
e
S14.D5
S9.D5
S26.D5
S12.D5
S18.D5
S24.D5
S31.D5
S6.D5
S11.D5
S17.D5
S28.D5
S3.D5
0.02 0.04 0.06 0.08 0.10 0.12 0.14 0.16
f
S3.R
S24.R
S14.R
S12.R
S18.R
S26.R
S9.R
S28.R
S31.R
S6.R
S11.R
S17.R
0.00 0.05 0.10 0.15 0.20 0.25

Fig. S21

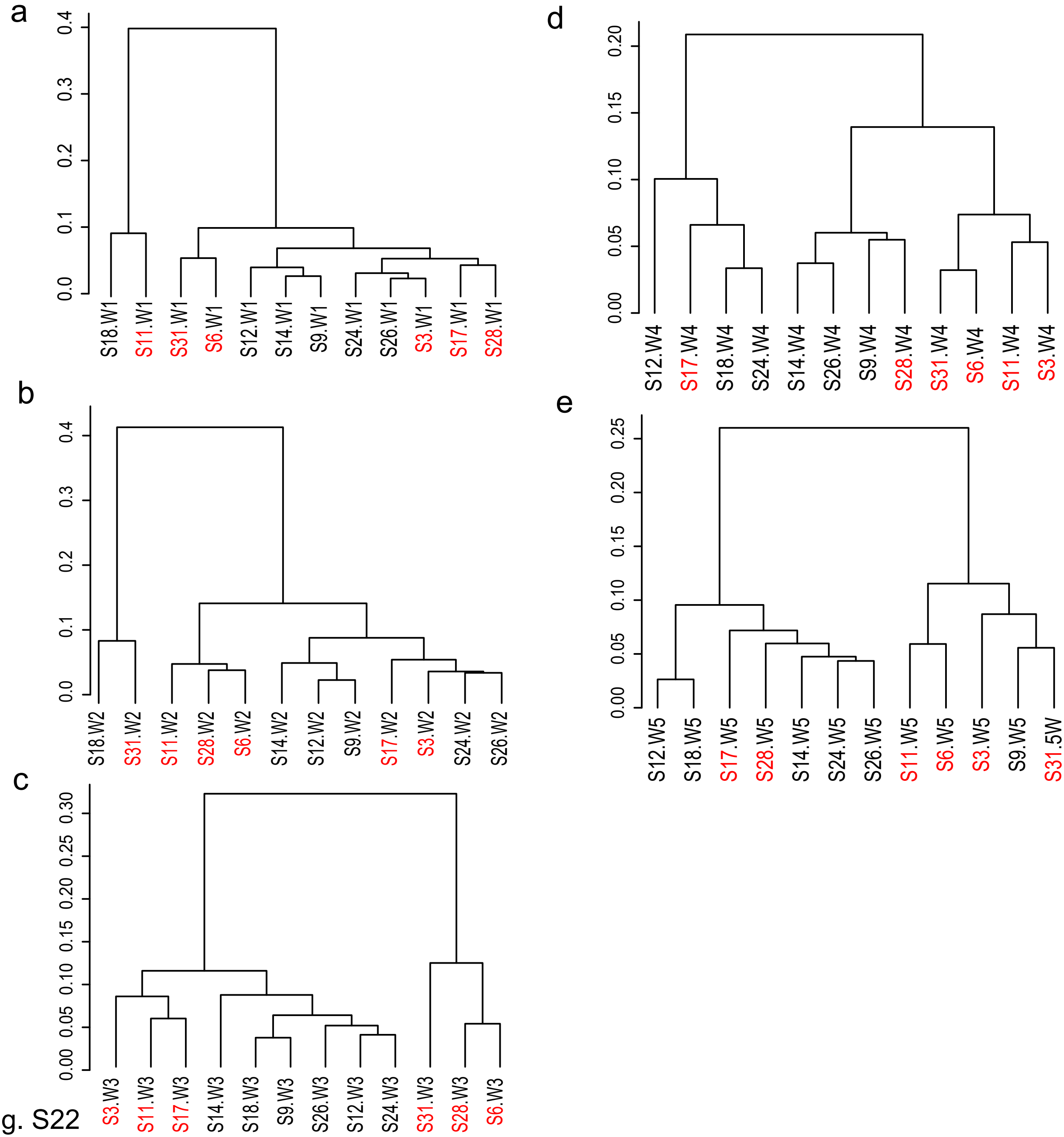
a
0.0
0.1
0.2
0.3
0.4
S18.W1
S11.W1
S31.W1
S6.W1
S12.W1
S14.W1
S9.W1
S24.W1
S26.W1
S3.W1
S17.W1
S28.W1
b
0.0
0.1
0.2
0.3
0.4
S18.W2
S31.W2
S11.W2
S28.W2
S6.W2
S14.W2
S12.W2
S9.W2
S17.W2
S3.W2
S24.W2
S26.W2
c
0.00
0.05
0.10
0.15
0.20
0.25
0.30
S3.W3
S11.W3
S17.W3
S14.W3
S18.W3
S9.W3
S26.W3
S12.W3
S24.W3
S31.W3
S28.W3
S6.W3
d
0.00
0.05
0.10
0.15
0.20
S12.W4
S17.W4
S18.W4
S24.W4
S14.W4
S26.W4
S9.W4
S28.W4
S31.W4
S6.W4
S11.W4
S3.W4
e
0.00
0.05
0.10
0.15
0.20
0.25
S12.W5
S18.W5
S17.W5
S28.W5
S14.W5
S24.W5
S26.W5
S11.W5
S6.W5
S3.W5
S9.W5
S31.5W
Fig. S22

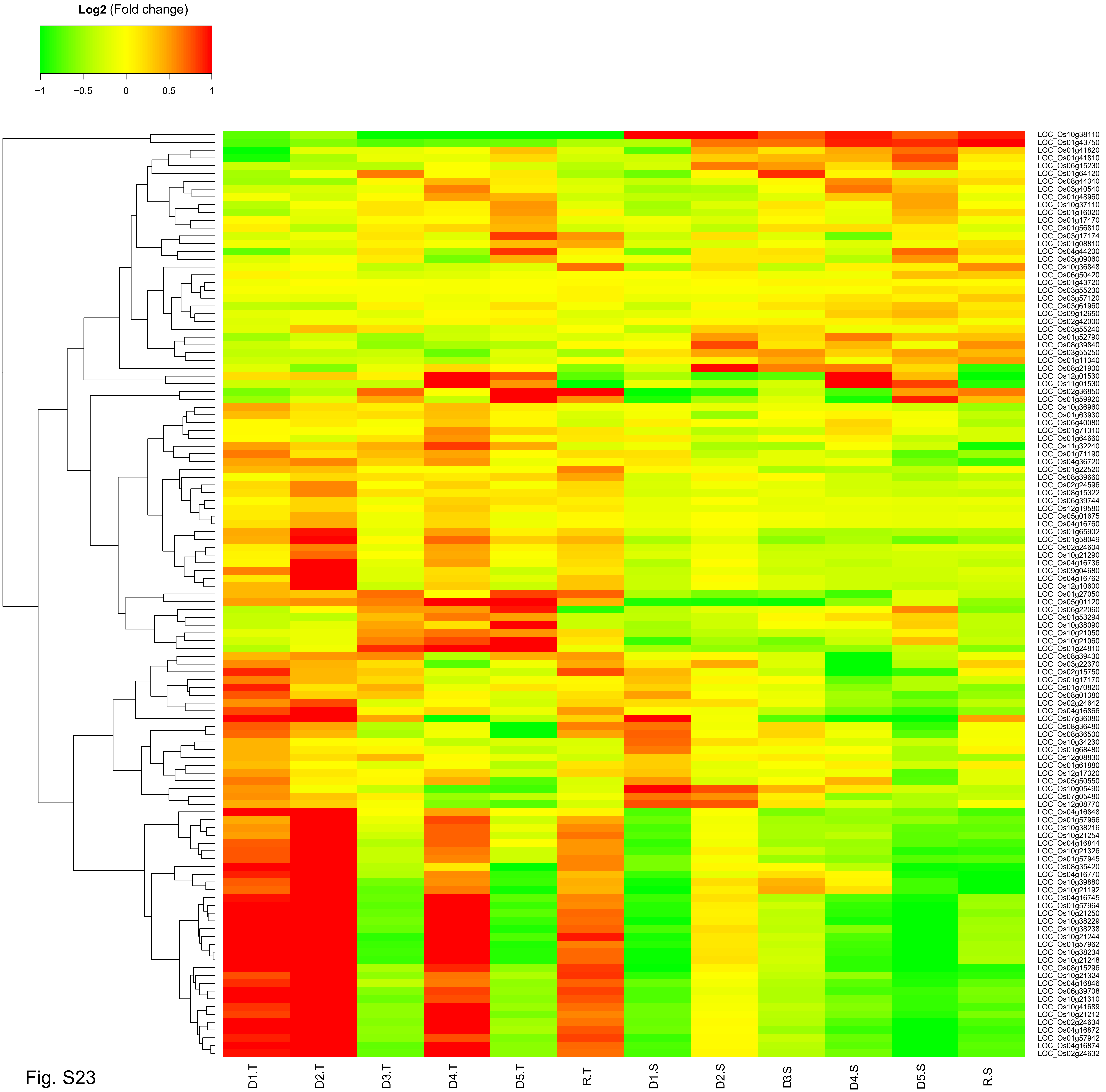

Log2 (Fold change)
−1
−0.5
0
0.5
1
D1.T
D2.T
D3.T
D4.T
D5.T
R.T
D1.S
D2.S
D3.S
D4.S
D5.S
R.S
LOC_Os10g38110
LOC_Os01g43750
LOC_Os01g41820
LOC_Os01g41810
LOC_Os06g15230
LOC_Os01g64120
LOC_Os08g44340
LOC_Os03g40540
LOC_Os01g48960
LOC_Os10g37110
LOC_Os01g16020
LOC_Os01g17470
LOC_Os01g56810
LOC_Os03g17174
LOC_Os01g08810
LOC_Os04g44200
LOC_Os03g09060
LOC_Os10g36848
LOC_Os06g50420
LOC_Os01g43720
LOC_Os03g55230
LOC_Os03g57120
LOC_Os03g61960
LOC_Os09g12650
LOC_Os02g42000
LOC_Os03g55240
LOC_Os01g52790
LOC_Os08g39840
LOC_Os03g55250
LOC_Os01g11340
LOC_Os08g21900
LOC_Os12g01530
LOC_Os11g01530
LOC_Os02g36850
LOC_Os01g59920
LOC_Os10g36960
LOC_Os01g63930
LOC_Os06g40080
LOC_Os01g71310
LOC_Os01g64660
LOC_Os11g32240
LOC_Os01g71190
LOC_Os04g36720
LOC_Os01g22520
LOC_Os08g39660
LOC_Os02g24596
LOC_Os08g15322
LOC_Os06g39744
LOC_Os12g19580
LOC_Os05g01675
LOC_Os04g16760
LOC_Os01g65902
LOC_Os01g58049
LOC_Os02g24604
LOC_Os10g21290
LOC_Os04g16736
LOC_Os09g04680
LOC_Os04g16762
LOC_Os12g10600
LOC_Os01g27050
LOC_Os05g01120
LOC_Os06g22060
LOC_Os01g53294
LOC_Os10g38090
LOC_Os10g21050
LOC_Os10g21060
LOC_Os01g24810
LOC_Os08g39430
LOC_Os03g22370
LOC_Os02g15750
LOC_Os01g17170
LOC_Os01g70820
LOC_Os08g01380
LOC_Os02g24642
LOC_Os04g16866
LOC_Os07g36080
LOC_Os08g36480
LOC_Os08g36500
LOC_Os10g34230
LOC_Os01g68480
LOC_Os12g08830
LOC_Os01g61880
LOC_Os12g17320
LOC_Os05g50550
LOC_Os10g05490
LOC_Os07g05480
LOC_Os12g08770
LOC_Os04g16848
LOC_Os01g57966
LOC_Os10g38216
LOC_Os10g21254
LOC_Os04g16844
LOC_Os10g21326
LOC_Os01g57945
LOC_Os08g35420
LOC_Os04g16770
LOC_Os10g39880
LOC_Os10g21192
LOC_Os04g16745
LOC_Os01g57964
LOC_Os10g21250
LOC_Os10g38229
LOC_Os10g38238
LOC_Os10g21244
LOC_Os01g57962
LOC_Os10g38234
LOC_Os10g21248
LOC_Os08g15296
LOC_Os10g21324
LOC_Os04g16846
LOC_Os06g39708
LOC_Os10g21310
LOC_Os10g41689
LOC_Os10g21212
LOC_Os02g24634
LOC_Os04g16872
LOC_Os01g57942
LOC_Os04g16874
LOC_Os02g24632

Fig. S23

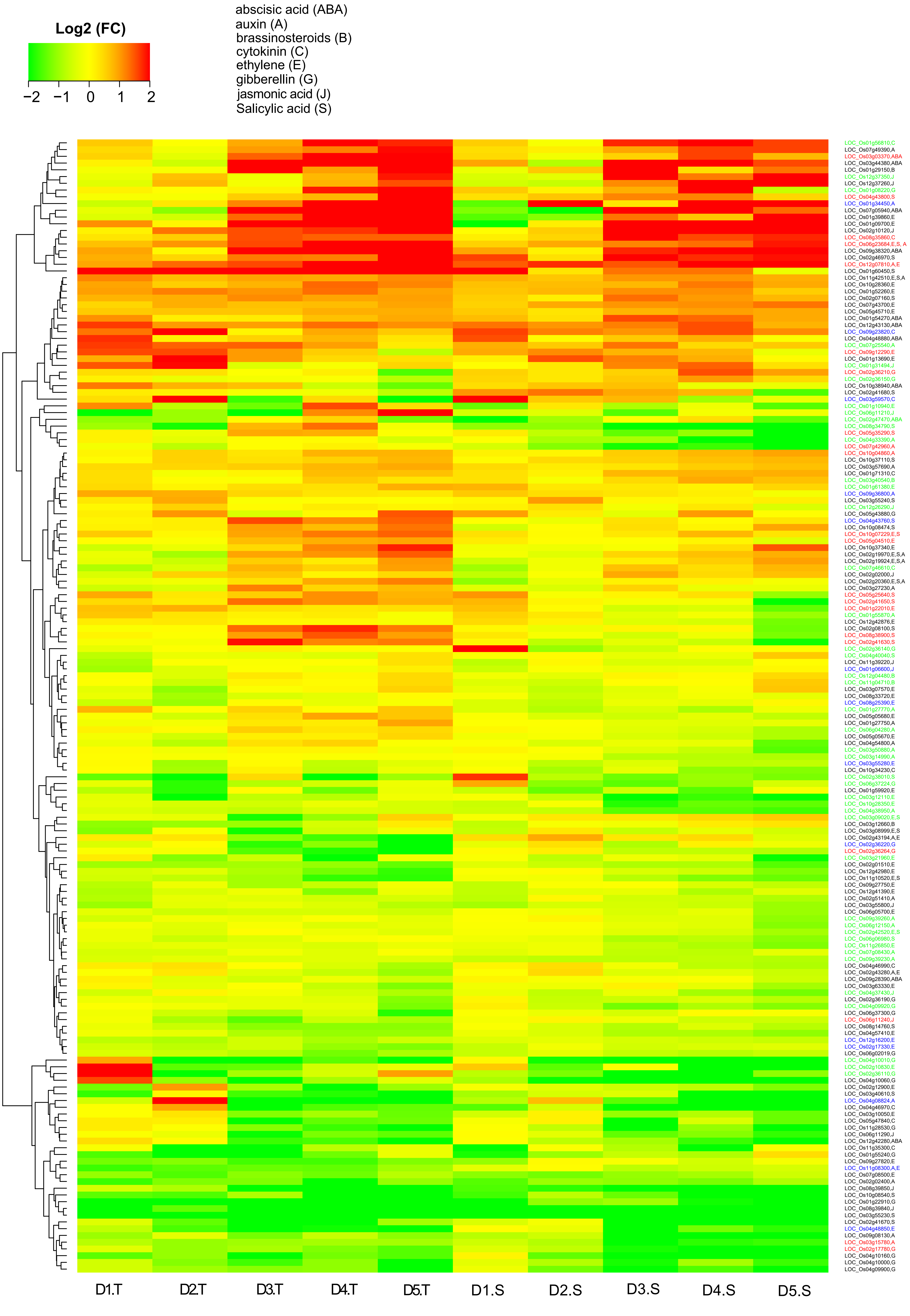
Log2 (FC)
−2
−1
0
1
2
abscisic acid (ABA)
auxin (A)
brassinosteroids (B)
cytokinin (C)
ethylene (E)
gibberellin (G)
jasmonic acid (J)
Salicylic acid (S)
D1.T
D2.T
D3.T
D4.T
D5.T
D1.S
D2.S
D3.S
D4.S
D5.S

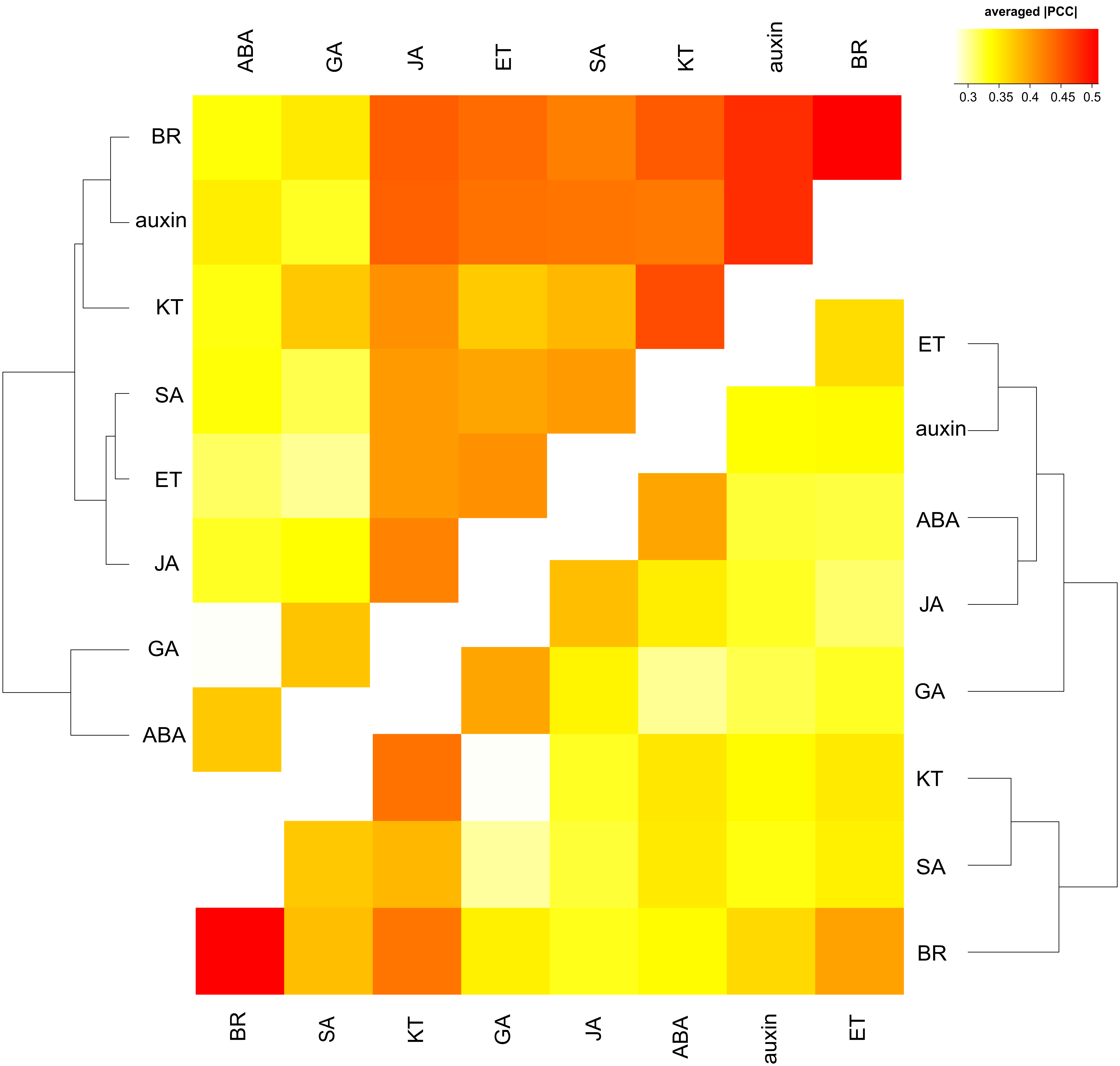

averaged |PCC|
0.3
0.35
0.4
0.45
0.5
ABA
GA
JA
ET
SA
KT
auxin
BR
BR
auxin
KT
SA
ET
JA
GA
ABA
ET
auxin
ABA
JA
GA
KT
SA
BR
BR
SA
KT
GA
JA
ABA
auxin
ET

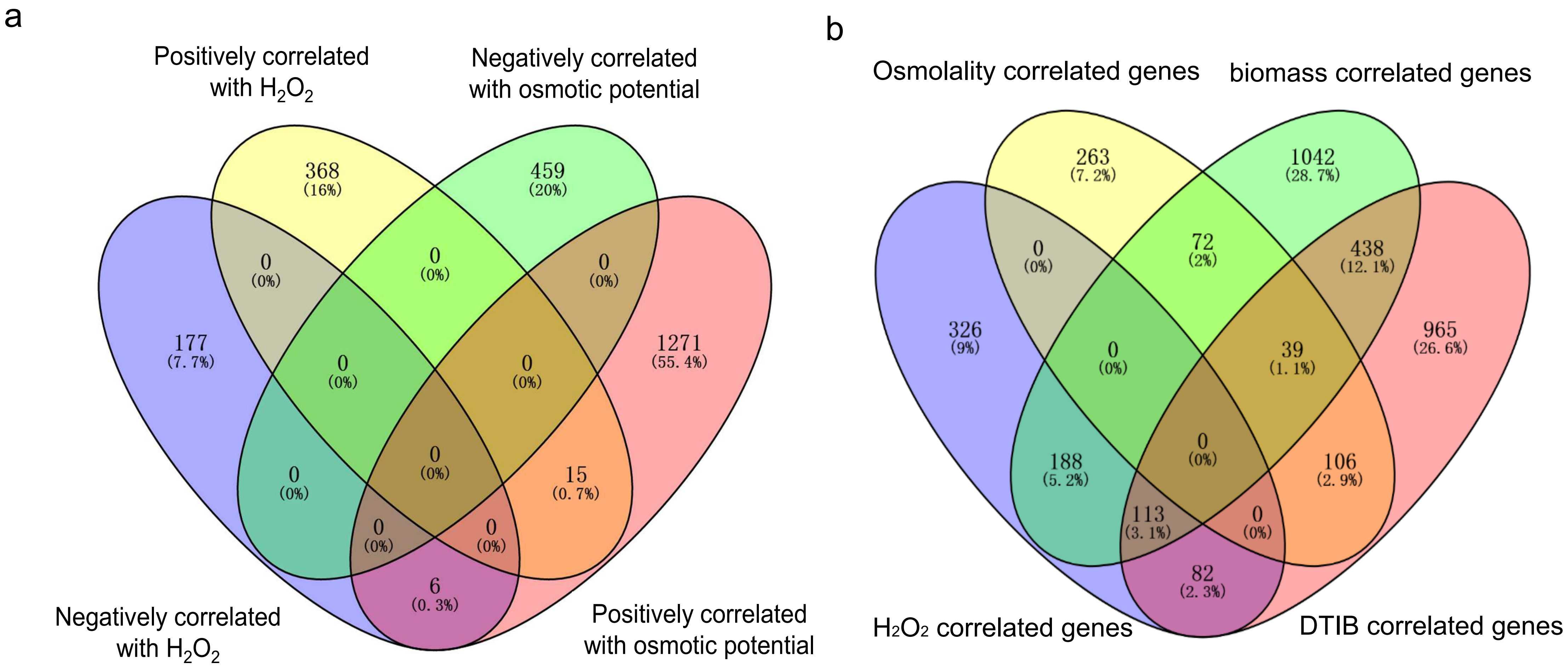
a
Positively correlated with H_2O_2
Negatively correlated with osmotic potential
Negatively correlated with H_2O_2
Positively correlated with osmotic potential
368 (16%)
459 (20%)
0 (0%)
0 (0%)
0 (0%)
177 (7.7%)
0 (0%)
0 (0%)
1271 (55.4%)
0 (0%)
0 (0%)
15 (0.7%)
0 (0%)
0 (0%)
6 (0.3%)
b
Osmolality correlated genes
biomass correlated genes
H_2O_2 correlated genes
DTIB correlated genes
263 (7.2%)
1042 (28.7%)
0 (0%)
72 (2%)
438 (12.1%)
326 (9%)
0 (0%)
39 (1.1%)
965 (26.6%)
0 (0%)
188 (5.2%)
106 (2.9%)
113 (3.1%)
0 (0%)
82 (2.3%)

Fig. S26

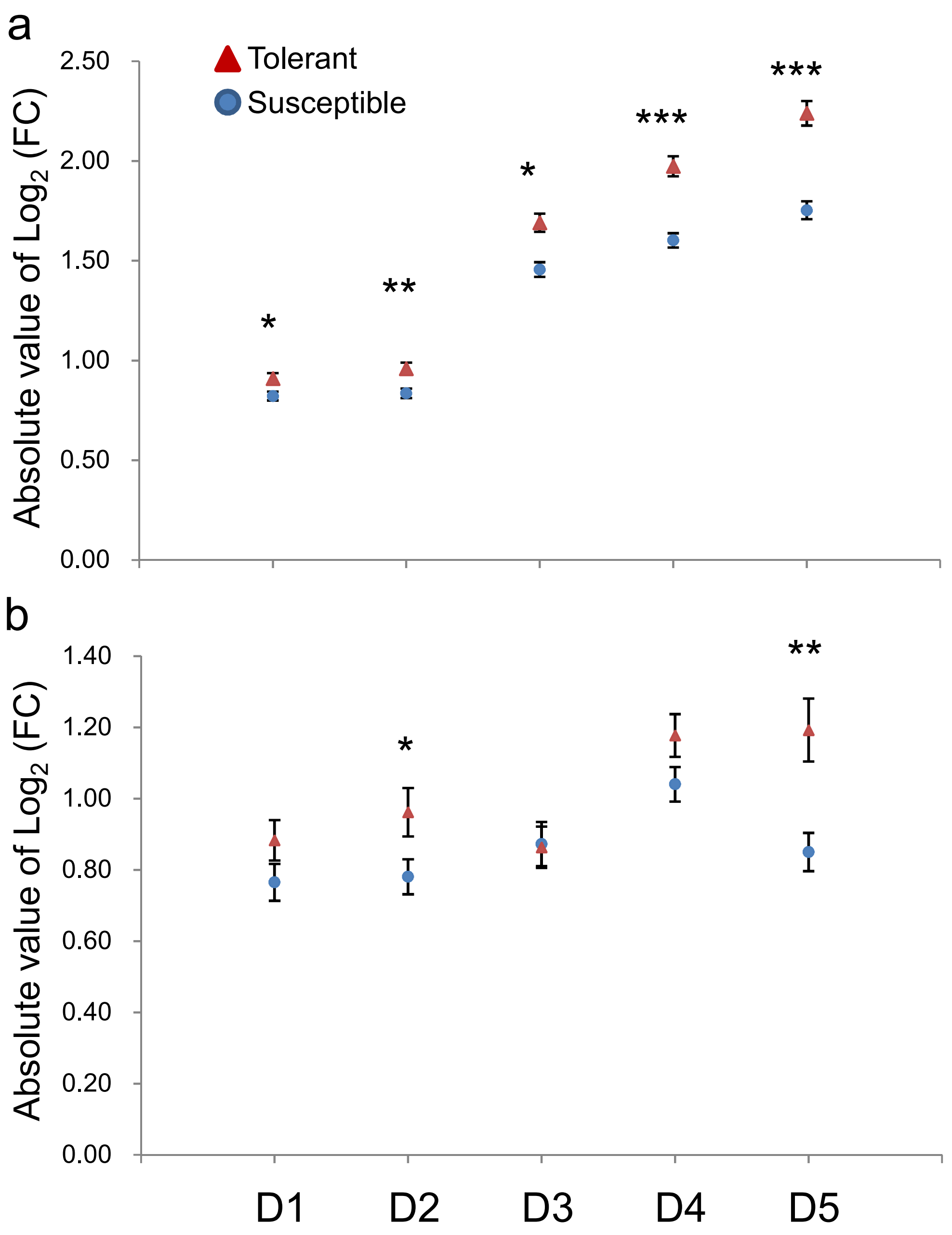
a
Tolerant
Susceptible
Absolute value of Log2 (FC)
2.50
2.00
1.50
1.00
0.50
0.00
*
**
*
***
***
b
Absolute value of Log2 (FC)
1.40
1.20
1.00
0.80
0.60
0.40
0.20
0.00
*
**
D1
D2
D3
D4
D5

Fig. S27
